# Supplementary material for: Second-Generation Enamine-Type Schiff Bases as 2-Amino Acid-Derived Antifungals against Fusarium oxysporum: Microwave-Assisted Synthesis, In Vitro Activity, 3D-QSAR, and In Vivo Effect
Source: J Fungi (Basel). 2023 Jan 13;9(1):113. doi: 10.3390/jof9010113 (PMC9866056; doi:10.3390/jof9010113)
Supplement: Supplementary file 1 [file jof-09-00113-s001.zip › jof-2139621-supplementary.pdf]

Supplementary Material

Research Article

Second-Generation Enamine-Type Schiff Bases as 2-Amino Acid-Derived Antifungals against *Fusarium Oxysporum*: Microwave-Assisted Synthesis, In-Vitro Activity, 3D-QSAR, and In-Vivo Effect

Paola Borrego-Muñoz, Diego Cardenas, Felipe Ospina, Ericsson Coy-Barrera and Diego Quiroga*

Content

Spectroscopic data for characterization of compounds 1-48 (Pages 1-7)

ATR-FT-IR spectra of selected compounds (Pages 8)

^{1}H and ^{13}C NMR spectra of compounds 1-48 (Pages 9-56)

HR-MS spectra of selected compounds (Pages 57-62)

Compounds 1-4, 17-20, and 33-36 showed the same properties and spectroscopic characterization than the previously reported by Borrego-Muñoz (2022, DOI: 10.1021/acsomega.2c02614)

Methyl (Z)-(4-oxopent-2-en-2-yl)-L-phenylalaninate (5, C15H19NO3)

^{1}H-NMR (400 MHz, $CDCl_3$) δ 11.11 (*d*, *J* = 9.5 Hz, 1H); 7.30 (*m*, 5H), 4.97 (*s*, 1H), 4.35 (*m*, 1H), 3.77 (*s*, 3H), 3.34 – 2.94 (*m*, 2H), 2.05 (*s*, 3H), 1.64 (*s*, 3H); ^{13}C-NMR (100 MHz, $CDCl_3$) 196.1, 171.6, 161.4, 136.2, 129.5, 128.8, 127.3, 96.8, 58.2, 52.7, 40.1, 29.2, 18.9; $[\alpha]_D^{25}$= -130.0° ± 0.01 (c 0.6, MeOH). ESI-HRMS m/z $[M+H]^+$, 262.1596 (calcd. 262.1443); ATR-FT-IR absorption bands in cm^{-1}: 1602 (C═C bond stretching), 1720 (C═O bond stretching), 2800–3430 cm–1 (N-H bond stretching).

Ethyl (Z)-(4-oxopent-2-en-2-yl)-L-phenylalaninate (6, C16H21NO3)

^{1}H-NMR (400 MHz, $CDCl_3$) δ 11.09 (*d*, *J* = 9.4 Hz, 1H), 7.39 – 7.18 (*m*, 5H), 4.96 (*s*, 1H), 4.32 (*m*, 1H), 4.21 (*m*, 2H), 3.24 – 2.99 (*m*, 2H), 2.04 (*s*, 3H), 1.65 (*s*, 3H), 1.27 (*t*, *J* = 7.2 Hz, 3H), ^{13}C-NMR (100 MHz, $CDCl_3$) δ 195.9, 171.1, 161.3, 136.3, 130.0, 128.7, 127.2, 96.7, 61.7, 58.3, 40.1, 29.1, 18.8, 14.2, $[\alpha]_D^{25}$= -96.67° ± 0.01 (c 0.6, MeOH). ATR-FT-IR absorption bands in cm^{-1}: 1604 (C═C bond stretching), 1712 (C═O bond stretching), 2810–3430 cm–1 (N-H bond stretching).

Isopropyl (Z)-(4-oxopent-2-en-2-yl)-L-phenylalaninate (7, C17H23NO3)

^{1}H-NMR ($CDCl_3$) *δ* 11.05 (*d*, *J* = 9,1 Hz, 1H), 7.19 – 7.34 (*m*, 5H), 5.01 (*m*, 1H), 4.94 (*s*, 1H), 4.26 (*m*, 1H), 3.15 (*m*, 1H), 2.99 (*m*, 1H), 1.63 (*s*, 3H), 2.01 (*s*, 3H), 1.21 (*m*, 6H), ^{13}C NMR ($CDCl_3$) *δ* 196.7, 170.5, 161.3, 136.2, 129.5, 128.6, 127.1, 96.6, 69.3, 58.3, 39.9, 29.0, 21.7, 21.7, 18.8, $[\alpha]_D^{25}$= -83,33° ± 0,01 (c 0,6, MeOH). ESI-HRMS m/z $[M+H]^+$, 290.1754 (calcd. 290.1751); ATR-FT-IR absorption bands in cm^{-1}: 1603 (C═C bond stretching), 1736 (C═O bond stretching), 2815–3440 cm–1 (N-H bond stretching).

Butyl (Z)-(4-oxopent-2-en-2-yl)-L-phenylalaninate (8, C18H25NO3)

^{1}H-NMR ($CDCl_3$) *δ* 10.98 (*d*, *J* = 9.1 Hz, 1H), 7.12 – 7.25 (*m*, 5H), 4.86 (*s*, 1H), 4.04 (*t*, J = 6.7 Hz, 2H), 4.22 (*m*, 1H), 3.10 (*m*, 1H), 2.92 (m, 1H), 1.55 (*s*, 3H), 1.94 (*s*, 2H), 1.46 – 1.55 (*m*, 2H), 1.25 (*m*, 2H); ^{13}C NMR ($CDCl_3$) *δ* 195.9, 171.1, 161.3, 136.2, 129.4, 128.7, 127.2, 96.7, 65.5, 58.2, 40.0, 30.5, 29.1, 19.0, 18.8, 13.7, $[\alpha]_D^{25}$= -144.17° ± 0.01 (c 0.6, MeOH); ESI-HRMS m/z $[M+H]^+$, 304.2143 (calcd. 304.1913); ATR-FT-IR absorption bands in cm^{-1}: 1604 (C═C bond stretching), 1738 (C═O bond stretching), 2810–3420 cm–1 (N-H bond stretching).

Methyl (Z)-(4-oxopent-2-en-2-yl)-L-tyrosinate (9, C15H19NO4)

^{1}H-NMR ($CDCl_3$) δ 10.99 (*d*, *J* = 9.0 Hz, 1H), 7.05 (*d*, *J* = 8.1 Hz, 2H), 6.77 (*d*, *J* = 8.1 Hz , 2H), 4.98 (*m*, 1H), 4.35 (m, 1H), 3.73 (*s*, 3H), 3.14 – 2.92 (*m*, 2H), 2.02 (*s*, 3H), 1.75 (*s*, 3H); ^{13}C NMR ($CDCl_3$) δ 196.3, 171.6, 162.7, 156.3, 130.6, 126.6, 115.9, 97.0, 58.2, 52.8, 38.7, 28.6, 19.2, $[\alpha]_D^{25}$= -100.00° ± 0.01 (c 0.6, MeOH). ESI-HRMS m/z $[M+H]^+$, 278.1674 (calcd. 278.1392); ATR-FT-IR absorption bands in cm^{-1}: 1604 (C═C bond stretching), 1711 (C═O bond stretching), 2800–3440 cm–1 (N-H bond stretching).

Ethyl (Z)-(4-oxopent-2-en-2-yl)-L-tyrosinate (10, $C_{16}H_{21}NO_4$)

^{1}H-NMR ($CDCl_3$) δ 11.00 (*d*, *J* = 8.7 Hz, 1H), 7.05 (*d*, *J* = 8.4 Hz, 2H), 6.78 (*d*, *J* = 8.4 Hz, 2H), 4.99 (*s*, 1H), 4.34 (*m*, 1H), 4.18 (*m*, 2H), 3.13 – 2.96 (*m*, 2H), 2.02 (*s*, 3H), 1.78 (*s*, 3H), 1.24 (*t*, *J* = 7.1 Hz, 3H); ^{13}C NMR ($CDCl_3$) δ 196.2, 171.0, 163.0, 156.6, 130.6, 126.2, 115.9, 96.9, 61.9, 58.2, 38.6, 28.5, 19.2, 14.2, $[\alpha]_D^{25}$= -

145.83° ± 0.01 (c 0.6, MeOH). ATR-FT-IR absorption bands in cm^{-1}: 1604 (C=C bond stretching), 1720 (C=O bond stretching), 2810–3430 cm–1 (N-H bond stretching).

Isopropyl (Z)-(4-oxopent-2-en-2-yl)-L-tyrosinate (11, $C_{17}H_{23}NO_4$)

^{1}H-NMR ($CDCl_3$) *δ* 11.06 (*d*, J = 8.8 Hz, 1H), 9.47 (*bs*, 1H), 7.05 (*d*, *J* = 8.4 Hz, 2H), 6.81 (*d*, *J* = 8.4 Hz, 2H), 5.02 (*m*, *J* = 6.3 Hz, 1H), 4.98 (*s*, 1H), 4.30 (*m*, 1H), 3.09 (*m*, 1H), 2.95 (*m*, 1H), 2.02 (*s*, 3H), 1.74 (*s*, 3H), 1.21 (*m*, 6H); ^{13}C NMR ($CDCl_3$) δ 195.9, 170.5, 163.1, 156.6, 130.5, 126.1, 115.8, 96.7, 69.6, 58.4, 38.8, 28.3, 21.7, 21.6, 19.1, $[\alpha]_D^{25}$= -223.33° ± 0.01 (c 0.6, MeOH). ESI-HRMS m/z $[M+H]^+$, 306.1704 (calcd. 306.1700); ATR-FT-IR absorption bands in cm^{-1}: 1597 (C=C bond stretching), 1733 (C=O bond stretching), 2810–3450 cm–1 (N-H bond stretching).

Butyl (Z)-(4-oxopent-2-en-2-yl)-L-tyrosinate (12, $C_{18}H_{25}NO_4$)

^{1}H-NMR ($CDCl_3$) *δ* 11.01 (*d*, *J* = 8.7 Hz, 1H), 9.30 (*bs*, 1H), 7.05 (*d*, *J* = 8.2 Hz, 2H), 6.80 (*d*, *J* = 8.2 Hz, 2H), 4.99 (*s*, 1H), 4.35 (*m*, 1H), 4.12 (*m*, 2H), 3.10 (*m*, 1H), 2.99 (*m*, 1H), 2.02 (*s*, 3H), 1.77 (*s*, 3H), 1.53 – 1.65 (*m*, 2H), 1.31 (*m*, 2H), 0.89 (*t*, *J* = 7.5 Hz, 3H); ^{13}C NMR ($CDCl_3$) *δ* 196.0, 171.1, 163.1, 156.7, 130.5, 126.0, 115.8, 96.8, 65.6, 58.3, 38.6, 30.5, 28.3, 19.1, 19.0, 13.7. $[\alpha]_D^{25}$= -183.33° ± 0.01 (c 0.6, MeOH). ESI-HRMS m/z $[M+H]^+$, 320.1862 (calcd. 320.1856); ATR-FT-IR absorption bands in cm^{-1}: 1596 (C=C bond stretching), 1731 (C=O bond stretching), 2810–3440 cm–1 (N-H bond stretching).

Methyl (Z)-(4-oxopent-2-en-2-yl)-L-alaninate (13, $C_9H_{15}NO_3$)

^{1}H-NMR ($CDCl_3$) δ 10.91 – 10.83 (*m*, 1H), 5.00 (*s*, 1H), 4.25 – 4.13 (*m*, 1H), 3.72 (*s*, 3H), 1.99 (*s*, 3H), 1.86 (*s*, 3H), 1.47 (*d*, *J* = 7.1 Hz, 3H); ^{13}C NMR ($CDCl_3$) δ 196.1, 172.8, 161.4, 96.6, 52.7, 51.5, 29.1, 19.2, 18.9, $[\alpha]_D^{25}$= -5.42° ± 0.01 (c 0.6, MeOH).

Ethyl (Z)-(4-oxopent-2-en-2-yl)-L-alaninate (14, $C_{10}H_{17}NO_3$)

^{1}H-NMR ($CDCl_3$) δ 10.88 (*d*, *J* = 7.8 Hz, 1H), 5.01 (*s*, 1H), 4.19 (*q*, *J* = 7.2 Hz, 3H), 2.01 (*s*, 3H), 1.88 (*s*, 3H), 1.48 (*d*, *J* = 7,1 Hz, 3H), 1.26 (*t*, *J* = 7.1 Hz, 3H); ^{13}C NMR ($CDCl_3$) δ 196.0, 172.3, 161.4, 96.6, 61.7, 51.7, 29.1, 19.2, 19.0, 14.2, $[\alpha]_D^{25}$= -4.17° ± 0.01 (c 0.6, MeOH).

Isopropyl (Z)-(4-oxopent-2-en-2-yl)-L-alaninate (15, $C_{11}H_{19}NO_3$)

^{1}H-NMR ($CDCl_3$) *δ* 10.88 (*d*, J = 7.2 Hz, 1H), 5.05 (*m*, 1H), 5.03 (*s*, 1H), 4.16 (*m*, 1H), 2.02 (*s*, 3H), 1.90 (*s*, 3H), 1.48 (*d*, *J* = 7.1 Hz, 3H), 1.26 (*d*, *J* = 6.2 Hz, 6H); ^{13}C NMR ($CDCl_3$) *δ* 195.8, 171.7, 161.3, 96.4, 69.2, 51.7, 29.0, 21.6, 21.6, 19.0, 18.9. $[\alpha]_D^{25}$= -2.58° ± 0.01 (c 0,6, MeOH).

Butyl (Z)-(4-oxopent-2-en-2-yl)-L-alaninate (16, $C_{12}H_{21}NO_3$)

^{1}H-NMR ($CDCl_3$) *δ* 10.89 (*d*, *J* = 7,8 Hz, 1H), 5.04 (*s*, 1H), 4.21 (*m*, 1H), 4.15 (*t*, *J* = 6,6 Hz, 2H), 2.02 (*s*, 3H), 1.91 (*s*, 3H), 1.63 (*m*, 2H), 1.50 (*d*, *J* = 7.1 Hz, 3H), 1.38 (*m*, 2H), 0.93 (*t*, *J* = 7.4 Hz, 3H); ^{13}C NMR ($CDCl_3$) *δ* 195.8, 172.3, 161.3, 96.5, 65.3, 51.6, 30.5, 29.0, 19.1, 19.0, 18.9, 13.6. $[\alpha]_D^{25}$= -2.17° ± 0.01 (c 0.6, MeOH).

Ethyl (S,Z)-3-((1-methoxy-1-oxo-3-phenylpropan-2-yl)amino)but-2-enoate (21, $C_{16}H_{21}NO_4$)

^{1}H-NMR ($CDCl_3$) δ 8.96 (*d*, *J* = 9.7 Hz, 1H), 7.37 – 7.24 (*m*, 5H), 4.50 (*s*, 1H), 4.33 (*m*, 1H), 4.16 (*q*, *J* = 7.1 Hz, 2H), 3.77 (*s*, 3H), 3.21 (*m*, 1H), 3.02 (*m*, 1H), 1.67 (*s*, 3H), 1.30 (*t*, *J* = 7.1 Hz, 3H); ^{13}C NMR ($CDCl_3$) δ 172.1, 170.3, 159.8, 136.5, 129.5, 128.8, 127.2, 85.0, 58.6, 52.5, 40.4, 19.3, 14.7, $[\alpha]_D^{25}$= -195.0° ± 0.01 (c 0.6,

MeOH). ATR-FT-IR absorption bands in cm^{-1}: 1601 (C═C bond stretching), 1720 (C═O bond stretching), 2820–3440 cm–1 (N-H bond stretching).

Ethyl (S,*Z*)-3-((1-ethoxy-1-oxo-3-phenylpropan-2-yl)amino)but-2-enoate (22, $C_{17}H_{23}NO_4$)

^{1}H-NMR ($CDCl_3$) δ 8.94 (*d*, *J* = 9.6 Hz, 1H), 7.38 – 7.22 (*m*, 5H), 4.49 (*m*, 1H), 4.30 (m, 1H), 4.18 (m, 3H), 3.26 – 2.97 (*m*, 2H), 1.68 (*s*, 3H), 1.28 (*m*, 6H); ^{13}C NMR ($CDCl_3$) δ 171.6, 170.3, 159.6, 136.5, 129.5, 128.7, 127.2, 84.9, 61.5, 58.6, 58.1, 40.4, 19.3, 14.7, 14.2, $[\alpha]_D^{25}$= -85.0° ± 0.01 (c 0.6, MeOH). ATR-FT-IR absorption bands in cm^{-1}: 1602 (C═C bond stretching), 1720 (C═O bond stretching), 2810–3450 cm–1 (N-H bond stretching).

Ethyl (S,*Z*)-3-((1-isopropoxy-1-oxo-3-phenylpropan-2-yl)amino)but-2-enoate (23, $C_{18}H_{25}NO_4$)

^{1}H-NMR ($CDCl_3$) *δ* 8.90 (*d*, *J* = 9.4 Hz, 1H), 7.18 – 7.33 (*m*, 4H), 5.01 (*q*, *J* = 6.3 Hz, 1H), 4.43 (*s*, 1H), 5.00 (*m*, *J* = 6.3 Hz, 1H), 4.22 (*m*, 1H), 3.36 – 3.48 (*m*, 1H), 3.12 (*m*, 1H), 2.97 (*m*, 1H), 1.65 (*s*, 3H), 1.23 (*t*, *J* = 6.3 Hz, 3H), 1.18 (*m*, 3H); ^{13}C NMR ($CDCl_3$) δ 171.1, 170.2, 159.9, 136.4, 129.4, 128.6, 127.0, 84.7, 69.1, 58.5, 58.0, 40.2, 21.6, 19.3, 14.6. $[\alpha]_D^{25}$= -53.25° ± 0.01 (c 0.6, MeOH). ATR-FT-IR absorption bands in cm^{-1}: 1603 (C═C bond stretching), 1721 (C═O bond stretching), 2810–3440 cm–1 (N-H bond stretching).

Ethyl (S,*Z*)-3-((1-butoxy-1-oxo-3-phenylpropan-2-yl)amino)but-2-enoate (24, $C_{19}H_{27}NO_4$)

^{1}H-NMR ($CDCl_3$) δ 8.91 (*m*, *J* = 9.6 Hz, 1H), 7.33 – 7.15 (*m*, 4H), 4.45 (*d*, *J* = 2.4 Hz, 1H), 4.26 (*m*, 1H), 4.15 – 4.04 (*m*, 8H), 3.21 – 2.91 (*m*, 4H), 2.04 (*s*, 3H), 1.61 – 1.48 (*m*, 4H), 1.37 – 1.19 (*m*, 4H), 0.90 (*m*, 9H); ^{13}C NMR ($CDCl_3$) δ 171.7, 170.2, 159.9, 136.4, 129.4, 128.6, 127.1, 84.8, 65.4, 60.5, 58.6, 58.0, 40.3, 30.5, 21.1, 19.3, 19.0, 14.6, 14.2, 13.7. $[\alpha]_D^{25}$= -77.13° ± 0.01 (c 0.6, MeOH). ATR-FT-IR absorption bands in cm^{-1}: 1604 (C═C bond stretching), 1720 (C═O bond stretching), 2810–3440 cm–1 (N-H bond stretching).

Ethyl (S,*Z*)-3-((3-(4-hydroxyphenyl)-1-methoxy-1-oxopropan-2-yl)amino)but-2-enoate (25, $C_{16}H_{21}NO_5$)

^{1}H-NMR ($CDCl_3$) δ 8.83 (*d*, *J* = 9.5 Hz, 1H), 6.99 (*d*, *J* = 8.1 Hz, 2H), 6.72 (*d*, *J* = 8.1 Hz, 2H), 4.46 (*m*, 1H), 4.26 (*m*, 1H), 4.10 (*m*, 2H), 3.70 (*s*, 2H), 3.70 (*s*, 2H), 3.08 – 2.88 (*m*, 2H), 1.67 (*s*, 2H), 1.24 (*t*, *J* = 7,2 Hz, 3H); ^{13}C NMR ($CDCl_3$), δ 172.2, 170.7, 160.4, 155.6, 130.5, 127.4, 115.7, 84.6, 77.2, 76.8, 60.6, 58.9, 58.0, 52.6, 39.4, 19.5, 14.6. $[\alpha]_D^{25}$= -14.92° ± 0.01 (c 0.6, MeOH). ATR-FT-IR absorption bands in cm^{-1}: 1602 (C═C bond stretching), 1738 (C═O bond stretching), 2820–3450 cm–1 (N-H bond stretching).

Ethyl (S,*Z*)-3-((1-ethoxy-3-(4-hydroxyphenyl)-1-oxopropan-2-yl)amino)but-2-enoate (26, $C_{17}H_{23}NO_5$)

^{1}H-NMR ($CDCl_3$) δ 8.86 (*d*, *J* = 9.5 Hz, 1H), 7.04 (*d*, *J* = 8.3 Hz, 2H), 6.73 (*d*, *J* = 8.1 Hz, 2H), 4.46 (*m*, 1H), 4.23 (*m*, 1H), 4.17 (*m*, 2H), 4.10 (*m*, 2H), 3.09 – 2.88 (*m*, 2H), 1.67 (*s*, 3H), 1.24 (*m*, 6H); ^{13}C NMR ($CDCl_3$) δ 171.8, 170.5, 160.2, 155.1, 130.7, 128.1, 115.6, 84.7, 77.5, 77.2, 76.8, 61.7, 58.8, 58.2, 39.4, 19.5, 14.7, 14.3, $[\alpha]_D^{25}$= -107.5° ± 0.01 (c 0.6, MeOH). ATR-FT-IR absorption bands in cm^{-1}: 1602 (C═C bond stretching), 1739 (C═O bond stretching), 2810–3440 cm–1 (N-H bond stretching).

Ethyl (S,*Z*)-3-((3-(4-hydroxyphenyl)-1-isopropoxy-1-oxopropan-2-yl)amino)but-2-enoate (27, $C_{18}H_{25}NO_5$)

^{1}H-NMR ($CDCl_3$) δ 8.82 (*d*, *J* = 9.1 Hz, 1H), 7.04 (*d*, *J* = 8.3 Hz, 2H), 6.71 (*d*, *J* = 8.3 Hz, 2H), 5.01 (*m*, 1H), 4.47 (*m*, 1H), 4.21 (*m*, 1H), 4.10 (*m*, 2H), 3.04 (*m*, 1H), 2.92 (*m*, 1H), 1.70 (*s*, 3H), 1.12 – 1.38 (*m*, 6H), 1.24 (*t*, *J* = 7.1 Hz, 1H), 1.21 (*m*, 6H); ^{13}C NMR ($CDCl_3$), δ 171.6, 171.4, 170.9, 170.6, 160.6, 160.5, 155.8, 155.5, 130.6, 69.4, 60.6, 58.9, 58.2, 50.8, 50.4, 39.2, 25.3, 21.8, 21.2, 19.5, 14.6, 14.3, $[\alpha]_D^{25}$= -56.67° ± 0.01 (c 0.6, MeOH). ATR-FT-IR absorption bands in cm^{-1}: 1603 (C═C bond stretching), 1729 (C═O bond stretching), 2820–3440 cm–1 (N-H bond stretching).

Ethyl (S,*Z*)-3-((1-butoxy-3-(4-hydroxyphenyl)-1-oxopropan-2-yl)amino)but-2-enoate (28, $C_{19}H_{27}NO_5$)

^{1}H-NMR ($CDCl_3$) δ 8.82 (*d*, *J* = 9.4 Hz, 1H), 7.02 (*d*, *J* = 8.3 Hz, 2H), 6.72 (*d*, *J* = 8.3 Hz, 2H), 4.48 (*s*, 1H), 4.25 (*m*, 1H), 4.11 (*t*, *J* = 6.7 Hz, 2H), 4.01 – 4.07 (*m*, 2H), 3.05 (*m*, 1H), 2.92 (*m*, 1H), 1.70 (*s*, 3H), 1.59 (*m*, 4H), 1.34 (*m*, 4H), 0.91 (*t*, *J* = 7.3 Hz, 6H); ^{13}C NMR ($CDCl_3$) δ 172.0, 170.8, 160.4, 155.5, 130.5, 127.3, 115.6, 84.6, 65.5, 62.9, 58.1, 39.2, 30.5, 19.5, 19.2, 19.0, 13.7. $[\alpha]_D^{25}$= -98.33° ± 0.01 (c 0.6, MeOH). ATR-FT-IR absorption bands in cm^{-1}: 1604 (C═C bond stretching), 1738 (C═O bond stretching), 2820–3440 cm–1 (N-H bond stretching).

Ethyl (S,Z)-3-((1-methoxy-1-oxopropan-2-yl)amino)but-2-enoate (29, $C_{10}H_{17}NO_4$)

^{1}H-NMR ($CDCl_3$) δ 8.75 (*d*, *J* = 8.7 Hz, 1H), 4.53 (*s*, 1H), 4.19 (*m*, 2H), 4.10 (*q*, *J* = 7.1 Hz, 1H), 3.74 (*s*, 3H), 2.27 (*s*, 3H), 1.48 (*d*, *J* = 7.0 Hz, 3H), 1.25 (*t*, 3H); ^{13}C NMR ($CDCl_3$) δ 200.7, 173.4, 170.8, 167.7, 160.2, 84.2, 52.7, 52.5, 51.5, 50.3, 50.0, 30.3, 19.5, $[\alpha]_D^{25}$= 25.00° ± 0.01 (c 0.6, MeOH).

Ethyl (S,Z)-3-((1-ethoxy-1-oxopropan-2-yl)amino)but-2-enoate (30, $C_{11}H_{19}NO_4$)

^{1}H-NMR ($CDCl_3$) δ 7.46 (*d*, *J* = 7.4 Hz, 1H), 4.48 (*q*, *J* = 7.2 Hz, 1H), 4.12 (*q*, *J* = 7.0 Hz, 4H), 3.39 (*s*, 1H), 2.21 (*s*, 3H), 1.35 (*d*, *J* = 7.2 Hz, 3H), 1.21 (*t*, *J* = 7.1 Hz, 6H); ^{13}C NMR ($CDCl_3$) δ 204.0, 172.7, 165.5, 61.5, 49.8, 48.2, 30.8, 18.0, 14.1, $[\alpha]_D^{25}$= -55.00° ± 0.01 (c 0.6, MeOH).

Ethyl (S,Z)-3-((1-isopropoxy-1-oxopropan-2-yl)amino)but-2-enoate (31, $C_{12}H_{21}NO_4$)

^{1}H-NMR ($CDCl_3$) δ 8.70 (*d*, *J* = 8.7 Hz, 1H), 5.00 (*m*, 1H), 4.47 (*s*, 1H), 4.06 (*m*, 2H), 1.84 (*s*, 2H), 1.41 (*d*, *J* = 7.0 Hz, 2H), 1.24 – 1.17 (*m*, 9H); ^{13}C NMR ($CDCl_3$) δ 172.4, 170.3, 160.1, 84.4, 69.0, 58.5, 51.6, 21.7, 19.4, 19.3, 14.6, $[\alpha]_D^{25}$= 40.00° ± 0.01 (c 0.6, MeOH).

Ethyl (S,Z)-3-((1-butoxy-1-oxopropan-2-yl)amino)but-2-enoate (32, $C_{13}H_{23}NO_4$)

^{1}H-NMR ($CDCl_3$) *δ* 8.75 (*d*, *J* = 8.3 Hz, 1H), 4.53 (*s*, 1H), 4.15 (*m*, 1H), 4.10 (*q*, *J* = 7.1 Hz, 2H), 1.89 (*s*, 3H), 1.58 – 1.67 (*m*, 2H), 1.48 (*d*, *J* = 7.0 Hz, 3H), 1.37 (*m*, 2H), 1.25 (*t*, *J* = 7.1 Hz, 3H), 0.93 (*t*, *J* = 7.4 Hz, 4H); ^{13}C NMR ($CDCl_3$) *δ* 173.0, 170.36, 160.1, 84.5, 65.3, 58.6, 51.5, 30.6, 19.4, 19.4, 19.0, 14.6, 13.7. $[\alpha]_D^{25}$= 23.33° ± 0.01 (c 0.6, MeOH).

Methyl (3-oxocyclohex-1-en-1-yl)-L-phenylalaninate (37, $C_{16}H_{19}NO_3$)

^{1}H-NMR ($CDCl_3$) δ 7.30 – 7.21 (*m*, 1H), 7.07 – 6.99 (*m*, 1H), 5.13 (*s*, 1H), 5.02 (*d*, *J* = 7.6 Hz, 1H), 4.33 (*m*, 1H), 3.71 (*s*, 2H), 3.22 – 3.00 (*m*, 2H), 2.31 (*m*, 2H), 1.95 (*m*, 1H), 1.23 (*d*, *J* = 8.6 Hz, 3H); ^{13}C NMR ($CDCl_3$) δ 197.6, 171.7, 162.5, 135.3, 129.3, 128.8, 127.4, 98.4, 55.8, 52.6, 37.2, 36.5, 29.7, 21.9, $[\alpha]_D^{25}$= -10.83° ± 0.01 (c 0.6, MeOH). ESI-HRMS m/z $[M+H]^+$, 274.1443 (calcd. 274.1438); ATR-FT-IR absorption bands in cm^{-1}: 1597 (C═C bond stretching), 1736 (C═O bond stretching), 3266 cm–1 (N-H bond stretching).

Ethyl (3-oxocyclohex-1-en-1-yl)-L-phenylalaninate (38, $C_{17}H_{21}NO_3$)

^{1}H-NMR ($CDCl_3$) δ 7.34 – 7.03 (*m*, 5H), 5.16 (*s*, 1H), 4.95 (*d*, *J* = 7.5 Hz, 2H), 4.32 (*q*, *J* = 6.3 Hz, 1H), 4.16 (*q*, *J* = 7.2 Hz, 2H), 3.22 – 3.02 (*m*, 2H), 2.32 (*t*, *J* = 6.4 Hz, 4H), 1.96 (*m*, 2H), 1.23 (*t*, *J* = 7.1 Hz, 3H); ^{13}C NMR ($CDCl_3$) δ 197.7, 171.3, 162.4, 135.4, 129.4, 128.7, 127.4, 98.4, 62.0, 55.8, 37.2, 36.5, 29.8, 22.0, 14.2, $[\alpha]_D^{25}$= -21.89° ± 0.01 (c 0.6, MeOH). ATR-FT-IR absorption bands in cm^{-1}: 1597 (C═C bond stretching), 1736 (C═O bond stretching), 3266 cm–1 (N-H bond stretching).

Isopropyl (3-oxocyclohex-1-en-1-yl)-L-phenylalaninate (39, $C_{18}H_{23}NO_3$)

^{1}H-NMR ($CDCl_3$) *δ* 7.21 – 7.31 (*m*, 3H), 7.11 (*m*, 2H), 5.47 (*d*, *J* = 7.0 Hz, 1H), 5.16 (*s*, 1H), 4.99 (*m*, 1H), 4.29 (*m*, 1H), 3.16 (*m*, 1H), 3.08 (*m*, 1H), 2.35 (*t*, *J* = 6.1 Hz, 2H), 2.31 (*t*, *J* = 6.4 Hz, 2H), 1.95 (*m*, 2H), 1.23 (*m*, 6H); ^{13}C NMR ($CDCl_3$) δ 197.7, 170.8, 163.1, 135.4, 129.3, 128.5, 127.2, 97.8, 69.8, 56.0, 37.0, 36.4, 29.6, 21.8, 21.8, 21.6. $[\alpha]_D^{25}$= -12,50° ± 0,01 (c 0,6, MeOH). ESI-HRMS m/z $[M+H]^+$, 302.1982 (calcd. 302.1756); ATR-FT-IR absorption bands in cm^{-1}: 1600 (C═C bond stretching), 1739 (C═O bond stretching), 3266 cm–1 (N-H bond stretching).

Butyl (3-oxocyclohex-1-en-1-yl)-L-phenylalaninate (40, $C_{19}H_{25}NO_3$)

^{1}H-NMR ($CDCl_3$) *δ* 7.23 – 7.31 (*m*, 3H), 7.09 (*d*, *J* = 7.2 Hz, 2H), 5.49 (*d*, *J* = 7.2 Hz, 1H), 5.15 (*s*, 1H), 4.33 (*m*, 1H), 4.09 (*m*, 2H), 3.17 (*m*, 1H), 3.09 (*m*, 1H), 2.35 (*t*, *J* = 6.1 Hz, 2H), 2.31 (*t*, *J* = 6.4 Hz, 2H), 1.95 (*m*, 2H), 1.51 – 1.60 (*m*, 2H), 1.39 (*m*, 2H), 0.91 (*t*, *J* = 7.4 Hz, 3H); ^{13}C NMR ($CDCl_3$) *δ* 197.7, 171.4, 163.1, 135.4, 129.2, 128.6, 127.2, 97.8, 65.6, 56.0, 37.1, 36.4, 30.4, 29.5, 21.8, 19.0, 13.7. $[\alpha]_D^{25}$= -9.27° ± 0.01 (c 0.6, MeOH). ESI-HRMS m/z $[M+H]^+$, 316.2156 (calcd. 316.1913); ATR-FT-IR absorption bands in cm^{-1}: 1594 (C═C bond stretching), 1737 (C═O bond stretching), 3060 cm–1 (N-H bond stretching).

Methyl (3-oxocyclohex-1-en-1-yl)-L-tyrosinate (41, $C_{16}H_{19}NO_4$)

^{1}H-NMR ($CDCl_3$) δ 7.29 (*s*, 1H), 6.86 (*d*, *J* = 8.2 Hz, 2H), 6.78 (*d*, *J* = 8.2 Hz, 2H), 5.44 (*d*, *J* = 7.4 Hz, 1H), 5.11 (*s*, 1H), 4.26 (*q*, *J* = 6.1 Hz, 1H), 3.71 (*s*, 3H), 2.99 (*m*, 2H), 2.39 – 2.28 (*m*, 4H), 1.94 (*m*, 2H); ^{13}C NMR ($CDCl_3$) δ 198.8, 171.7, 164.6, 156.4, 130.2, 125.7, 115.9, 104.5, 97.4, 60.6, 56.1, 52.7, 36.4, 35.9, 29.7, 21.2, $[\alpha]_D^{25}$= 5.83° ± 0.01 (c 0.6, MeOH). ESI-HRMS m/z $[M+H]^+$, 290.1392 (calcd. 290.1387); ATR-FT-IR absorption bands in cm^{-1}: 1594 (C═C bond stretching), 1731 (C═O bond stretching), 3064 cm–1 (N-H bond stretching).

Ethyl (3-oxocyclohex-1-en-1-yl)-L-tyrosinate (42, $C_{17}H_{21}NO_4$)

^{1}H-NMR ($CDCl_3$) δ 7.26 (*s*, 1H), 6.84 (*d*, *J* = 8.1 Hz, 2H), 6.76 (*d*, *J* = 8.2 Hz, 1H), 5.30 (*d*, *J* = 7.5 Hz, 1H), 5.13 (*s*, 1H), 4.22 (*q*, J = 6,1 Hz, 1H), 4.17 – 4.06 (*m*, 2H), 3.70 (*q*, *J* = 7.1 Hz, 2H), 2.98 (*m*, 2H), 2.31 (*d*, *J* = 6.5 Hz, 2H), 1.92 (*m*, 1H), 1.22 (*m*, 3H); ^{13}C NMR ($CDCl_3$) δ 198.7, 171.3, 164.2, 156.4, 130.3, 125.6, 115.8, 97.5, 62.0, 60.6, 58.4, 56.0, 36.2, 29.8, 21.8, 21.2, 18.4, 14.2. $[\alpha]_D^{25}$=7.50° ± 0.01 (c 0.6, MeOH). ATR-FT-IR absorption bands in cm^{-1}: 1594 (C═C bond stretching), 1732 (C═O bond stretching), 3064 cm–1 (N-H bond stretching).

Isopropyl (3-oxocyclohex-1-en-1-yl)-L-tyrosinate (43, $C_{18}H_{23}NO_4$)

^{1}H-NMR ($CDCl_3$) *δ* 6.89 (*d*, *J* = 8.3 Hz, 2H), 6.79 (*d*, *J* = 8.3 Hz, 2H), 5.58 (*d*, *J* = 6.9 Hz, 1H), 5.15 (*s*, 1H), 4.99 (*m*, 1H), 4.22 (*m*, 1H), 3.04 (*m*, 1H), 2.95 (*m*, 1H), 2.29 – 2.41 (*m*, 4H), 1.89 – 1.97 (*m*, 2H), 1.21 (*m*, 6H); ^{13}C NMR ($CDCl_3$) *δ* 198.8, 170.8, 164.9, 156.4, 130.3, 125.7, 115.8, 97.2, 70.0, 56.2, 36.2, 35.8, 29.6,

21.7, 21.7. $[\alpha]_D^{25}$= -4,17° ± 0,01 (c 0,6, MeOH). ESI-HRMS m/z $[M+H]^+$, 318.1702 (calcd. 318.1700); ATR-FT-IR absorption bands in cm^{-1}: 1595 (C═C bond stretching), 1741 (C═O bond stretching), 3050 cm–1 (N-H bond stretching).

Butyl (3-oxocyclohex-1-en-1-yl)-L-tyrosinate (44, $C_{19}H_{25}NO_4$)

^{1}H-NMR ($CDCl_3$) δ 6.87 (*d*, *J* = 8.4 Hz, 2H), 6.78 (*d*, *J* = 8.4 Hz, 2H), 5.40 (*d*, *J* = 7.0 Hz, 1H), 5.12 (*s*, 1H), 4.27 (*m*, 1H), 4.11 (*t*, *J* = 6.8 Hz, 2H), 3.06 (*m*, 1H), 2.94 (*m*, 1H), 2.29 – 2.36 (*m*, 4H), 1.89 – 1.97 (*m*, 2H), 1.59 (*m*, 2H), 1.34 (*m*, 2H), 0.92 (*t*, *J* = 7.4 Hz, 3H); ^{13}C NMR ($CDCl_3$) *δ* 198.8, 171.3, 164.6, 156.4, 130.3, 125.7, 115.8, 97.3, 65.9, 56.1, 36.4, 35.8, 30.4, 29.7, 21.7, 19.1, 13.7. $[\alpha]_D^{25}$= -4.17° ± 0.01 (c 0.6, MeOH). ESI-HRMS m/z $[M+Na]^+$, 354.1677 (calcd. 354.1676); ATR-FT-IR absorption bands in cm^{-1}: 1594 (C═C bond stretching), 1738 (C═O bond stretching), 3050 cm–1 (N-H bond stretching).

Methyl (3-oxocyclohex-1-en-1-yl)-L-alaninate (45, $C_{10}H_{15}NO_3$)

^{1}H-NMR ($CDCl_3$) δ 5.17 (*s*, 1H), 5.02 (*s*, 1H), 4.07 (*s*, 1H), 3.75 (*s*, 3H), 2.39 – 2.26 (*m*, 4H), 1.95 (*m*, 2H), 1.43 (*s*, 1H); ^{13}C NMR ($CDCl_3$) δ 197.7, 173.2, 162.7, 97.8, 52.8, 50.4, 36.4, 29.7, 21.8, 17.9, $[\alpha]_D^{25}$= -7.69° ± 0.01 (c 0.6, MeOH).

Ethyl (3-oxocyclohex-1-en-1-yl)-L-alaninate (46, $C_{11}H_{17}NO_3$)

^{1}H-NMR ($CDCl_3$) δ 5.05 (*s*, 1H), 5.03 (*s*, 1H), 4.23 (*q*, *J* = 7.1 Hz, 2H), 4.06 (*m*, 1H), 2.41 – 2.27 (*m*, 4H), 1.97 (*m*, 2H), 1.43 (*d*, *J* = 6.9 Hz, 3H), 1.30 (*t*, *J* = 7.1 Hz, 3H); ^{13}C NMR ($CDCl_3$) δ 197.7, 173.3, 162.4, 98.0, 62.0, 50.5, 36.4, 29.8, 21.9, 18.0, 14.2. $[\alpha]_D^{25}$= -15.62° ± 0.01 (c 0.6, MeOH),

Isopropyl (3-oxocyclohex-1-en-1-yl)-L-alaninate (47, $C_{12}H_{19}NO_3$)

^{1}H-NMR ($CDCl_3$) *δ* 5.75 (*d*, *J* = 6.7 Hz, 1H), 5.06 (*m*, 1H), 5.04 (*s*, 1H), 4.04 (*m*, 1H), 2.41 (*t*, *J* = 6.2 Hz, 2H), 2.31 (*t*, *J* = 6.4 Hz, 2H), 1.97 (*m*, 2H), 1.27 (*m*, 6H); ^{13}C NMR ($CDCl_3$) δ 197.7, 172.2, 163.5, 97.3, 69.5, 50.6, 36.3, 29.5, 21.8, 21.7, 21.6, 17.7. $[\alpha]_D^{25}$= -22.50° ± 0.01 (c 0.6, MeOH).

Butyl (3-oxocyclohex-1-en-1-yl)-L-alaninate (48, $C_{13}H_{21}NO_3$)

^{1}H-NMR ($CDCl_3$) *δ* 5.68 (*d*, *J* = 6.8 Hz, 1H), 5.05 (*s*, 1H), 4.10 – 4.23 (*m*, 2H), 4.09 (*m*, 1H), 2.41 (*t*, *J* = 6.2 Hz, 2H), 2.31 (*t*, *J* = 6.4 Hz, 2H), 1.97 (*m*, 2H), 1.60 – 1.69 (*m*, 2H), 1.45 (*d*, *J* = 7.0 Hz, 3H), 1.39 (*m*, 2H), 0.94 (*t*, *J* = 7.4 Hz, 3H); ^{13}C NMR ($CDCl_3$) *δ* 197.7, 172.8, 163.4, 97.4, 65.6, 50.6, 36.4, 30.4, 29.5, 21.8, 19.0, 17.8, 13.6. $[\alpha]_D^{25}$= -104.17° ± 0.01 (c 0.6, MeOH).

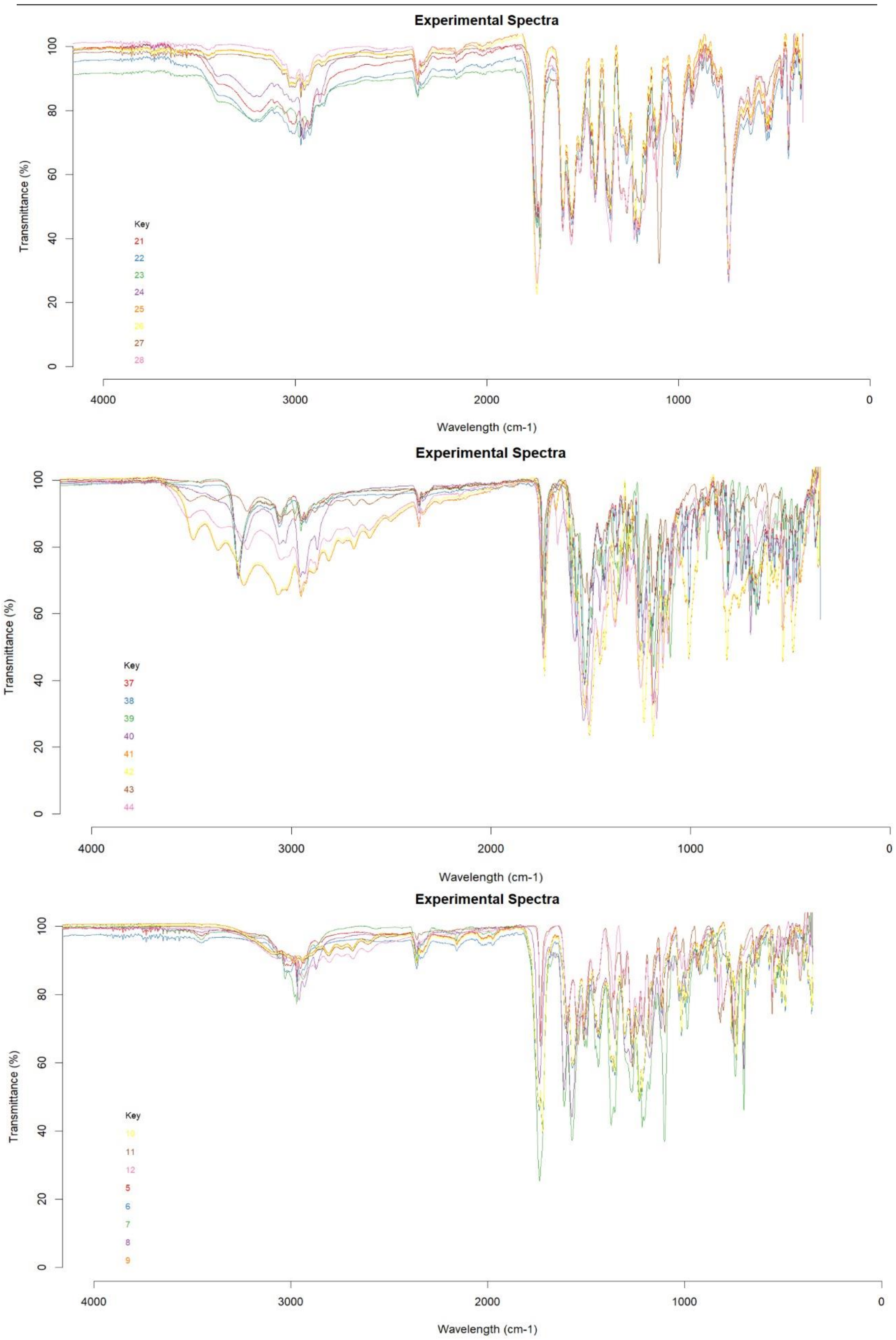

ATR-FT-IR spectra of selected compounds

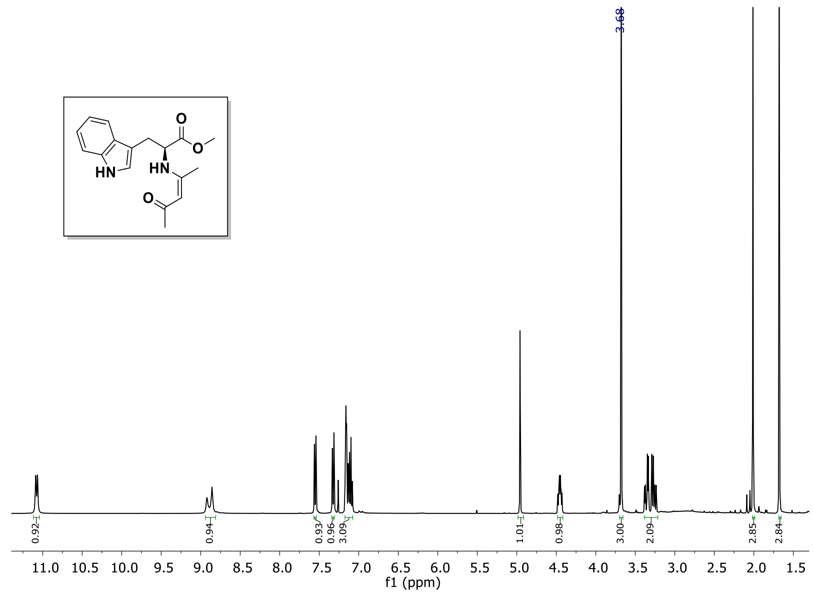

1H NMR spectrum of 1 in CDCl3.

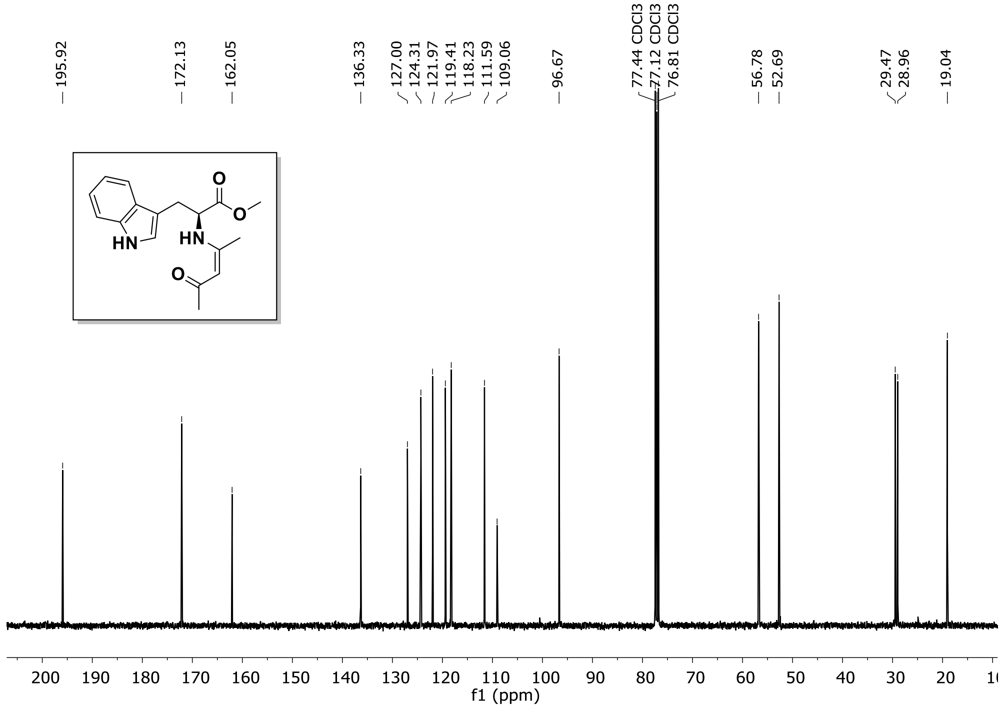

13C NMR spectrum of 1 in CDCl3.

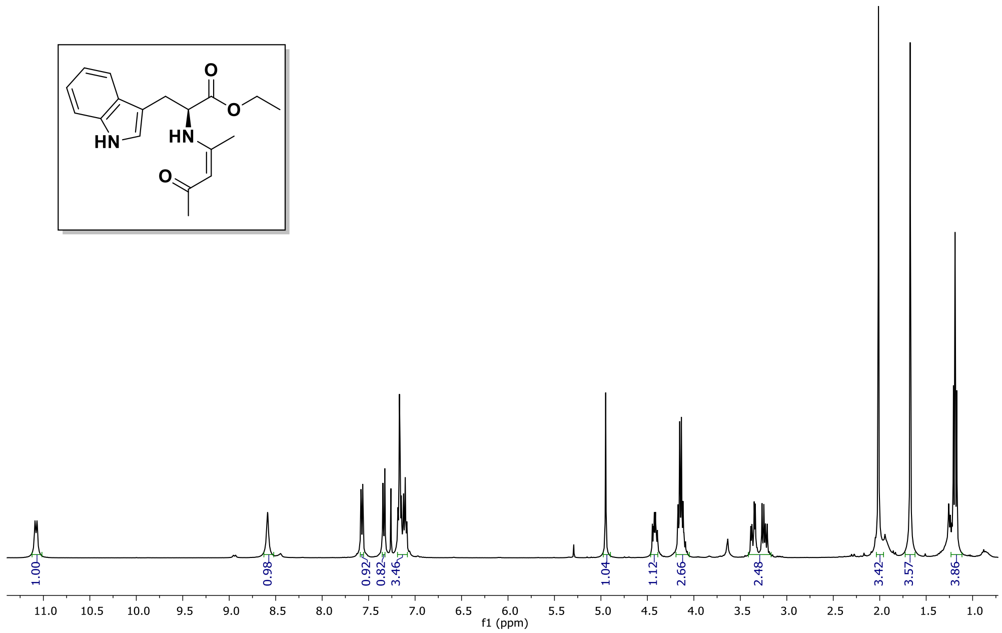

1H NMR spectrum of 2 in $CDCl_3$.

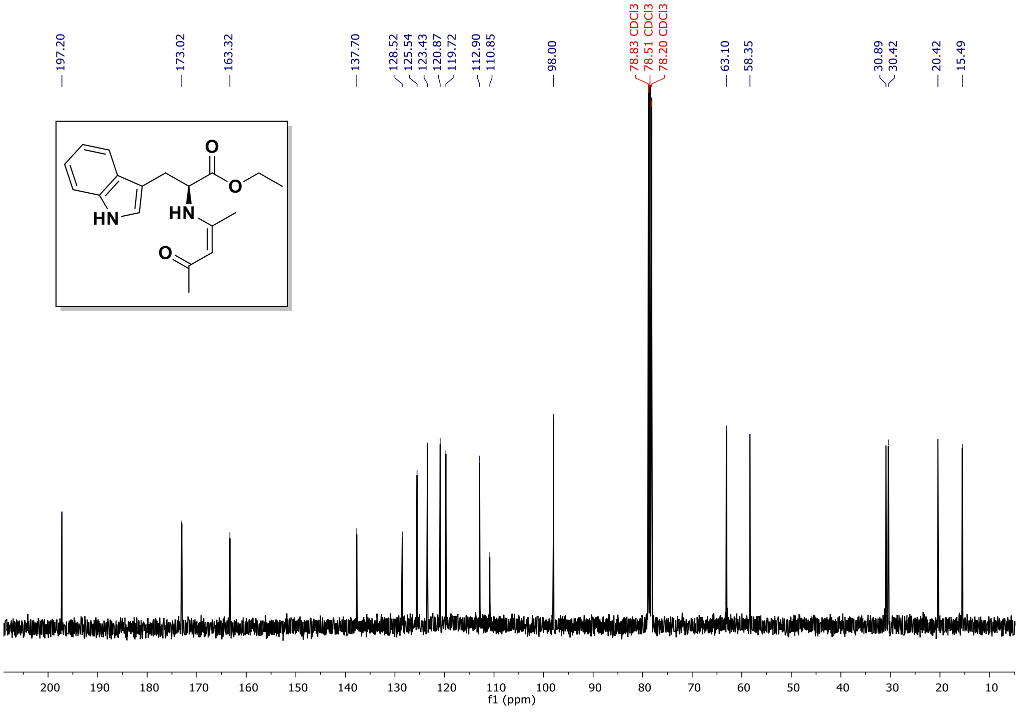

13C NMR spectrum of 2 in $CDCl_3$.

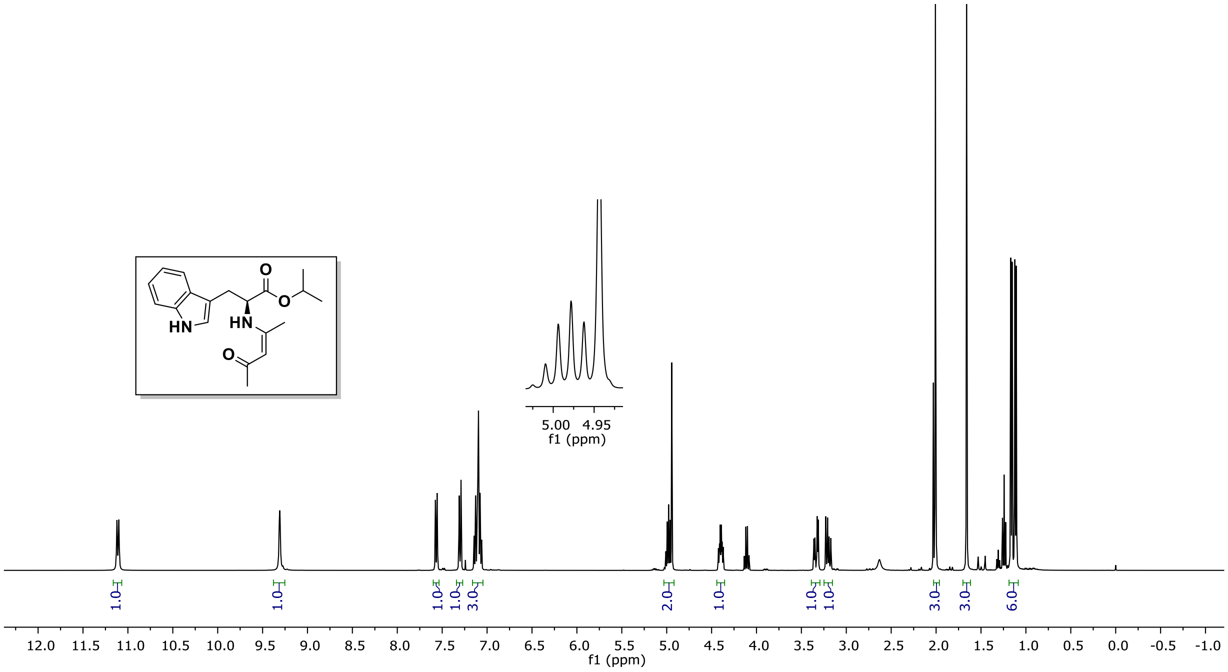

1H NMR spectrum of 3 in $CDCl_3$.

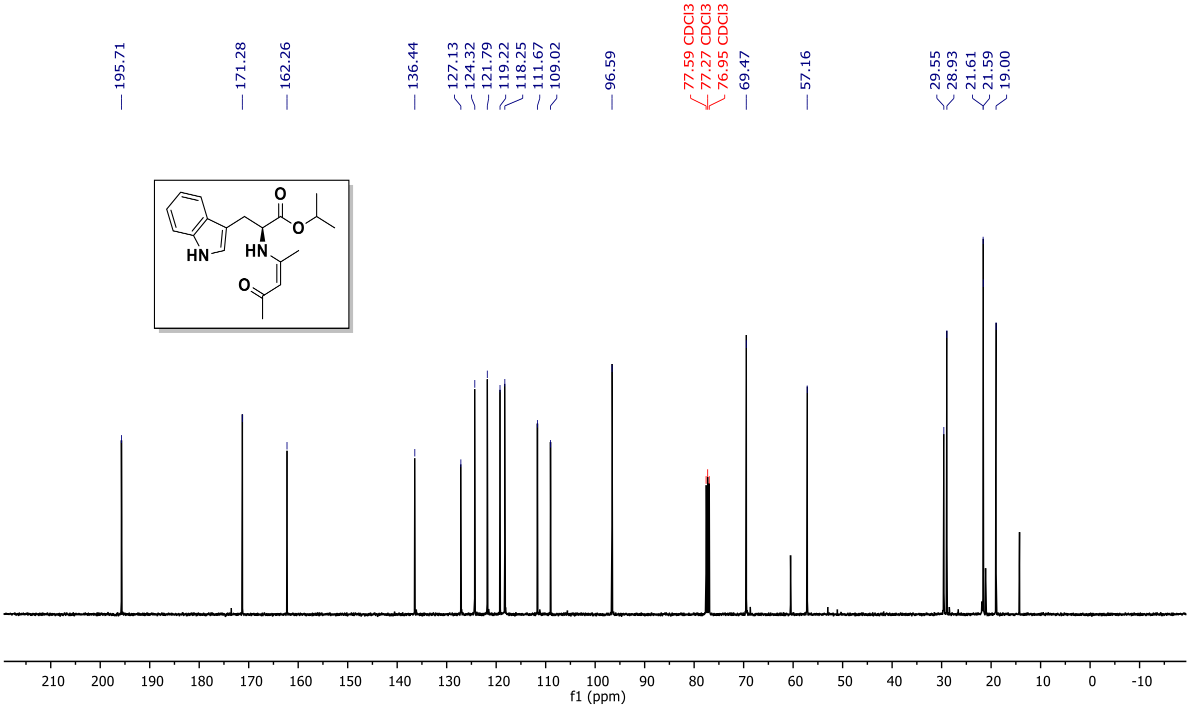

13C NMR spectrum of 3 in $CDCl_3$.

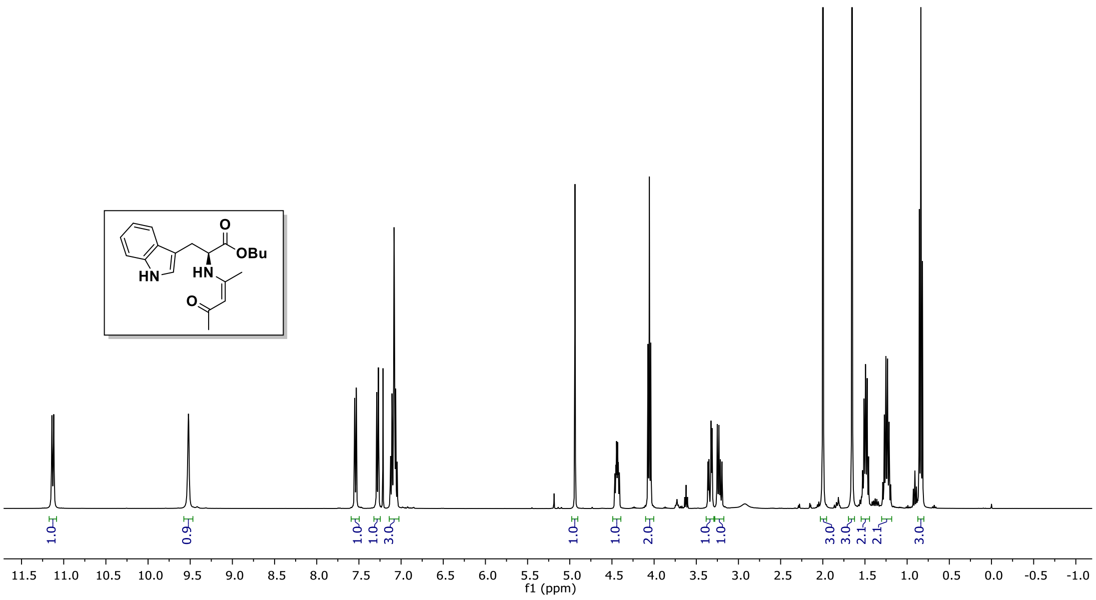

1H NMR spectrum of 4 in CDCl3.

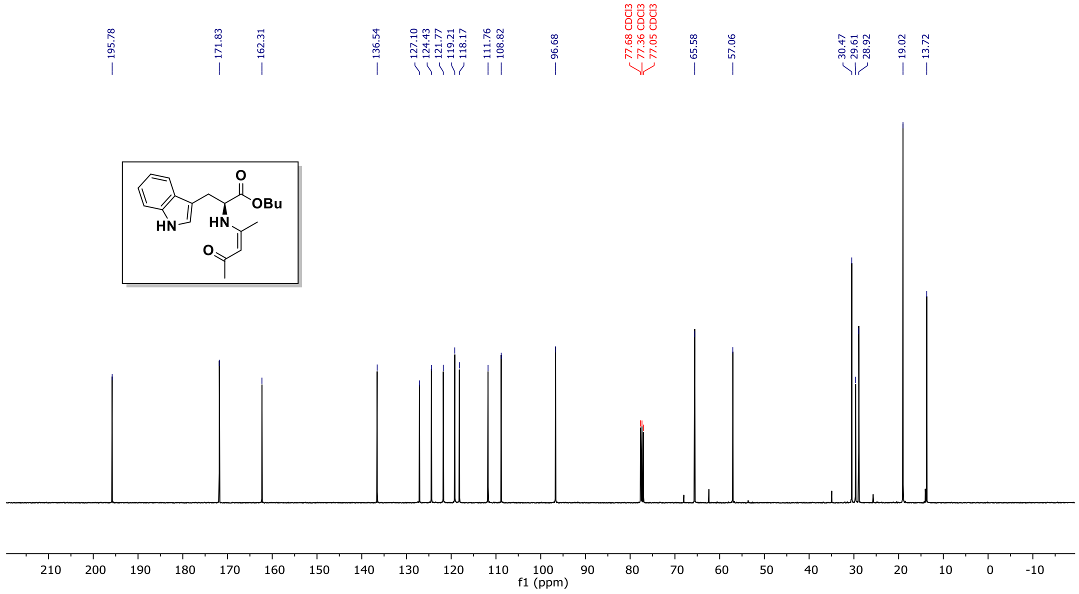

13C NMR spectrum of 4 in CDCl3.

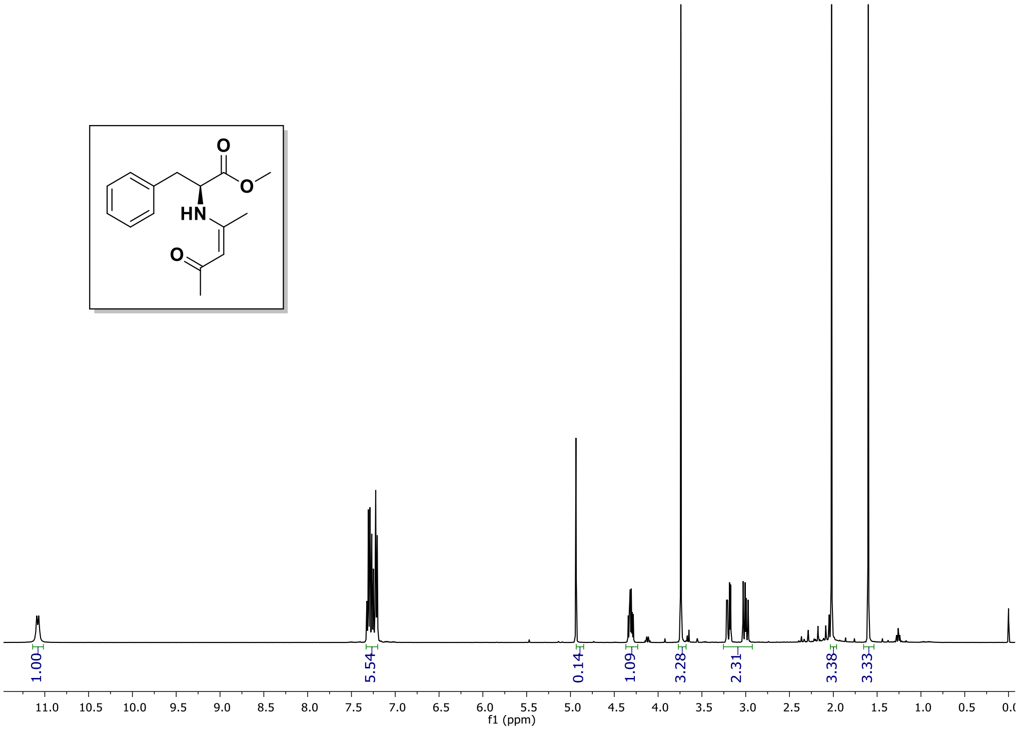

1H NMR spectrum of 5 in CDCl3.

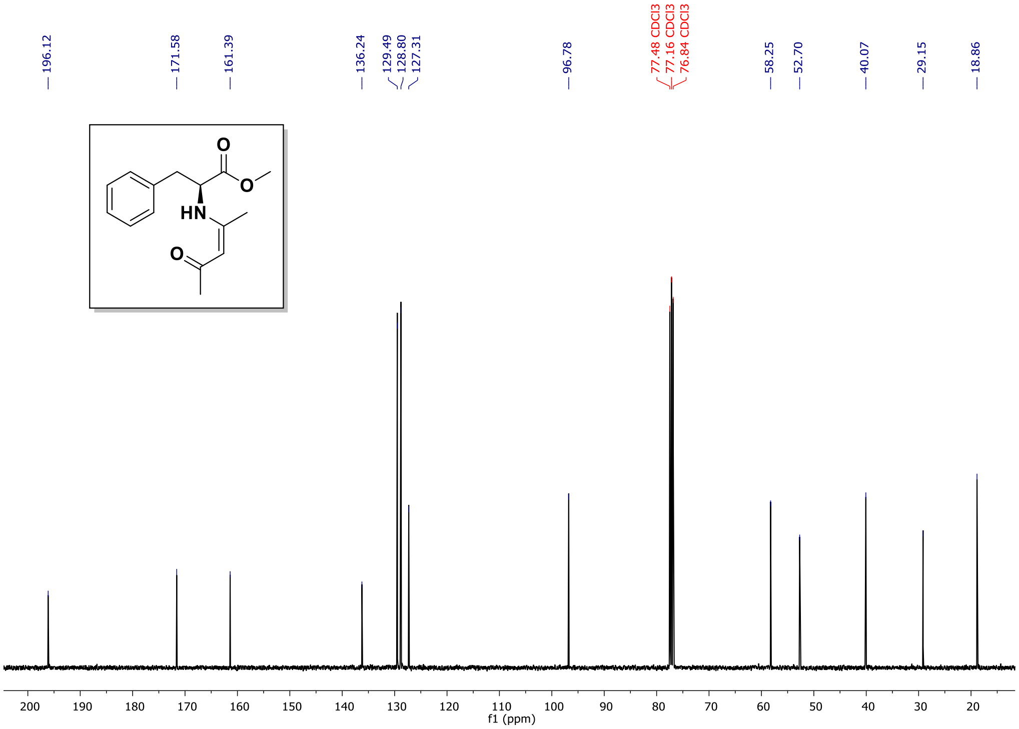

13C NMR spectrum of 5 in CDCl3.

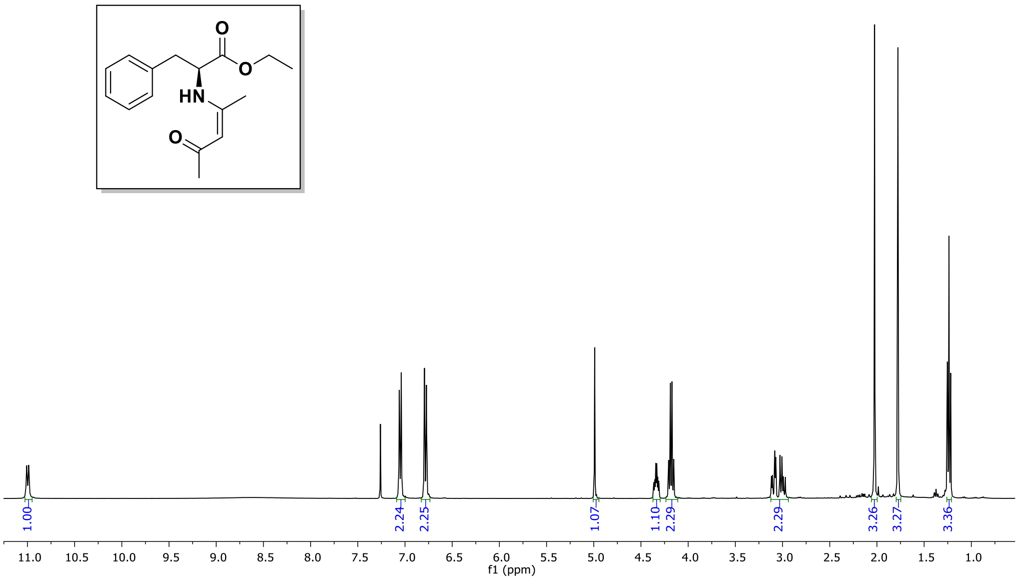

1H NMR spectrum of 6 in CDCl3.

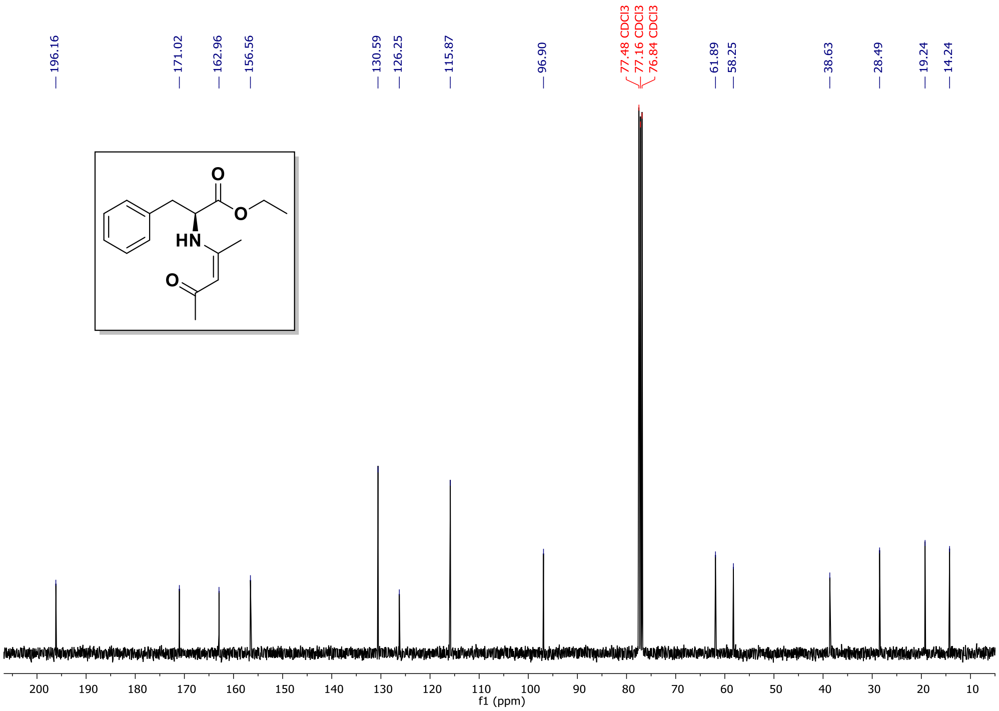

13C NMR spectrum of 6 in CDCl3.

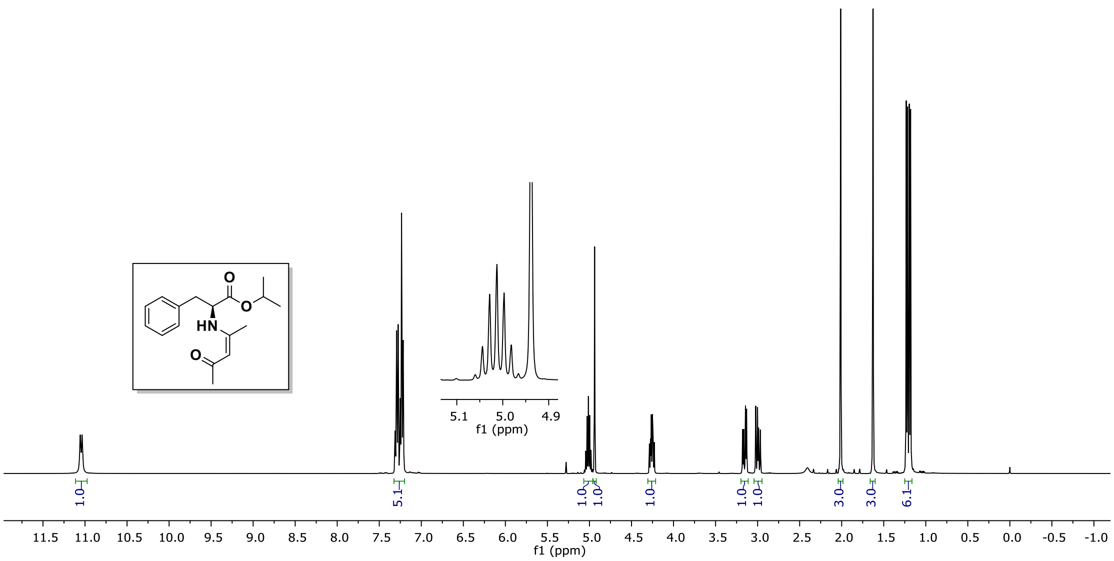

1H NMR spectrum of 7 in CDCl3.

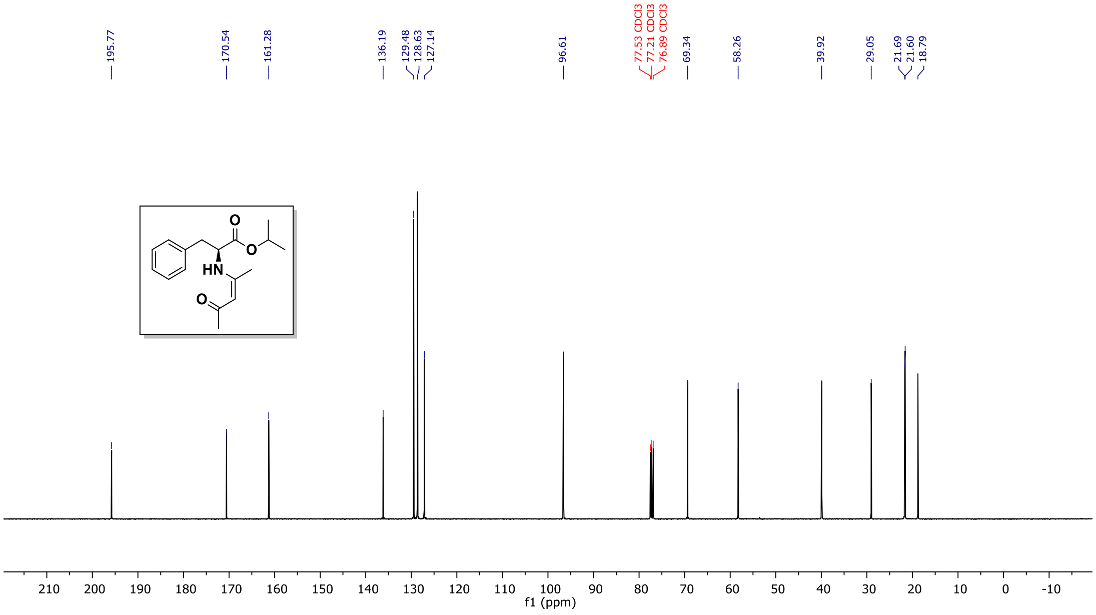

13C NMR spectrum of 7 in CDCl3.

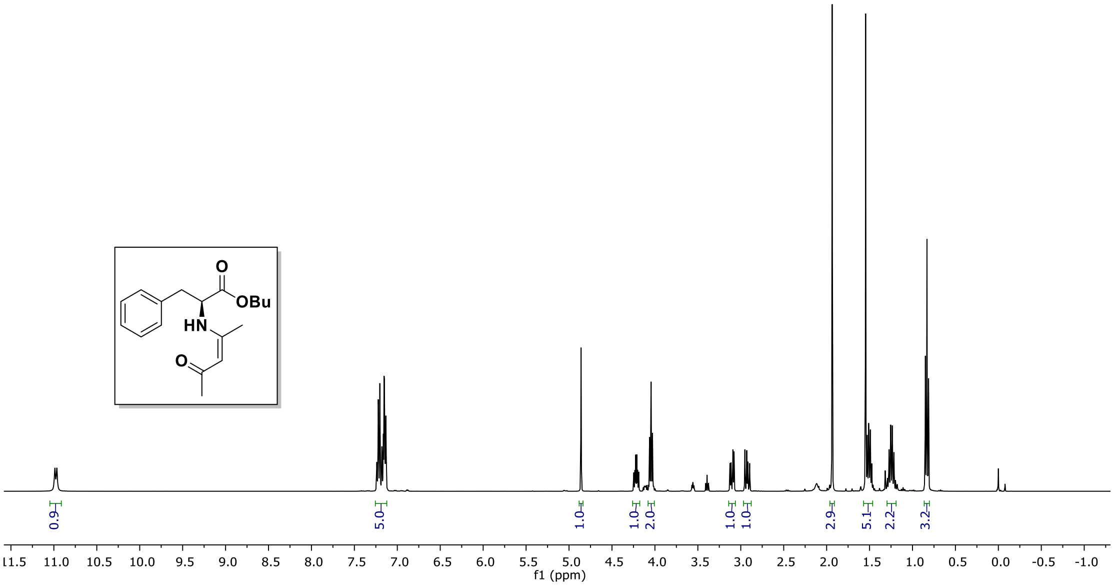

1H NMR spectrum of 8 in CDCl3.

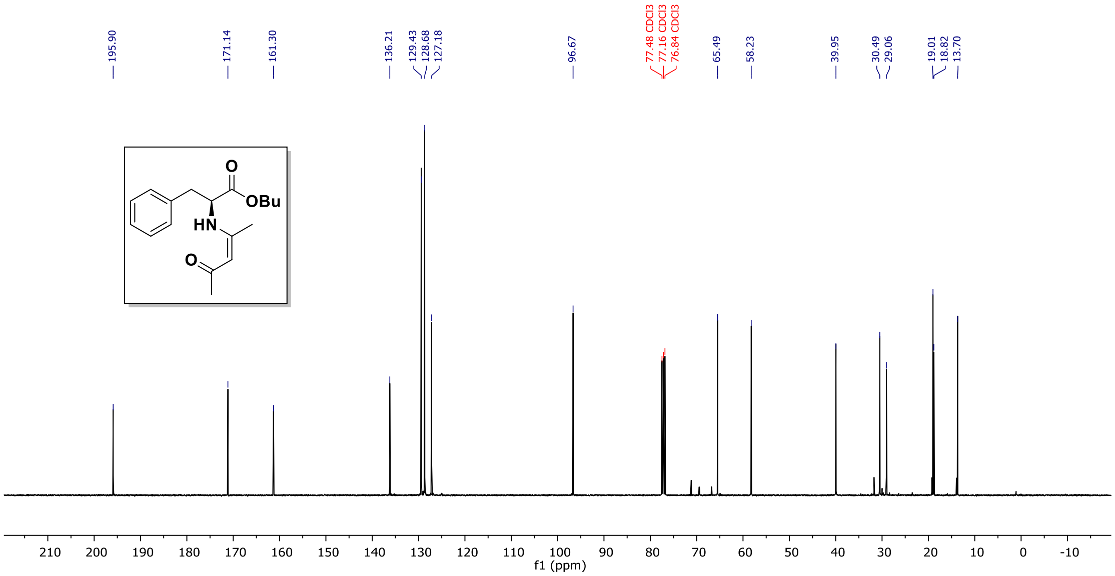

13C NMR spectrum of 8 in CDCl3.

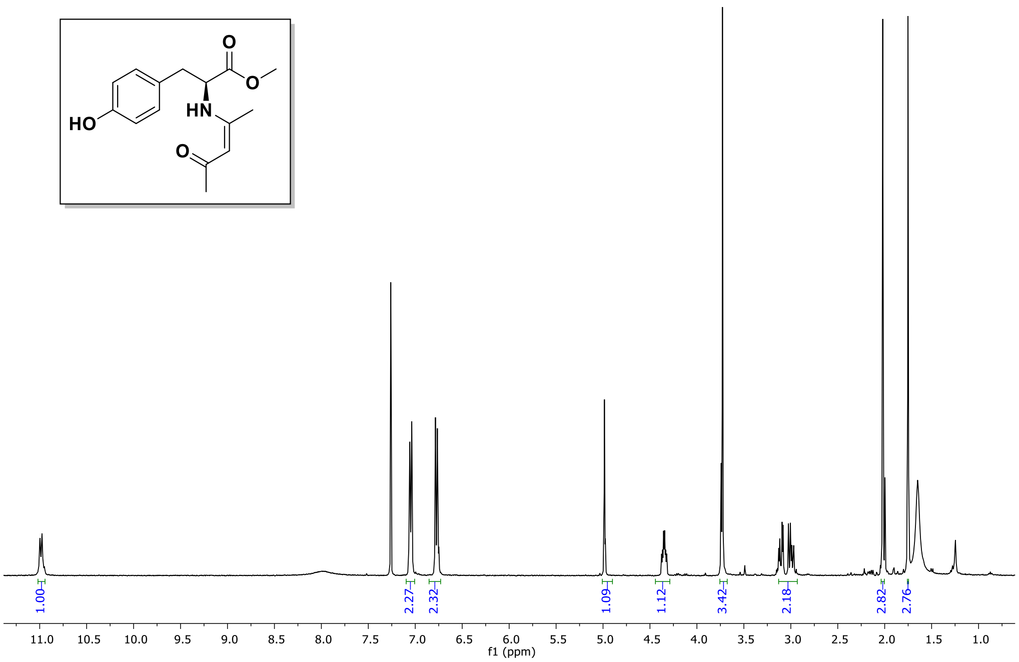

1H NMR spectrum of 9 in CDCl3.

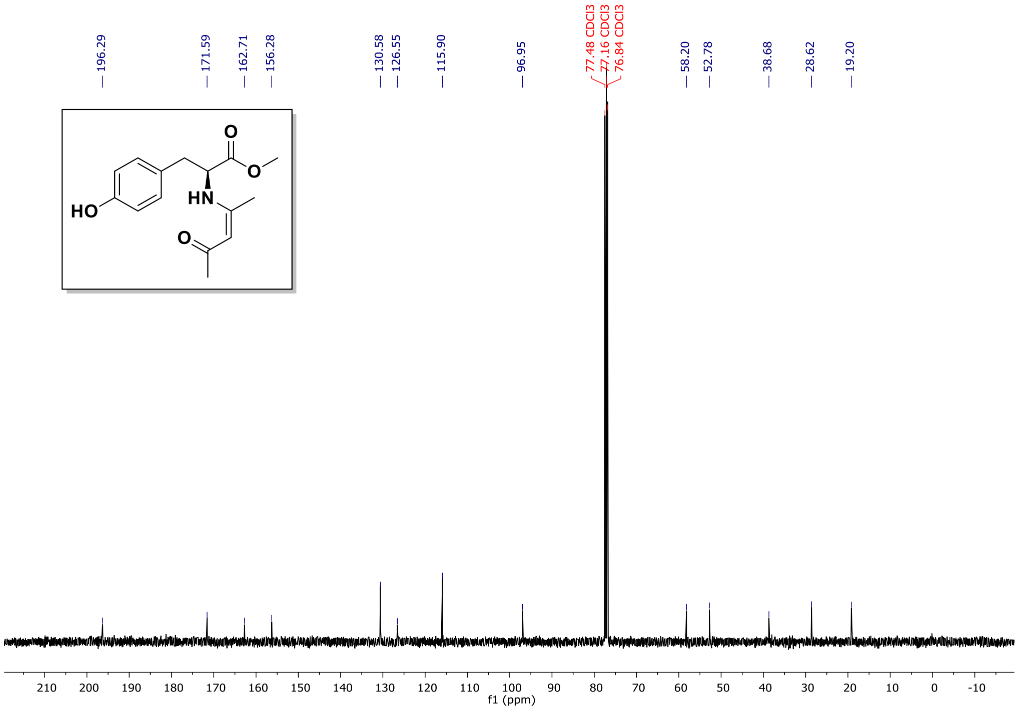

13C NMR spectrum of 9 in CDCl3.

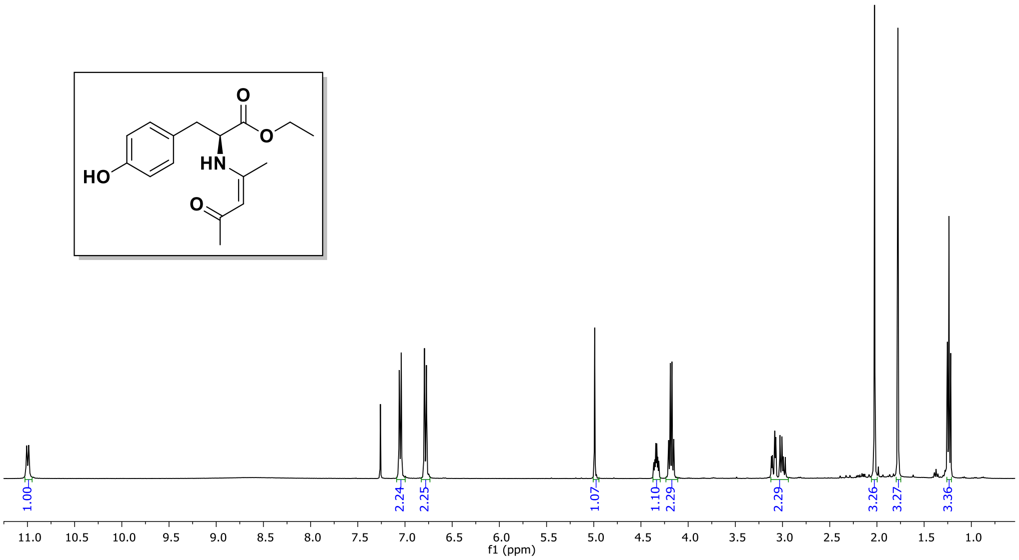

1H NMR spectrum of 10 in $CDCl_3$.

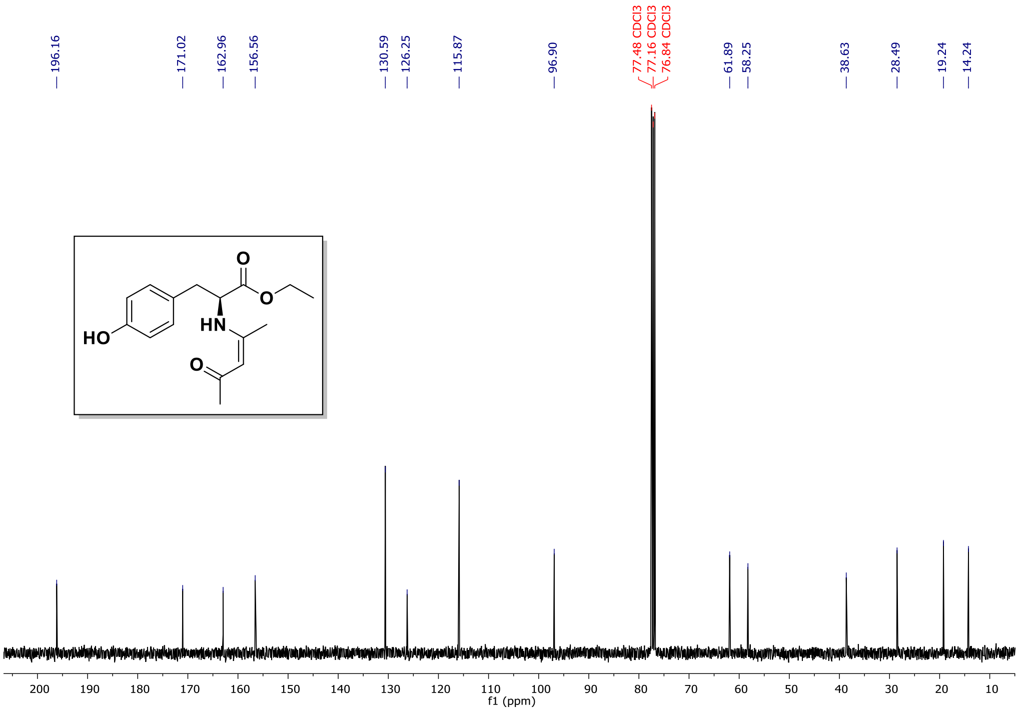

13C NMR spectrum of 10 in $CDCl_3$.

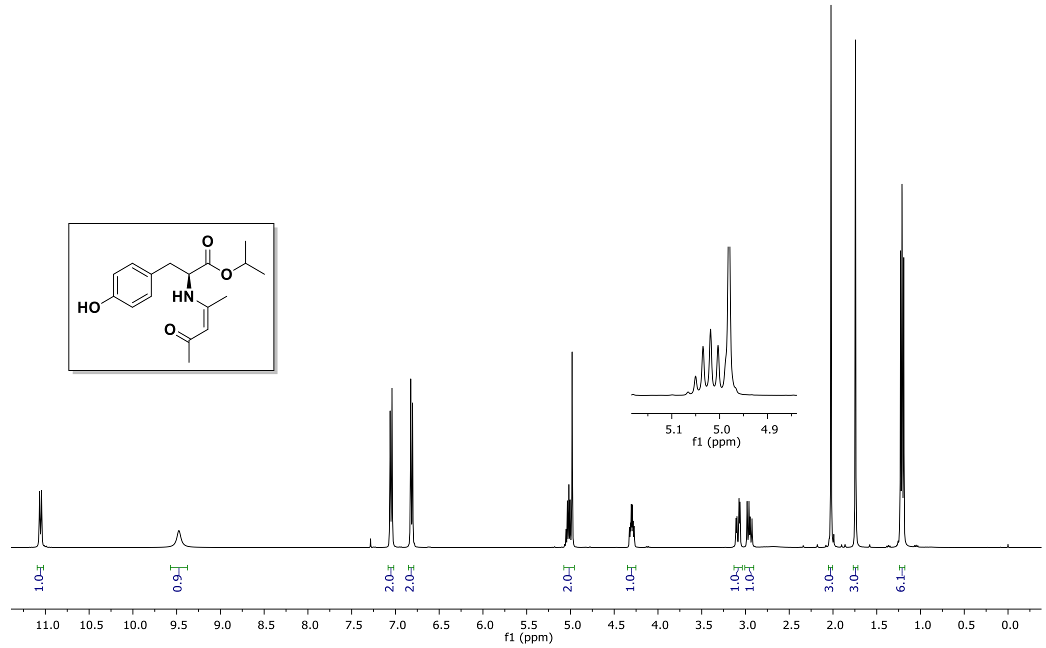

1H NMR spectrum of 11 in CDCl3.

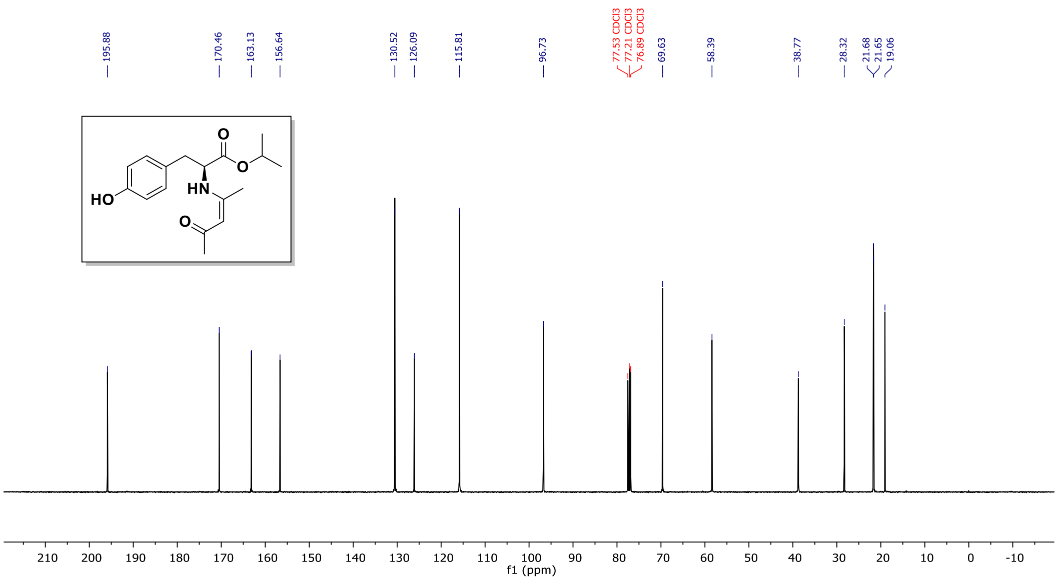

13C NMR spectrum of 11 in CDCl3.

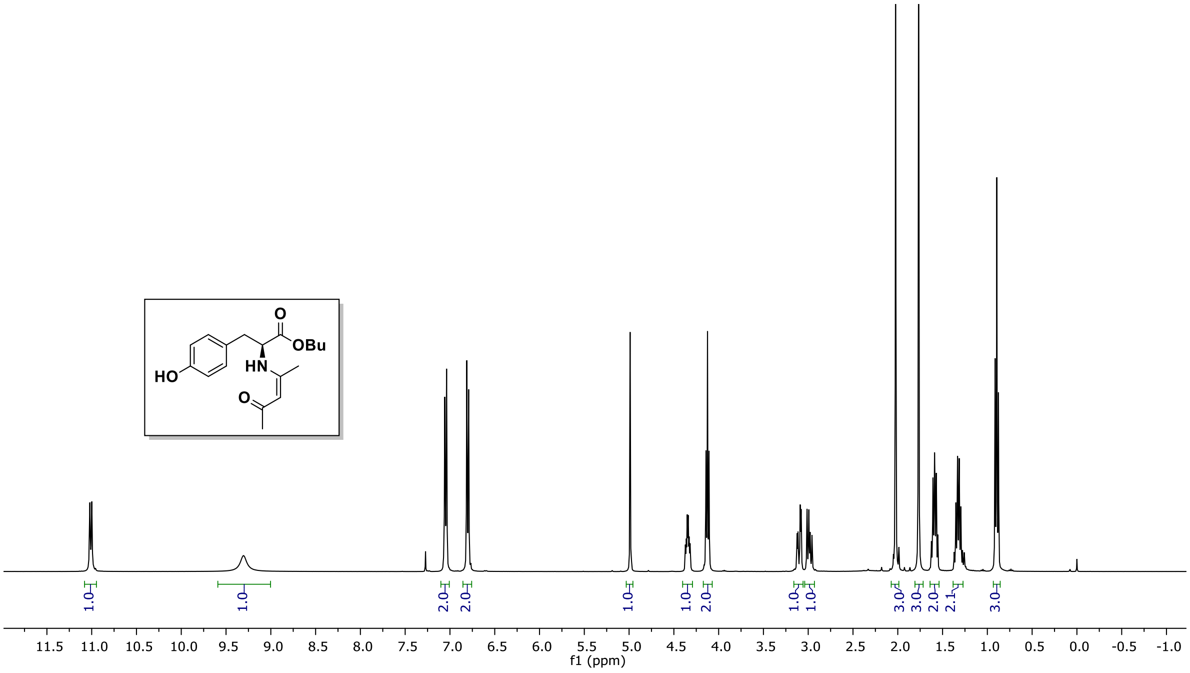

1H NMR spectrum of 12 in $CDCl_3$.

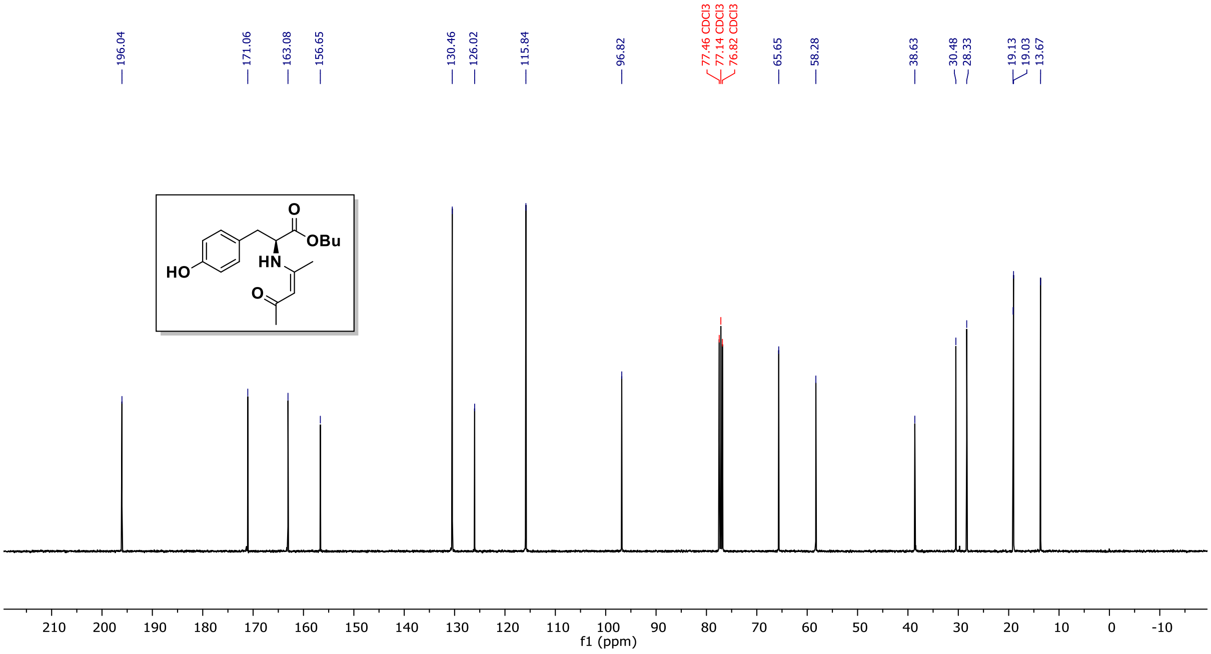

13C NMR spectrum of 12 in $CDCl_3$.

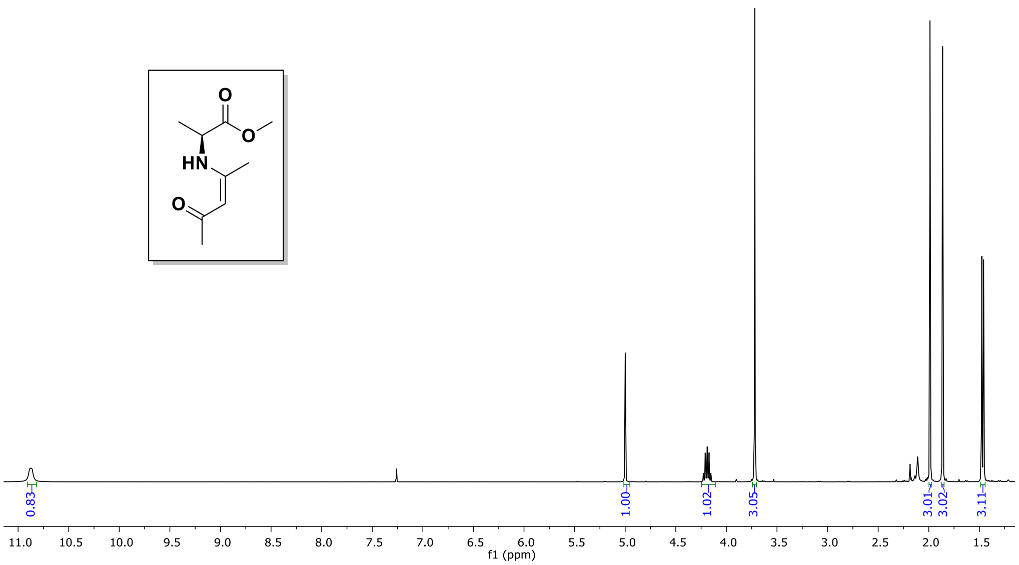

1H NMR spectrum of 13 in $CDCl_3$.

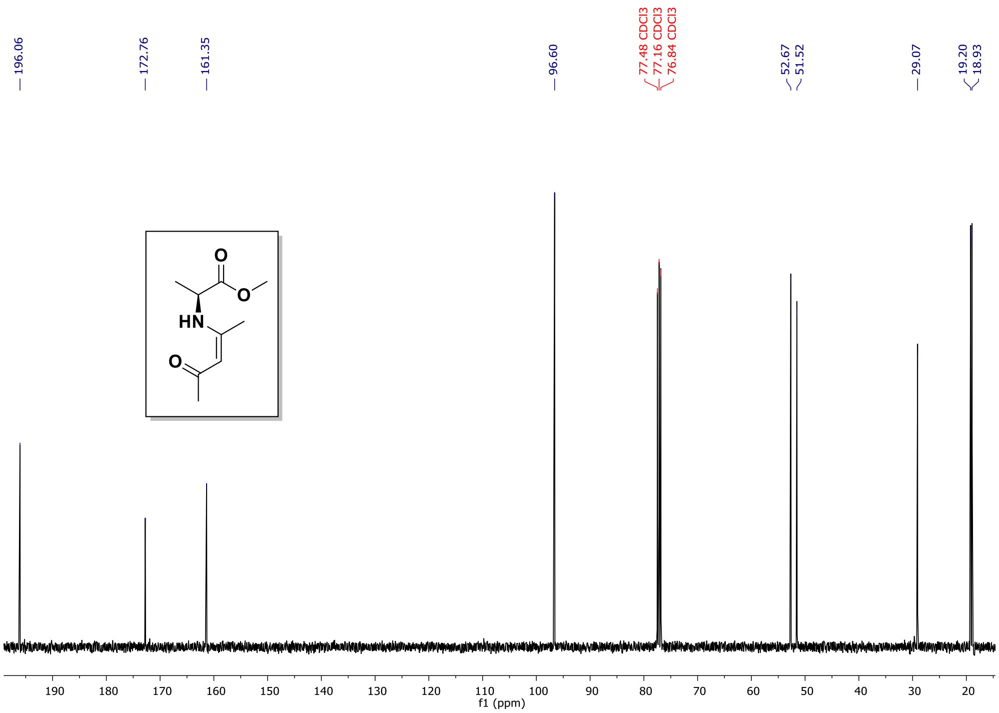

13C NMR spectrum of 13 in $CDCl_3$.

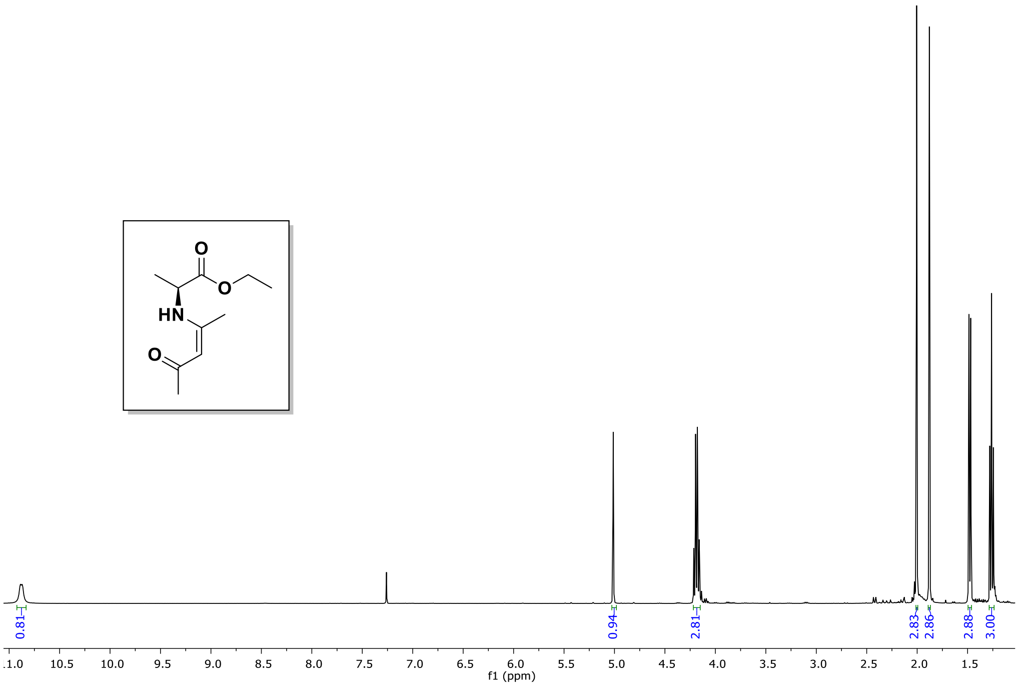

1H NMR spectrum of 14 in CDCl3.

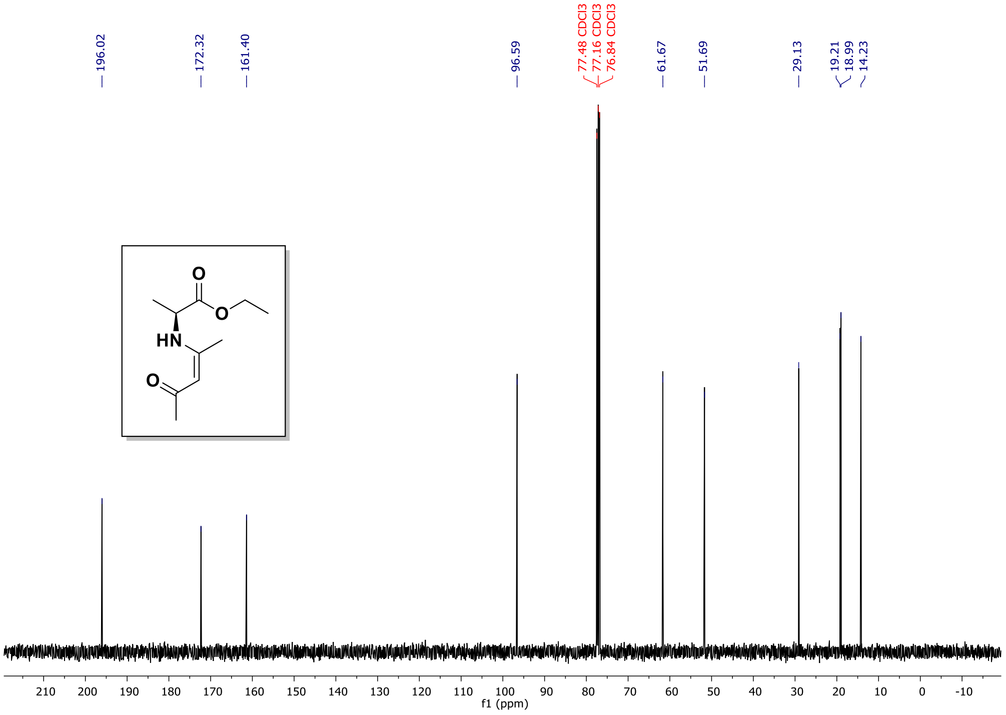

13C NMR spectrum of 14 in CDCl3.

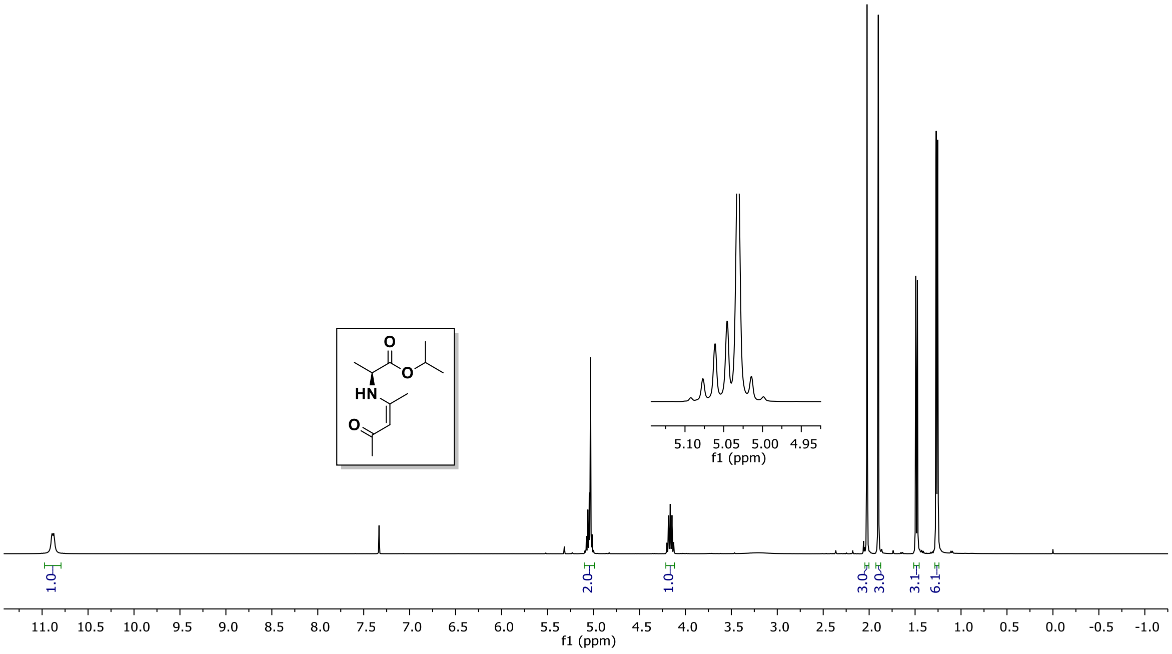

1H NMR spectrum of 15 in CDCl3.

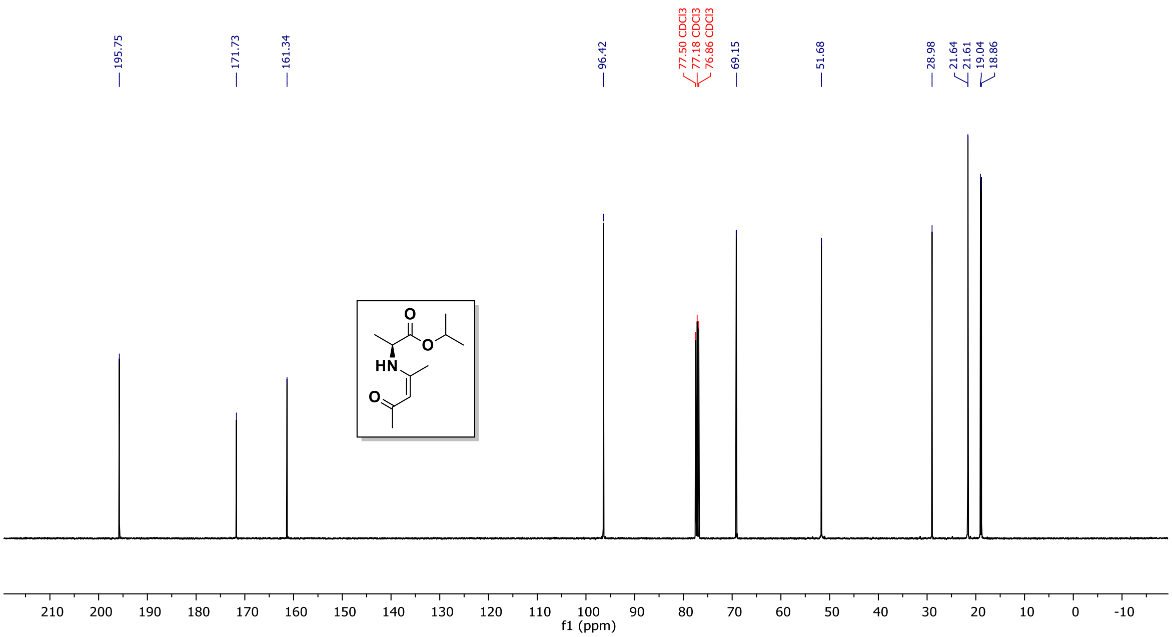

13C NMR spectrum of 15 in CDCl3.

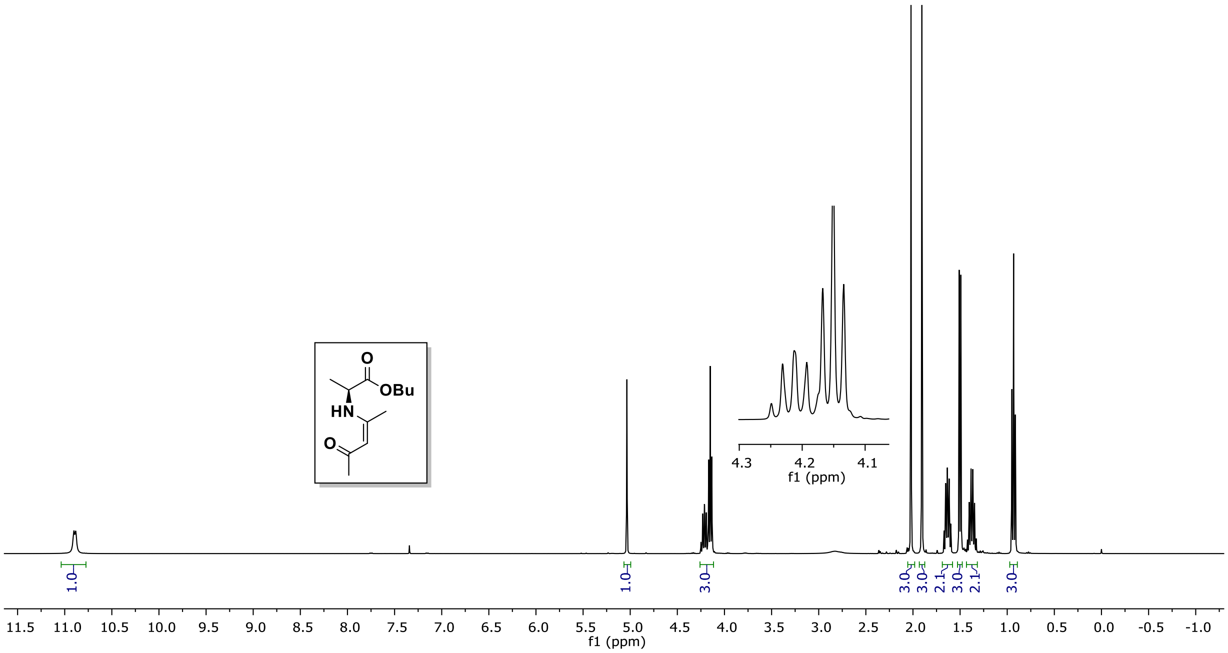

1H NMR spectrum of 16 in $CDCl_3$.

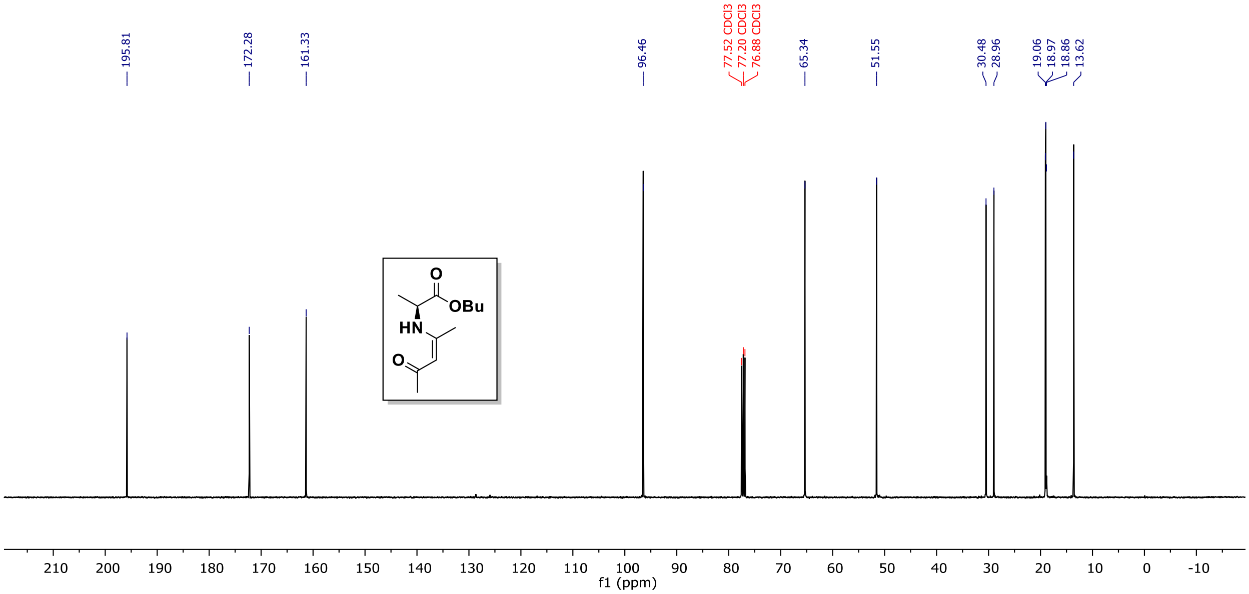

13C NMR spectrum of 16 in $CDCl_3$.

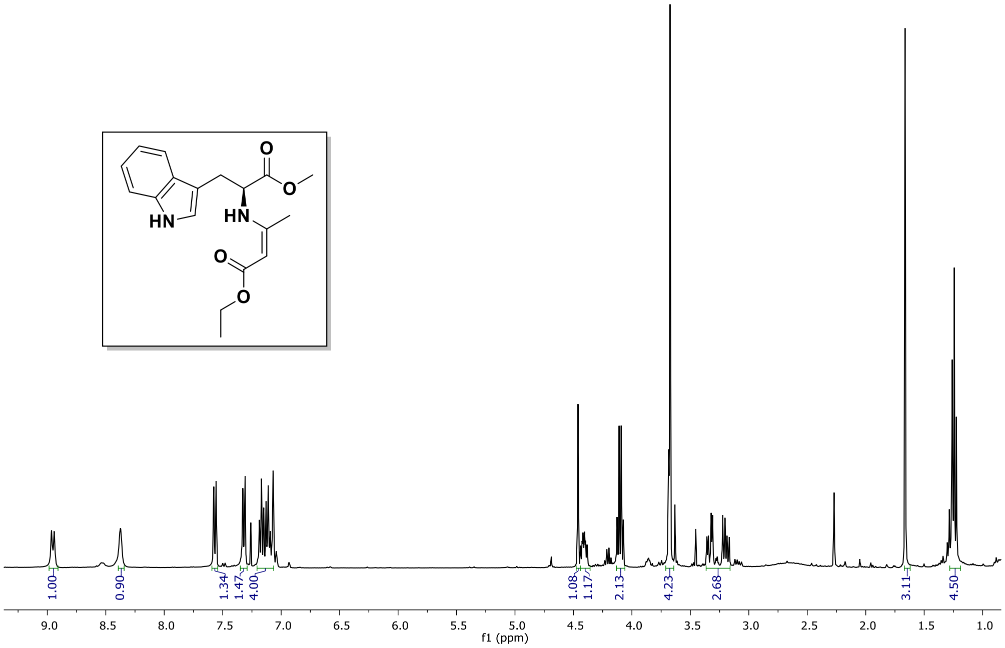

1H NMR spectrum of 17 in CDCl3.

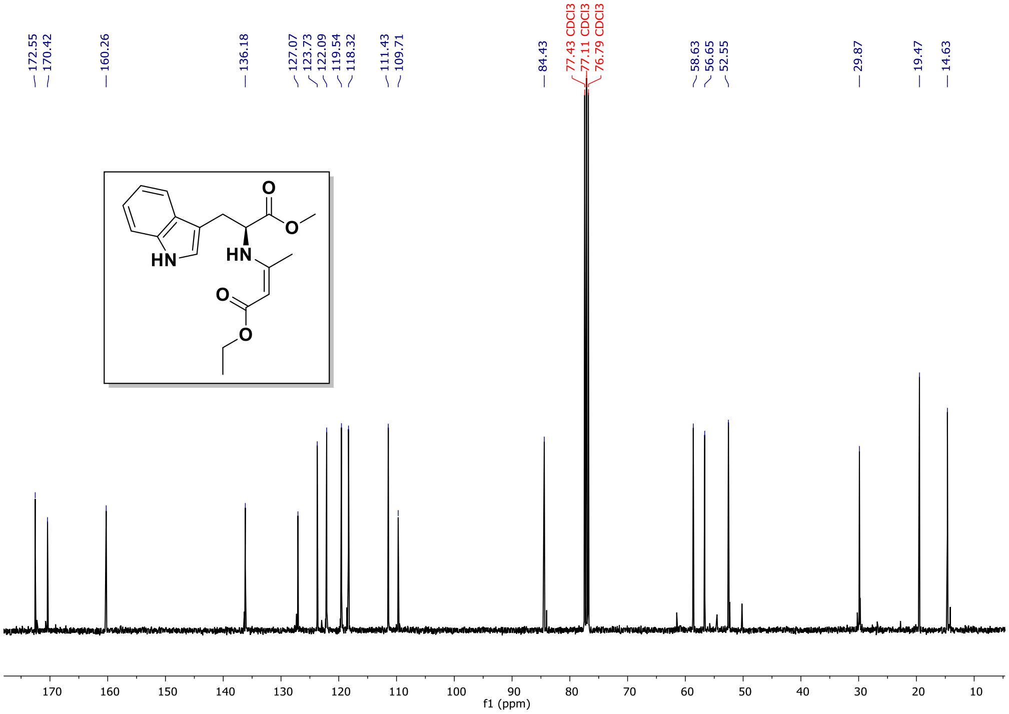

13C NMR spectrum of 17 in CDCl3.

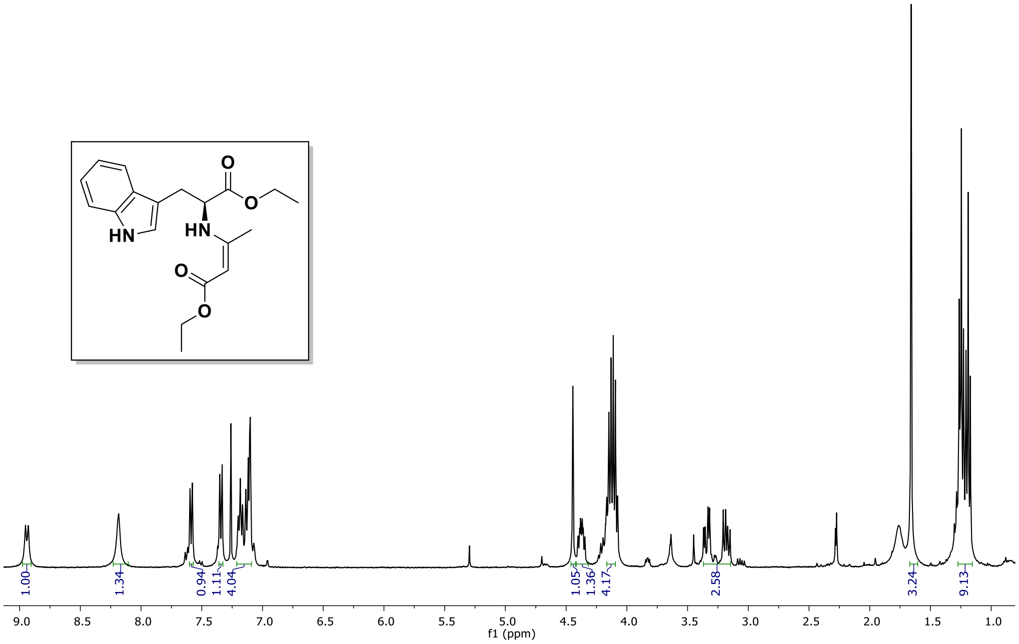

1H NMR spectrum of 18 in CDCl3.

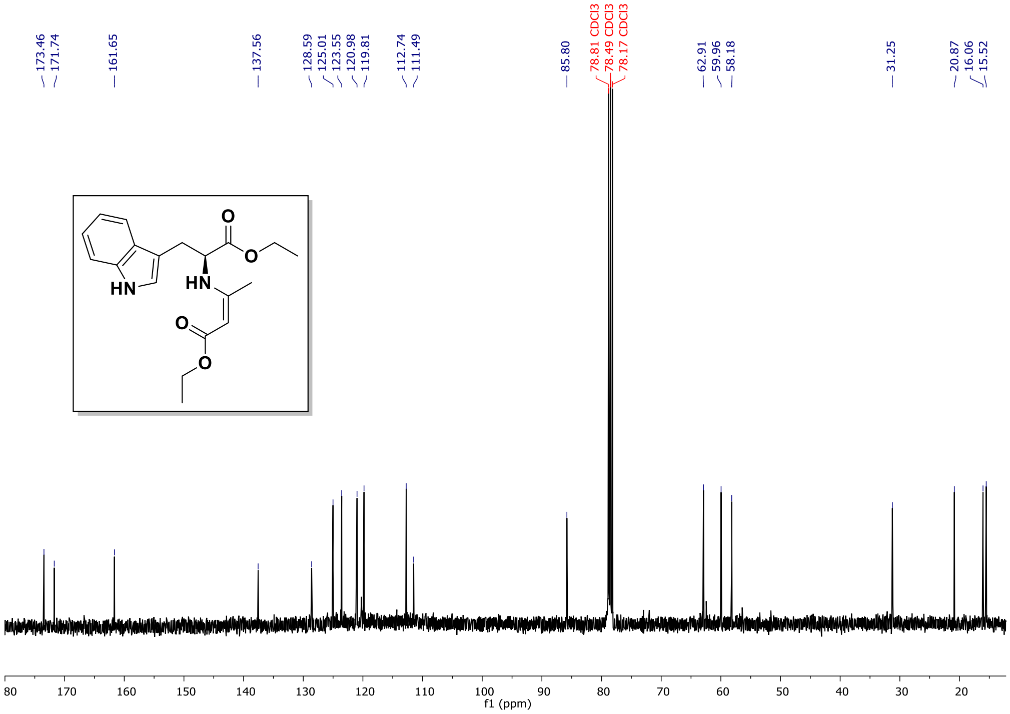

13C NMR spectrum of 18 in CDCl3.

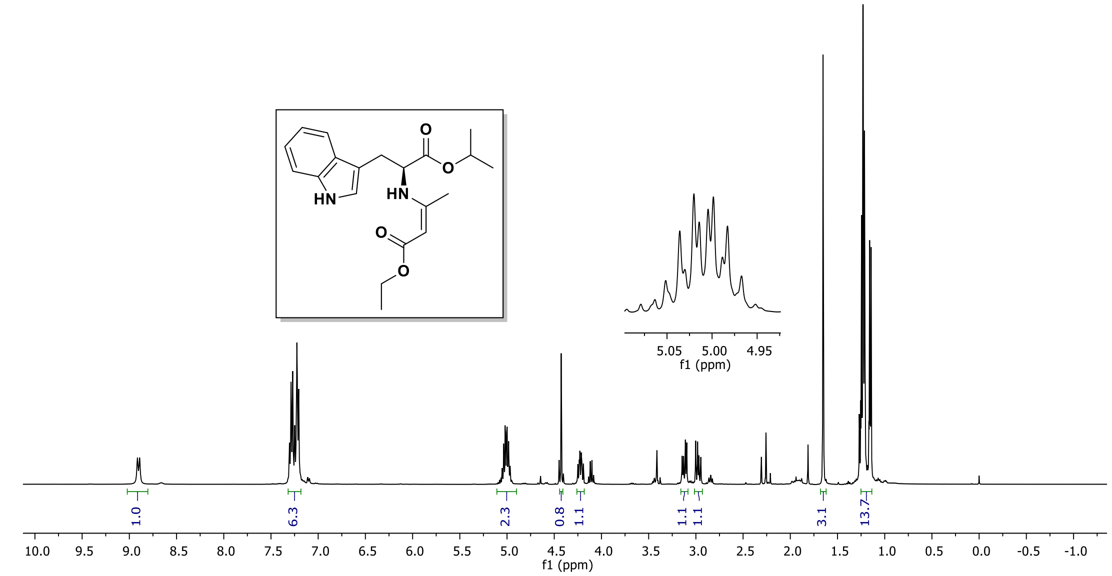

1H NMR spectrum of 19 in CDCl3.

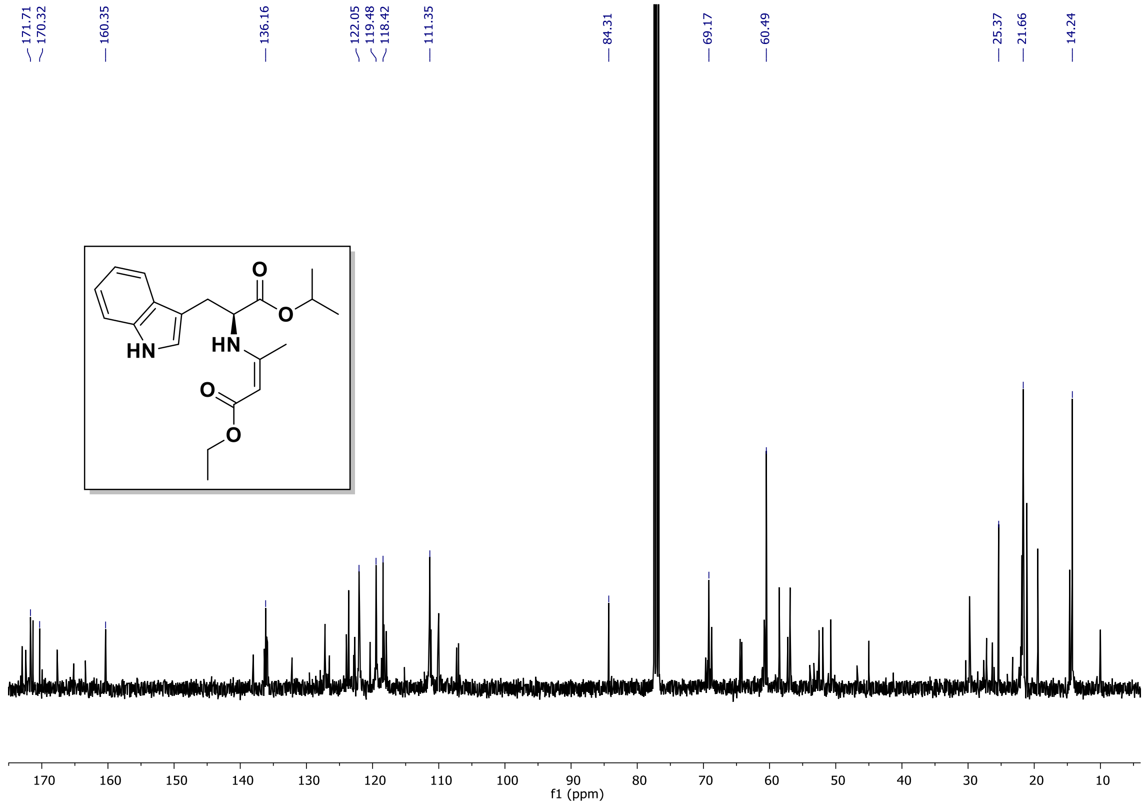

13C NMR spectrum of 19 in CDCl3.

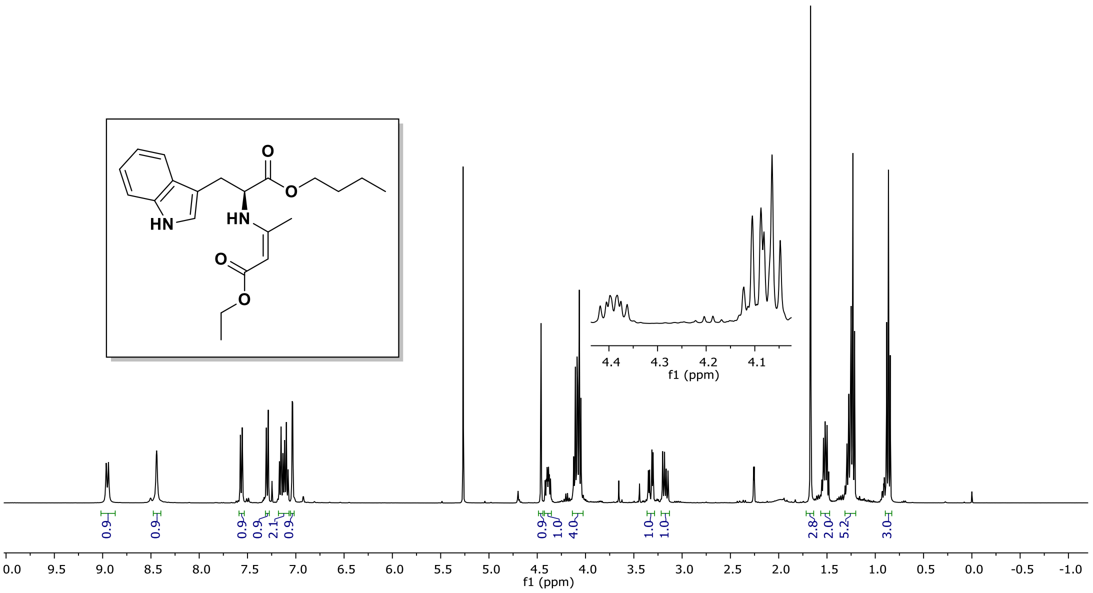

1H NMR spectrum of 20 in CDCl3.

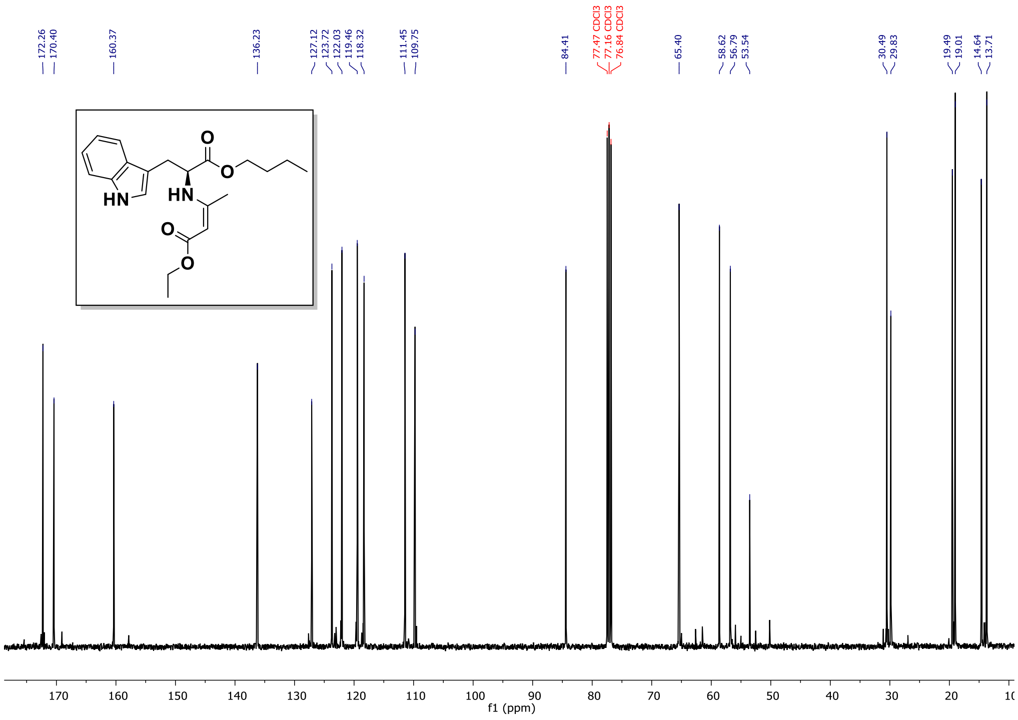

13C NMR spectrum of 20 in CDCl3.

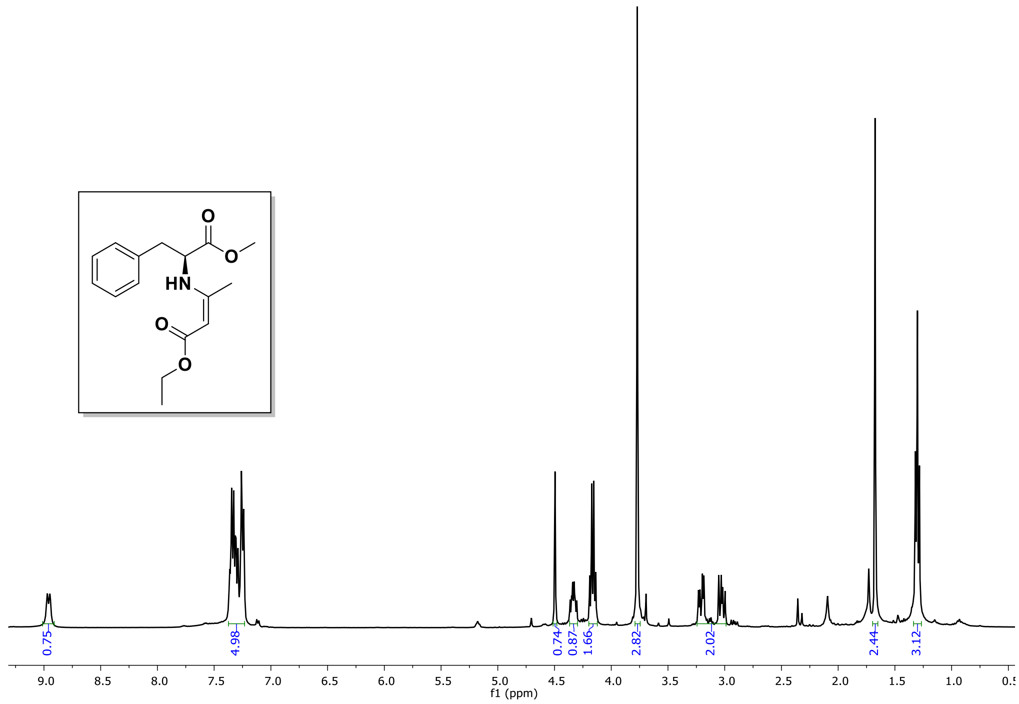

1H NMR spectrum of 21 in CDCl3.

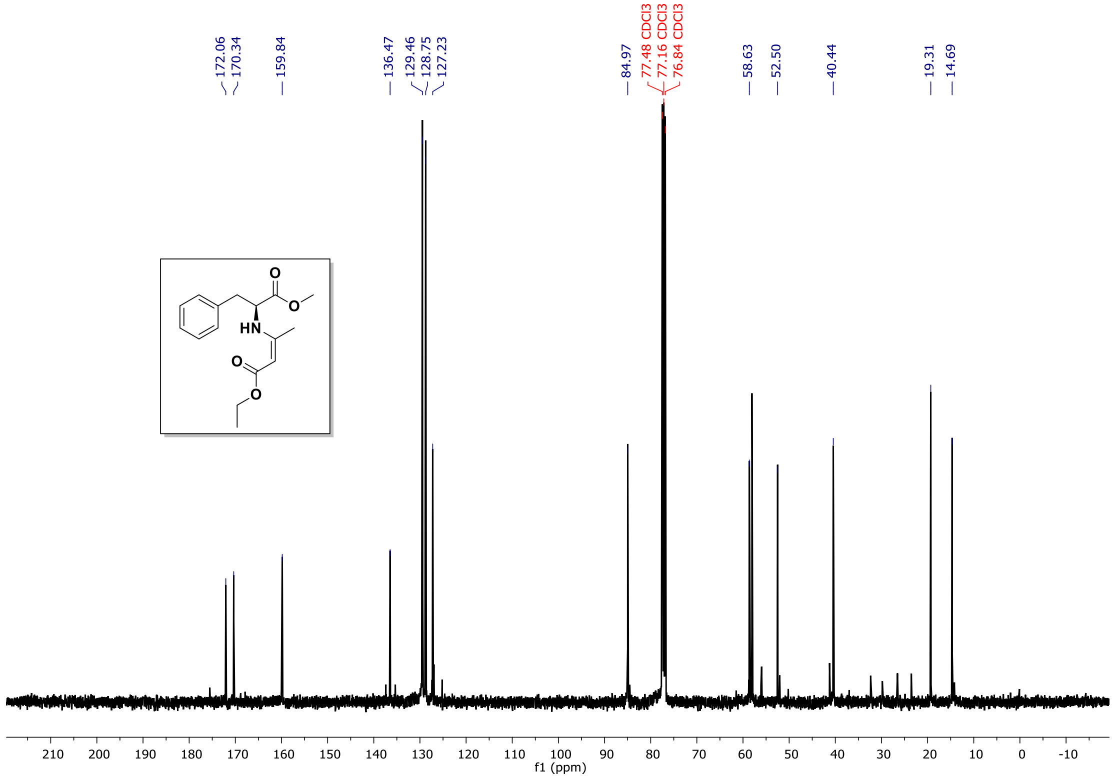

13C NMR spectrum of 21 in CDCl3.

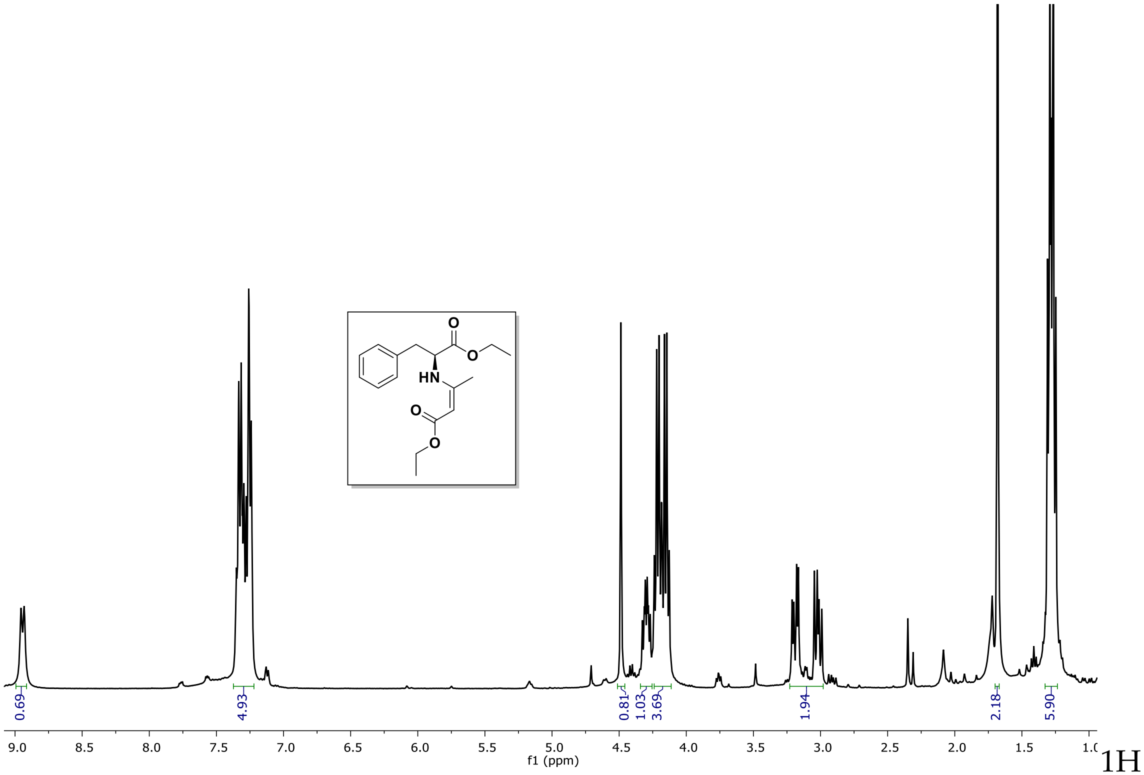

1H NMR spectrum of 22 in $CDCl_3$.

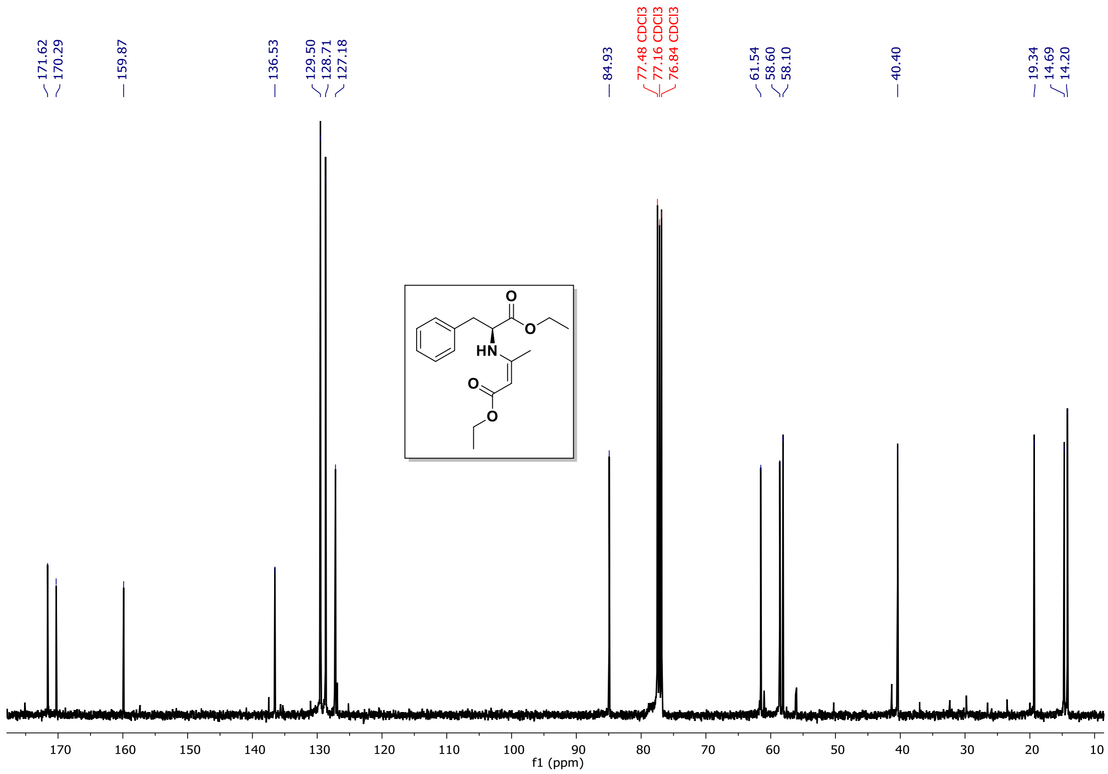

13C NMR spectrum of 22 in $CDCl_3$.

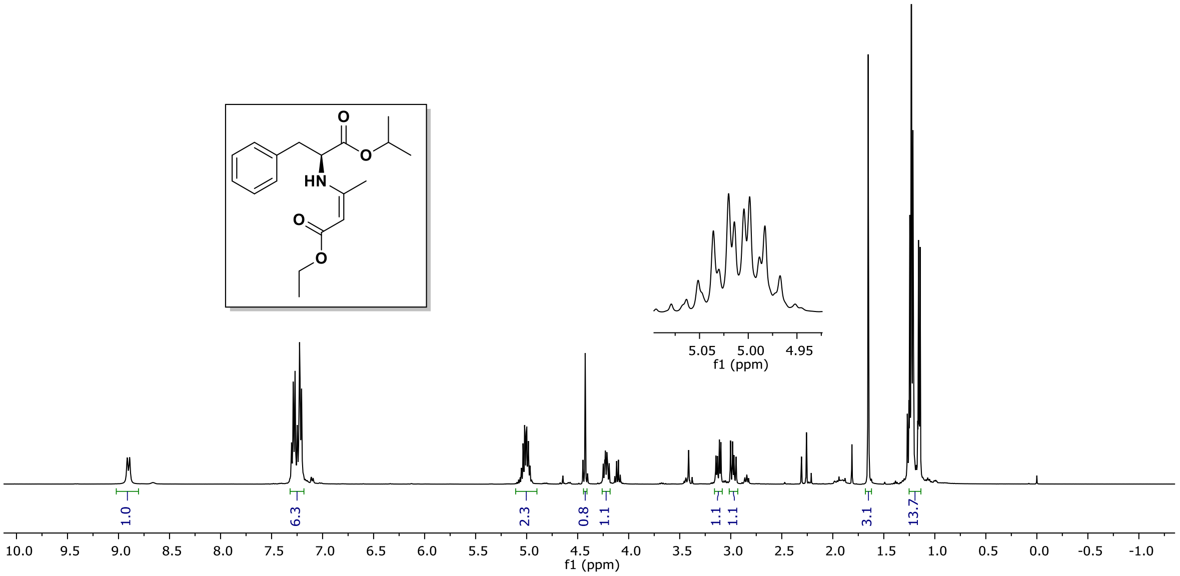

1H NMR spectrum of 23 in CDCl3.

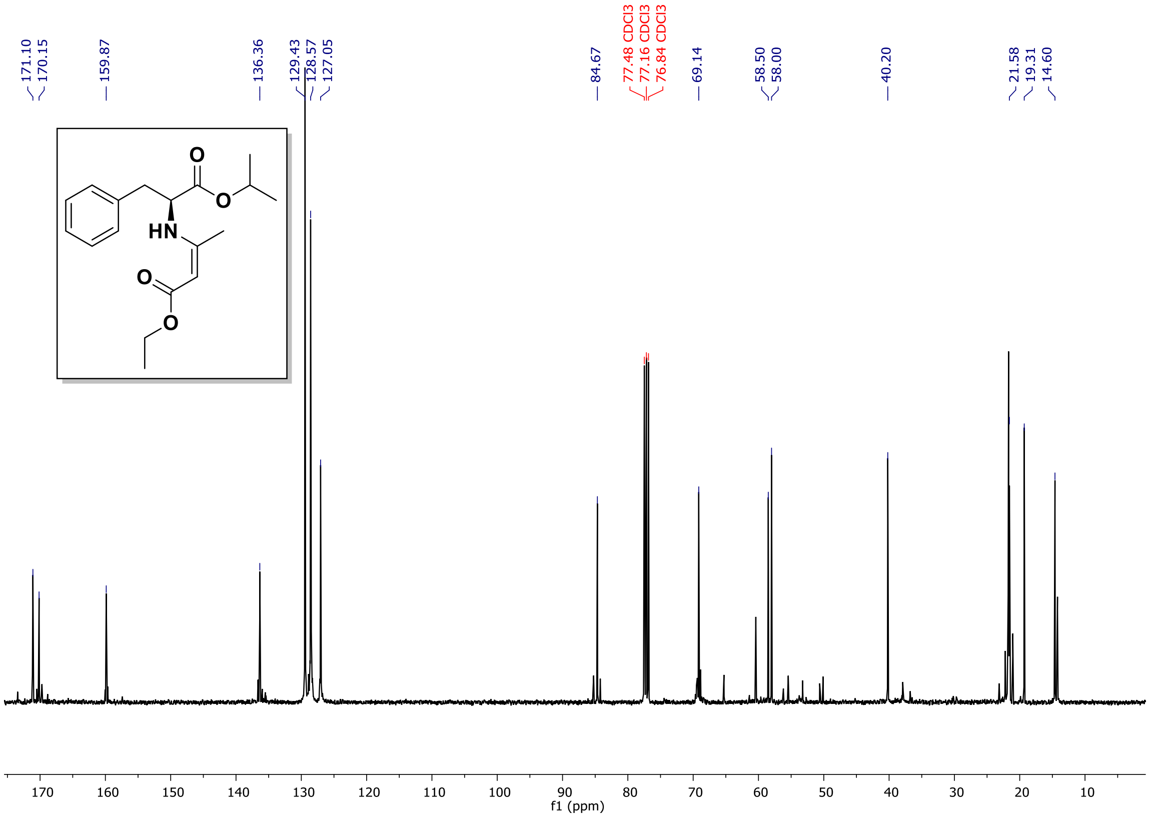

13C NMR spectrum of 23 in CDCl3.

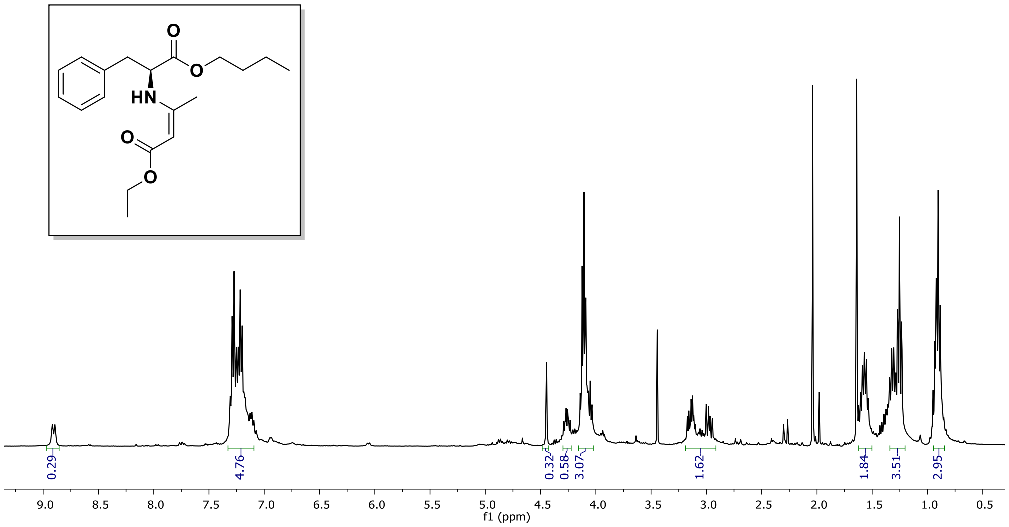

1H NMR spectrum of 24 in $CDCl_3$.

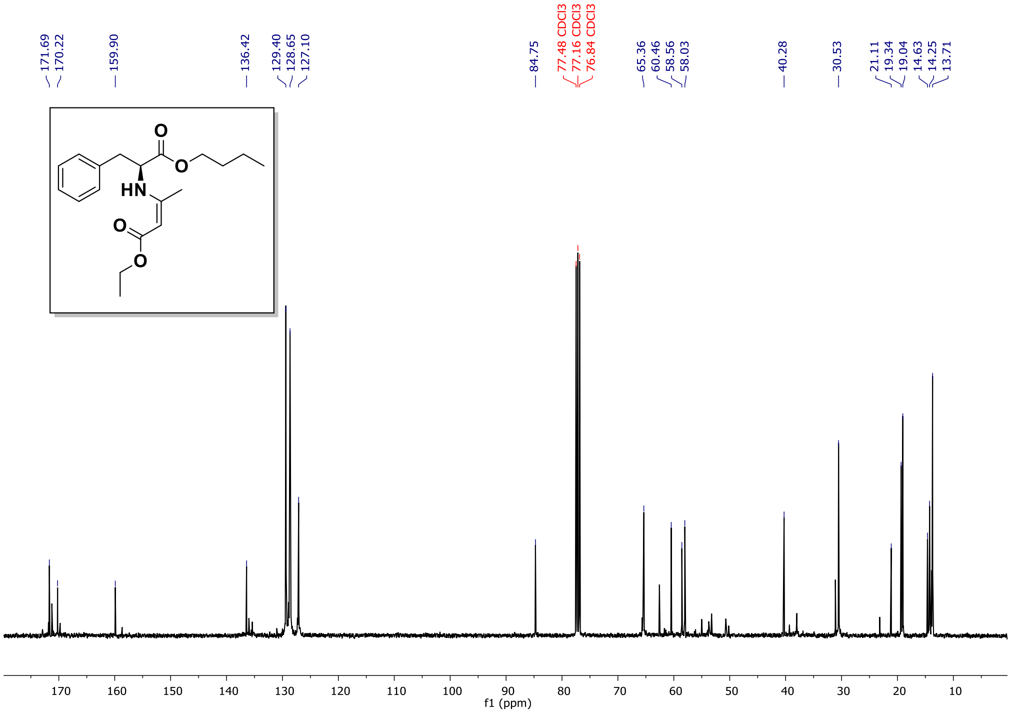

13C NMR spectrum of 24 in $CDCl_3$.

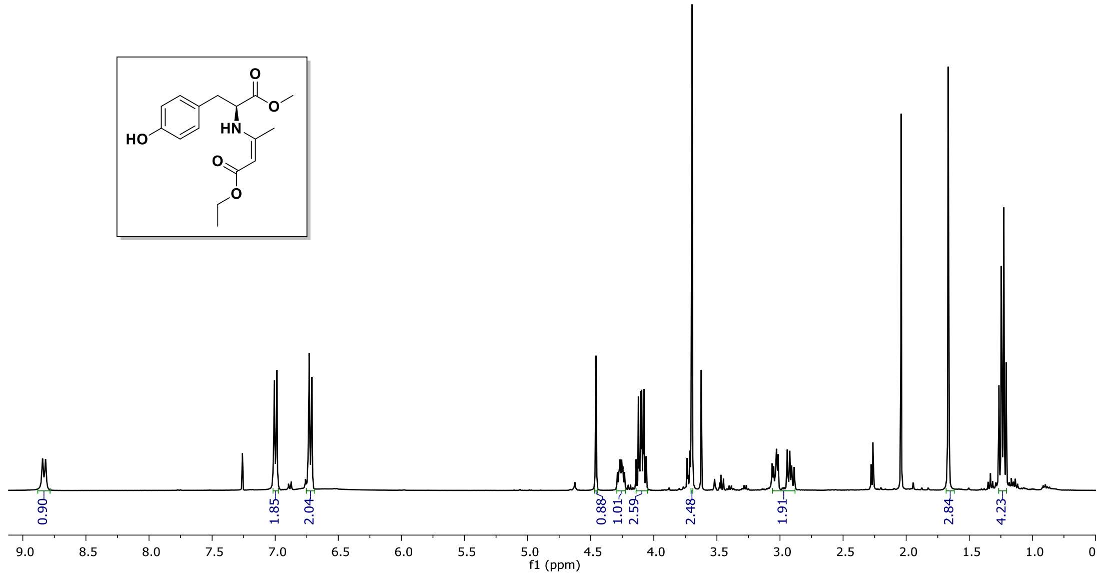

1H NMR spectrum of 25 in $CDCl_3$.

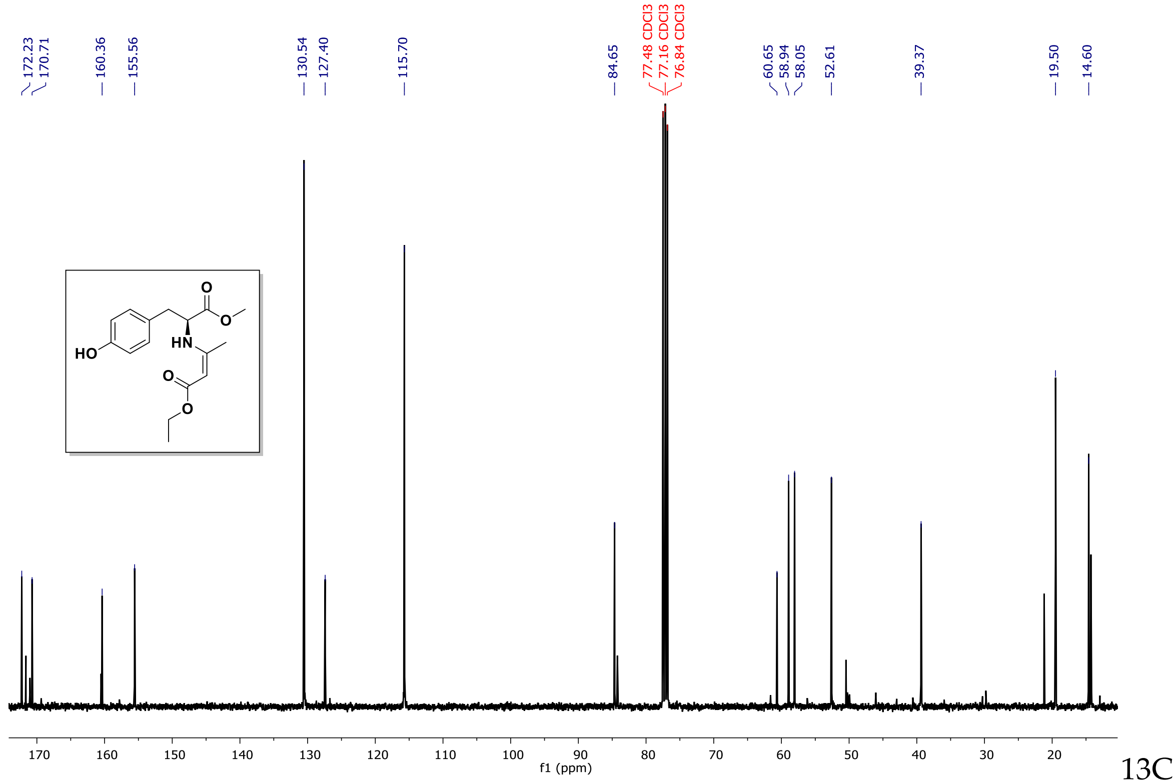

13C NMR spectrum of 25 in $CDCl_3$.

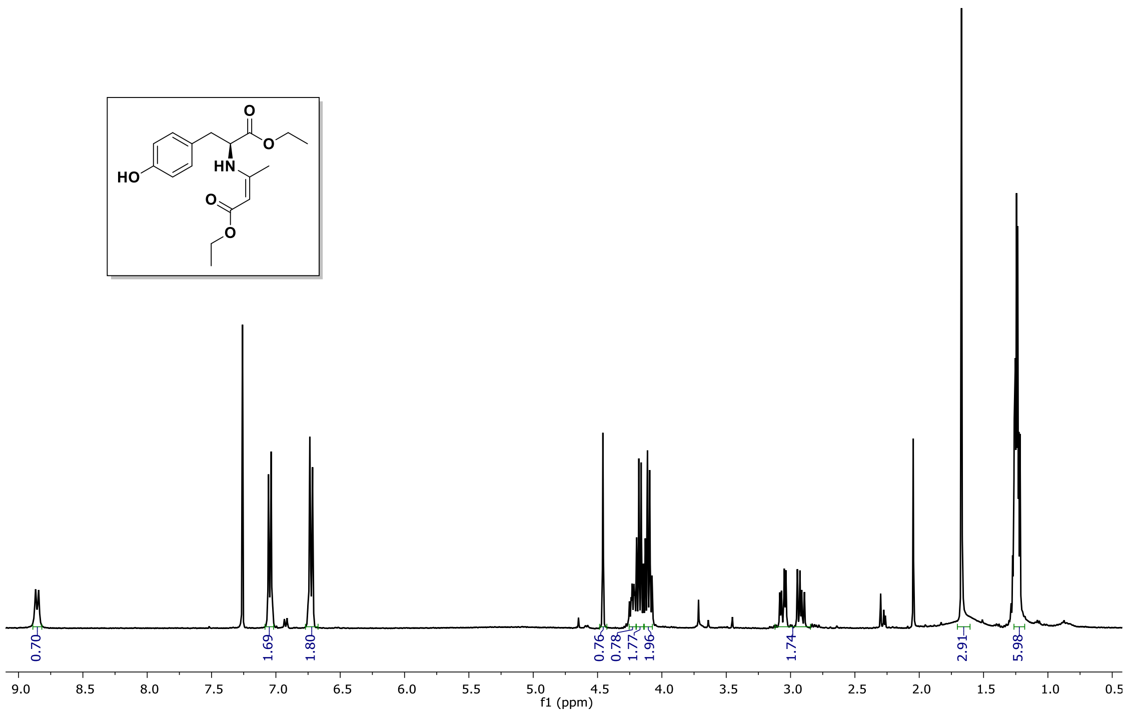

1H NMR spectrum of 26 in CDCl3.

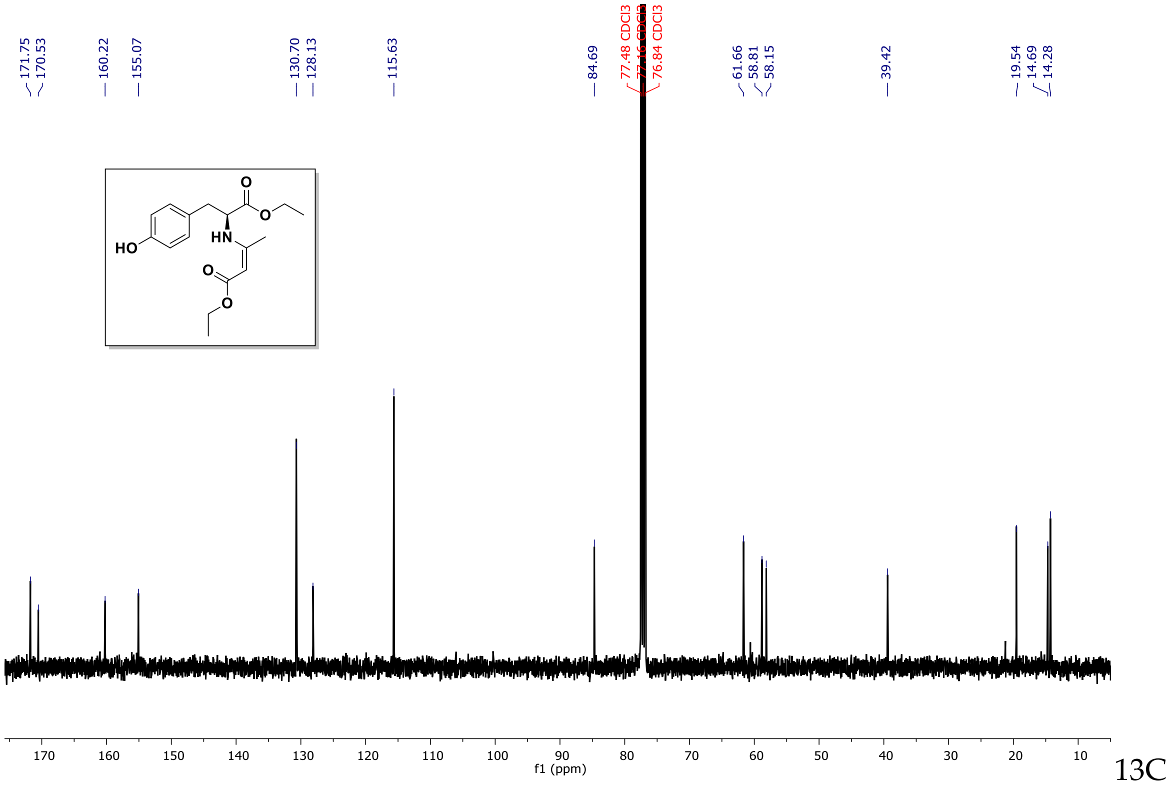

13C NMR spectrum of 26 in CDCl3.

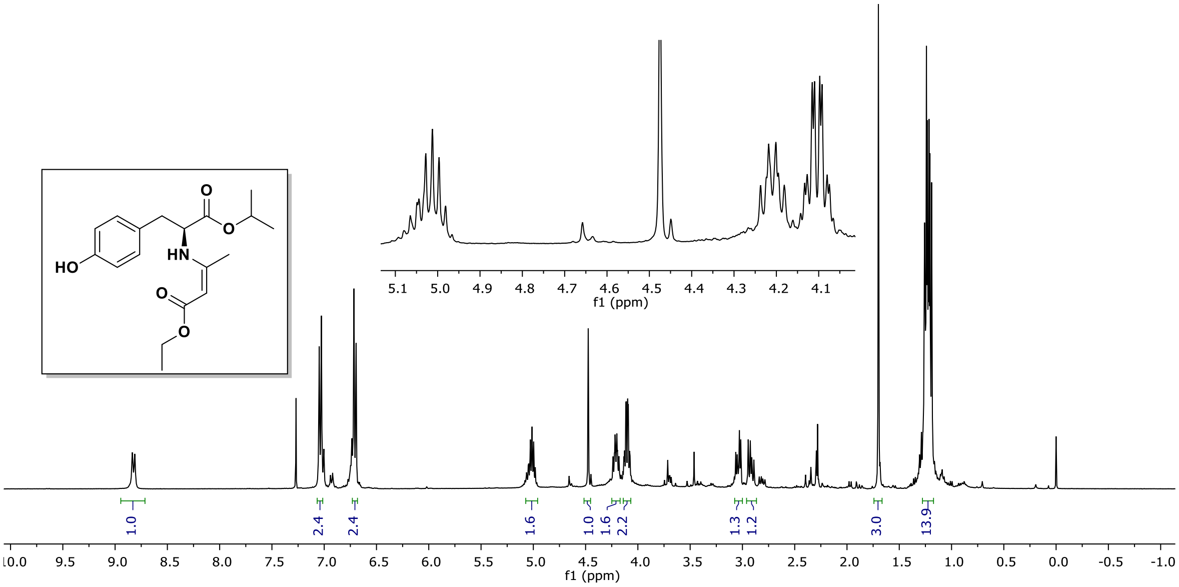

1H NMR spectrum of 27 in CDCl3.

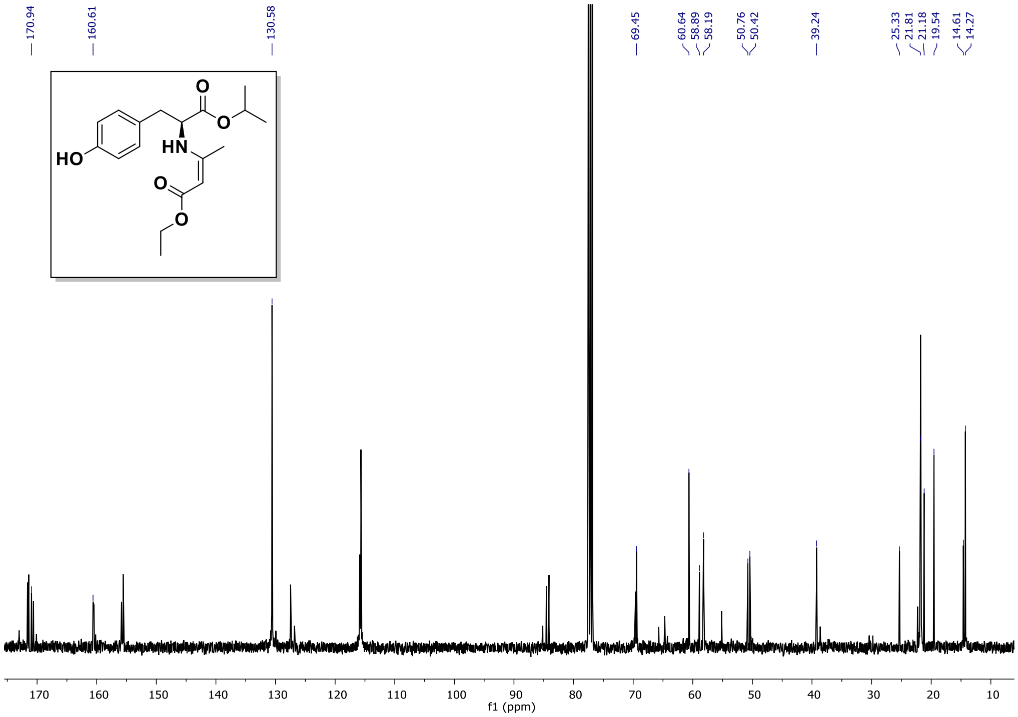

13C NMR spectrum of 27 in CDCl3.

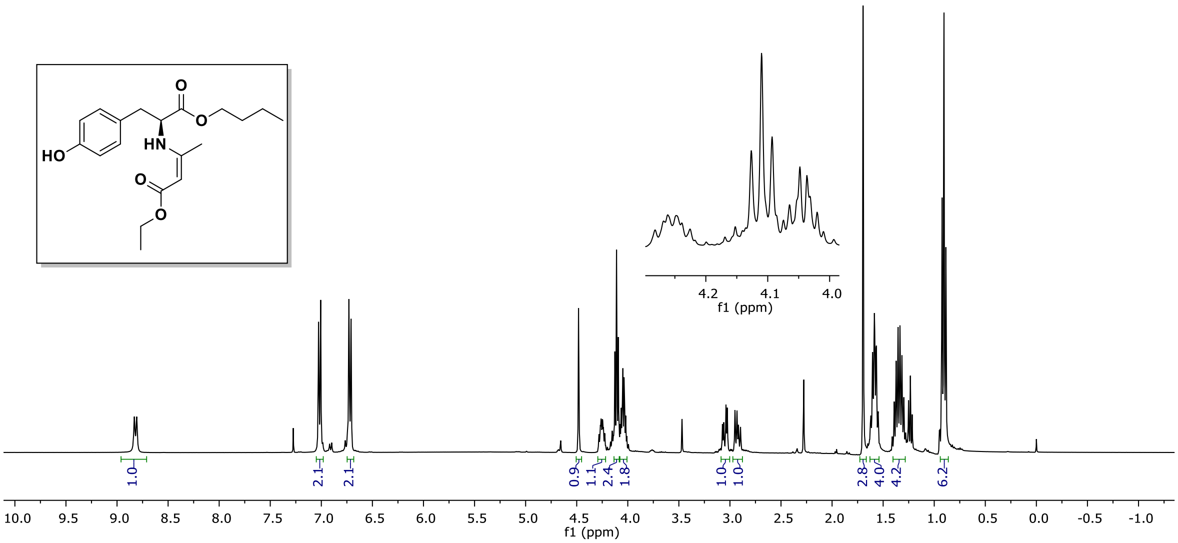

1H NMR spectrum of 28 in CDCl3.

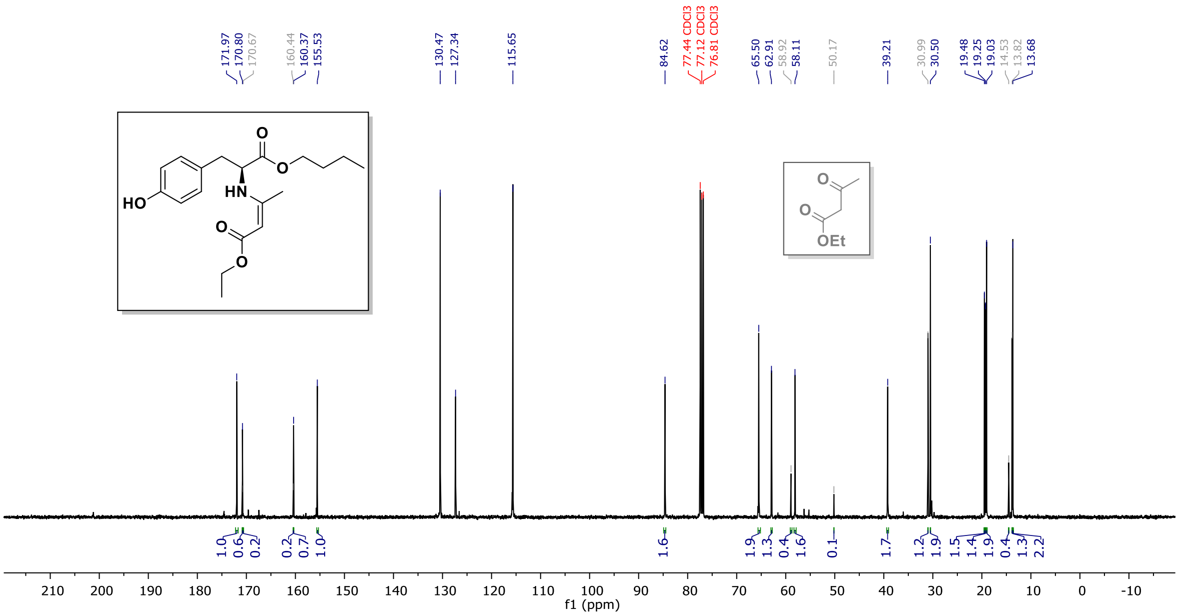

13C NMR spectrum of 28 in CDCl3.

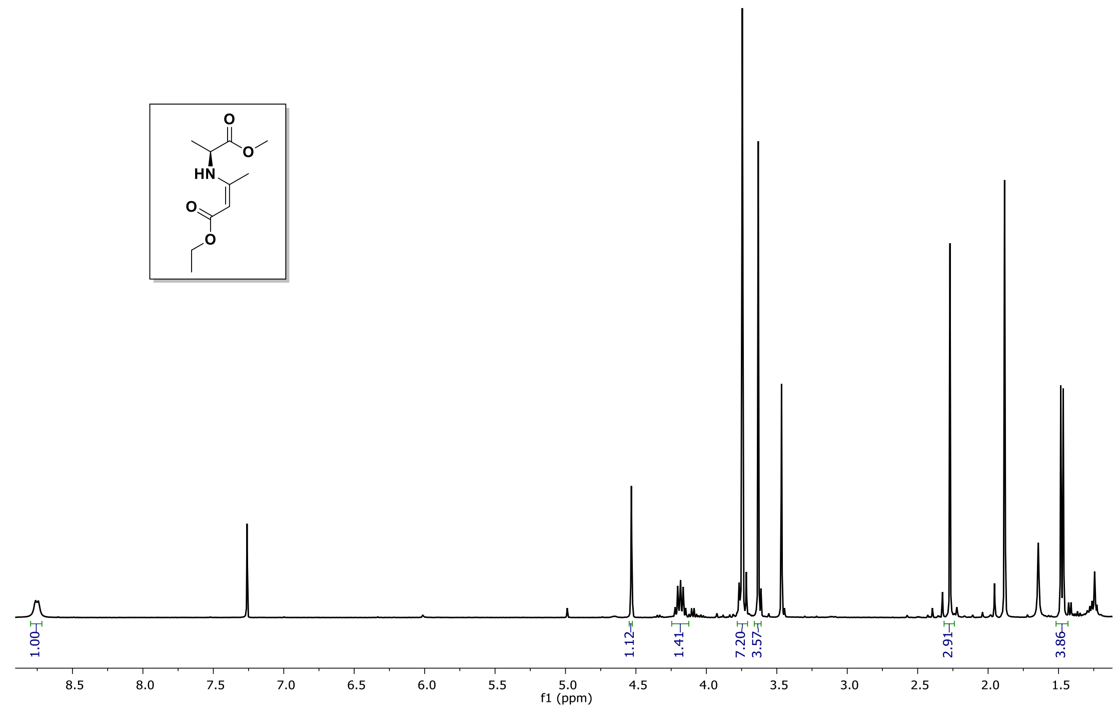

1H

^{1}H NMR spectrum of 29 in $CDCl_3$.

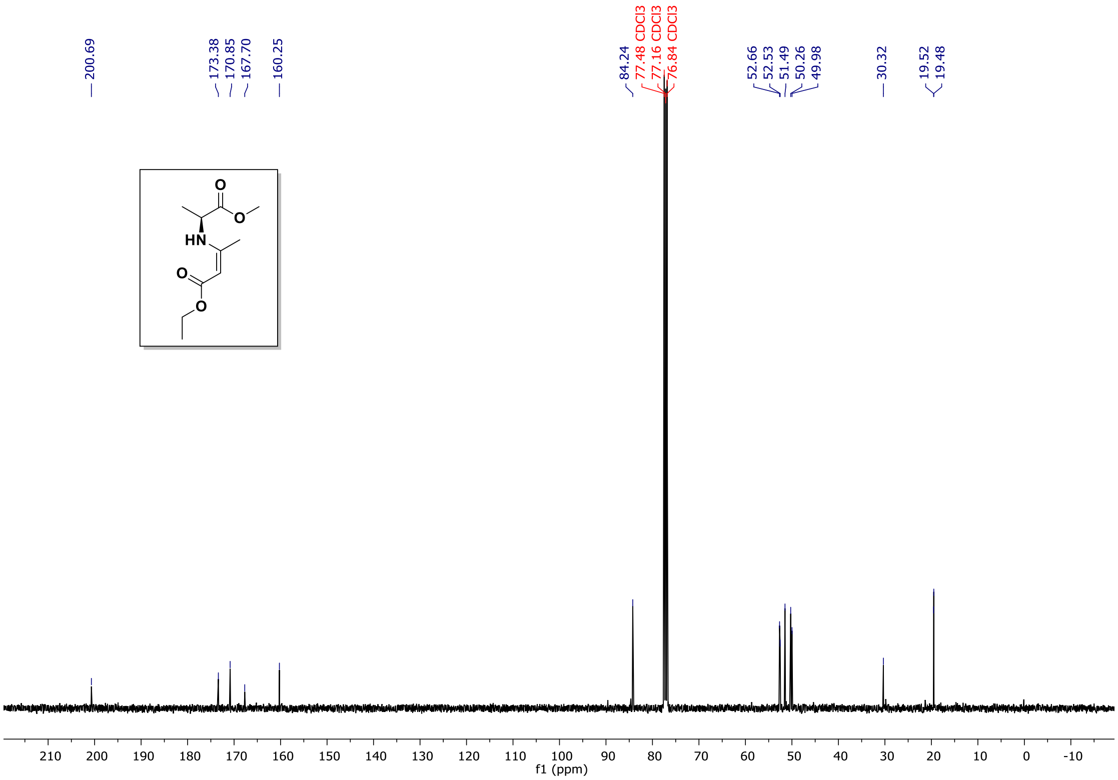

13C

^{13}C NMR spectrum of 29 in $CDCl_3$.

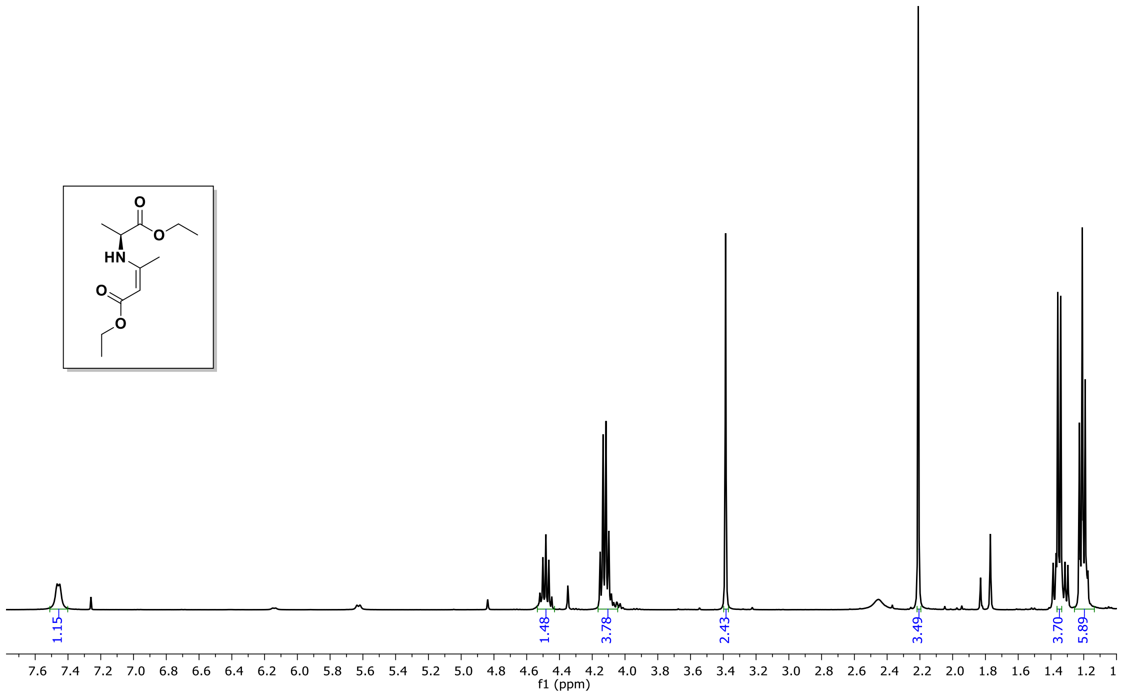

^{1}H NMR spectrum of 30 in $CDCl_3$.

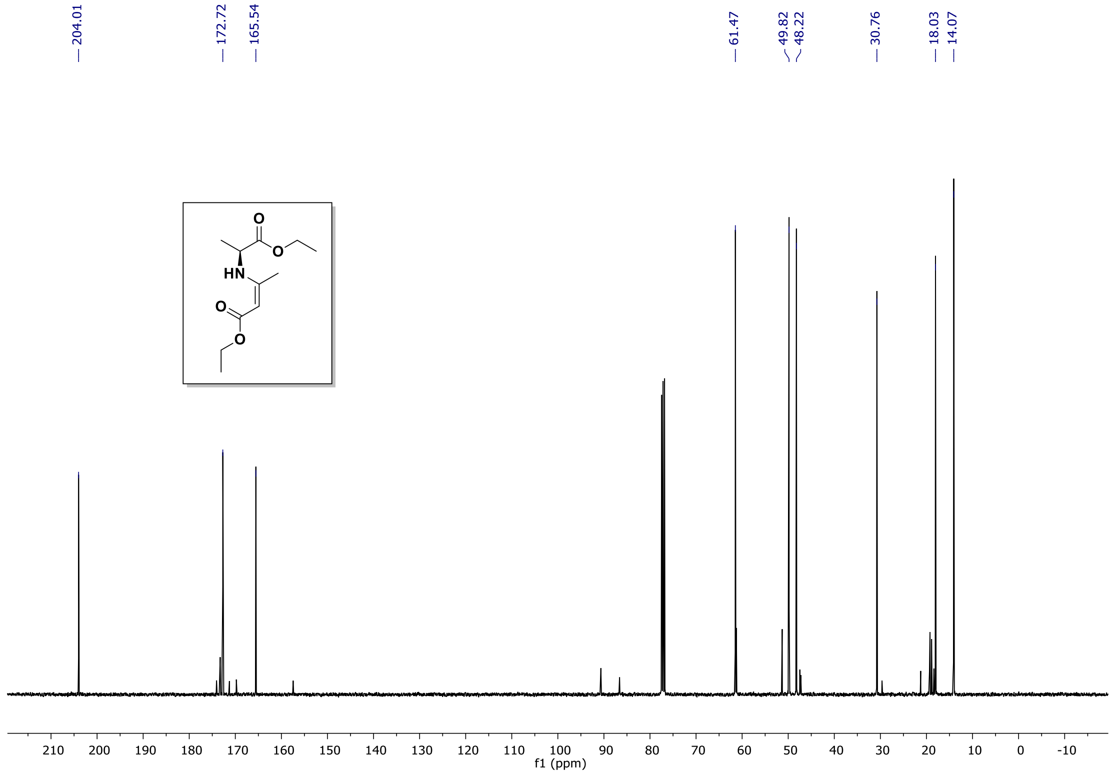

13C

^{13}C NMR spectrum of 30 in $CDCl_3$.

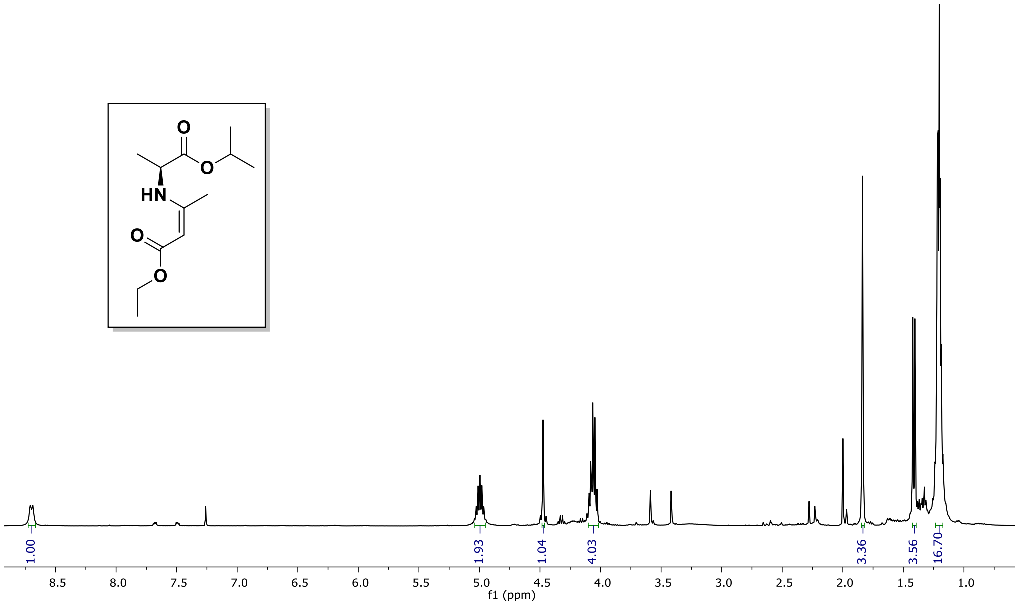

1H NMR spectrum of 31 in $CDCl_3$.

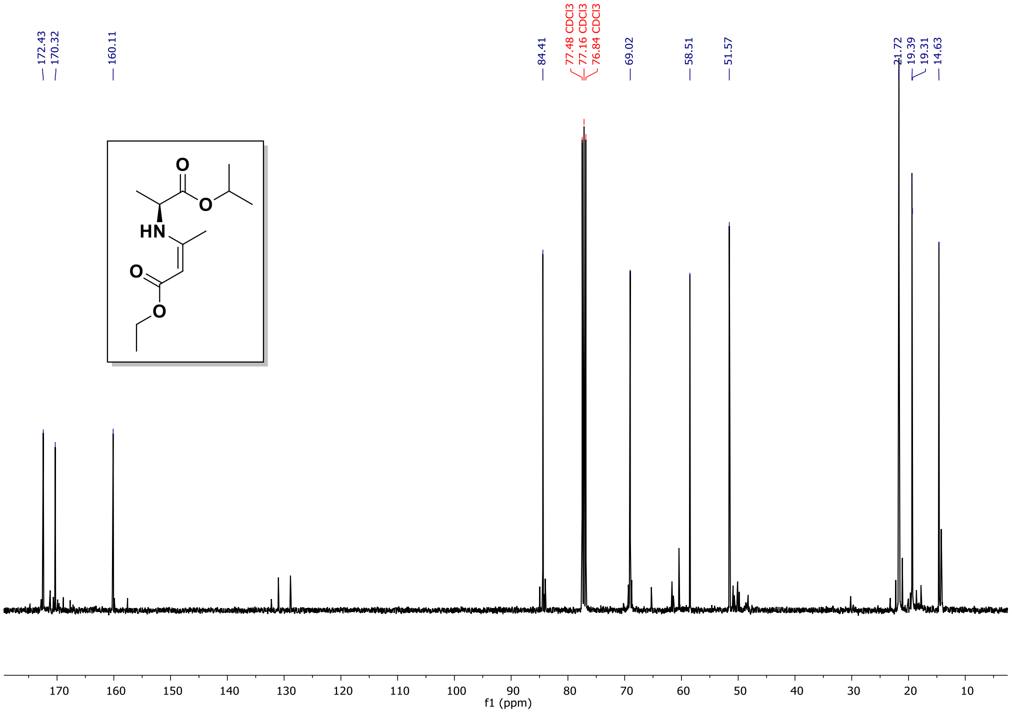

13C NMR spectrum of 31 in $CDCl_3$.

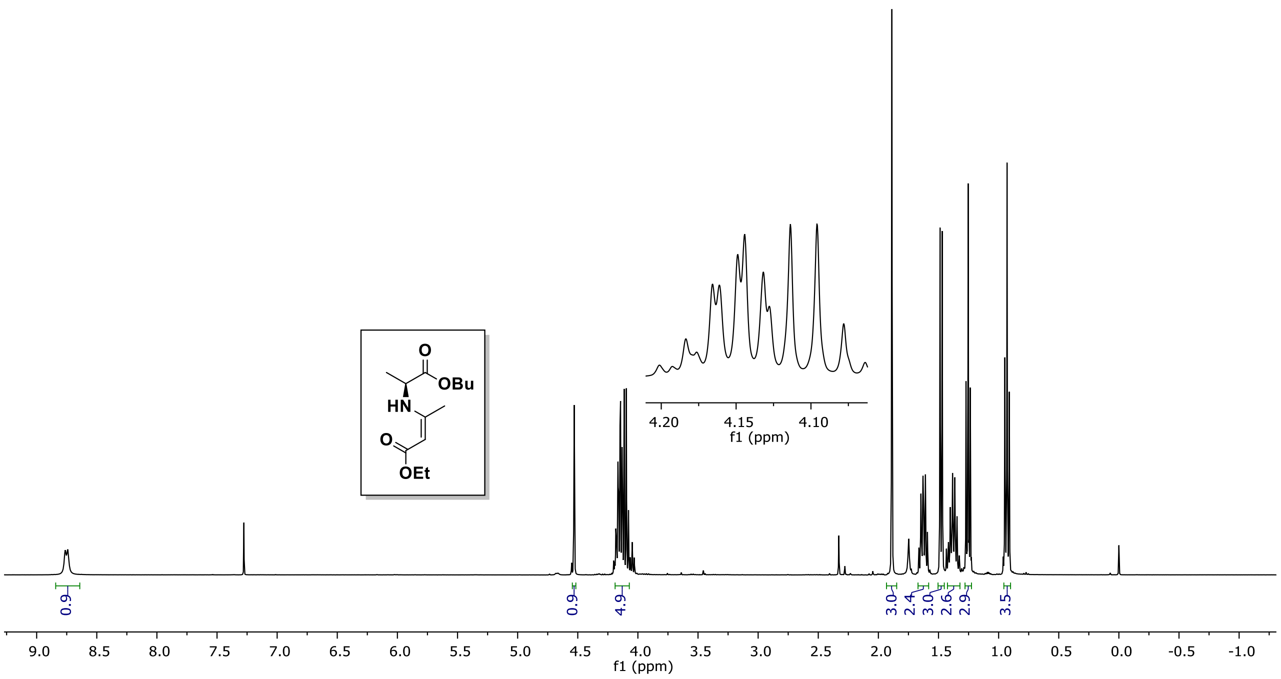

1H NMR spectrum of 32 in $CDCl_3$.

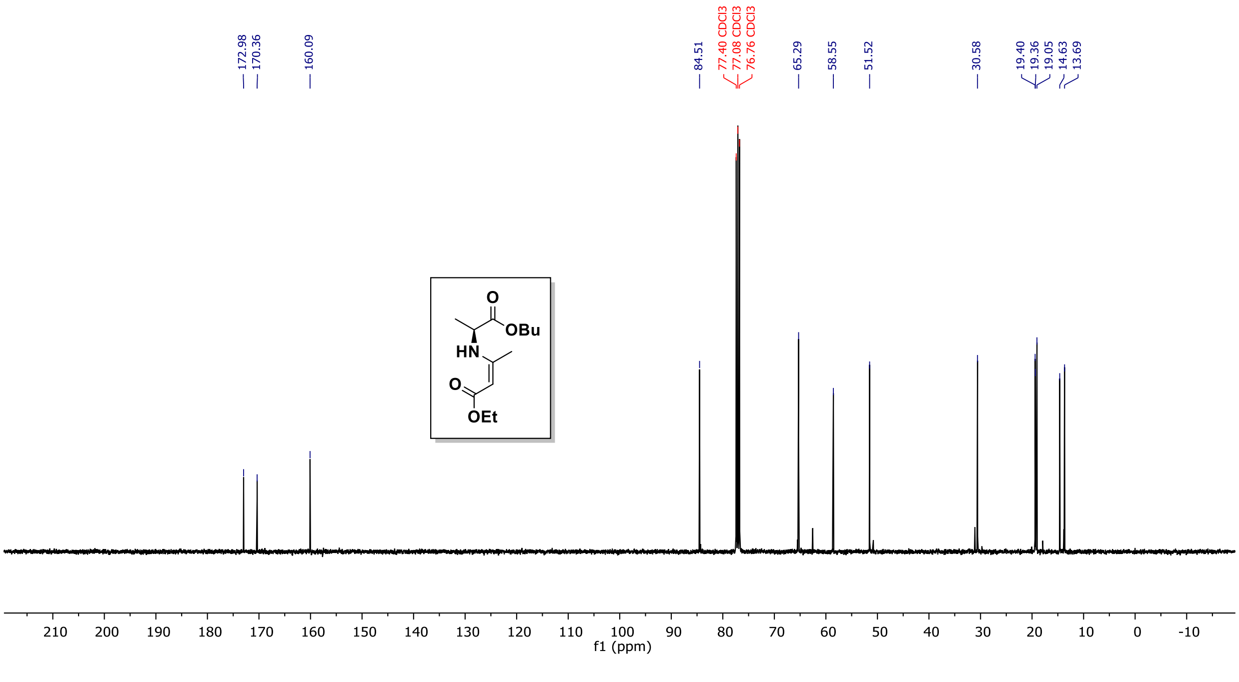

13C NMR spectrum of 32 in $CDCl_3$.

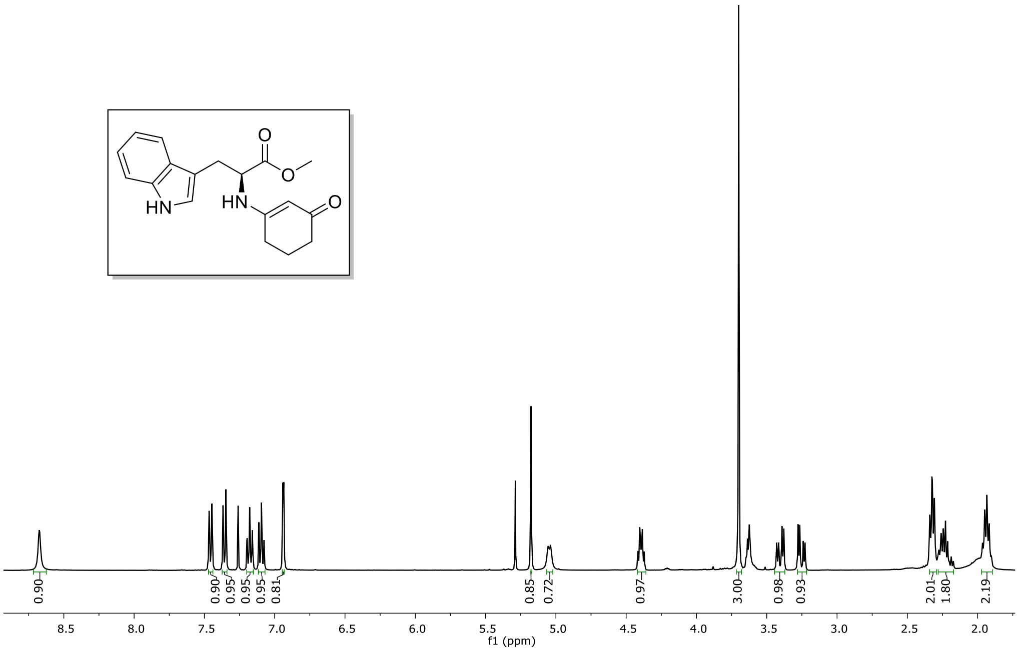

1H NMR spectrum of 33 in CDCl3.

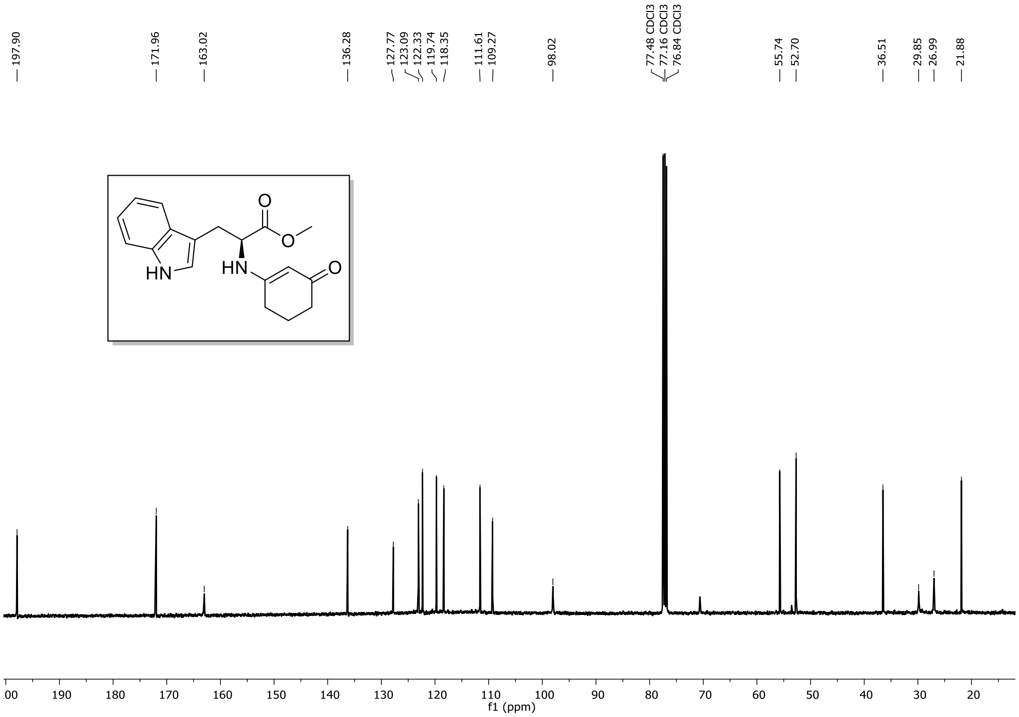

13C NMR spectrum of 33 in CDCl3.

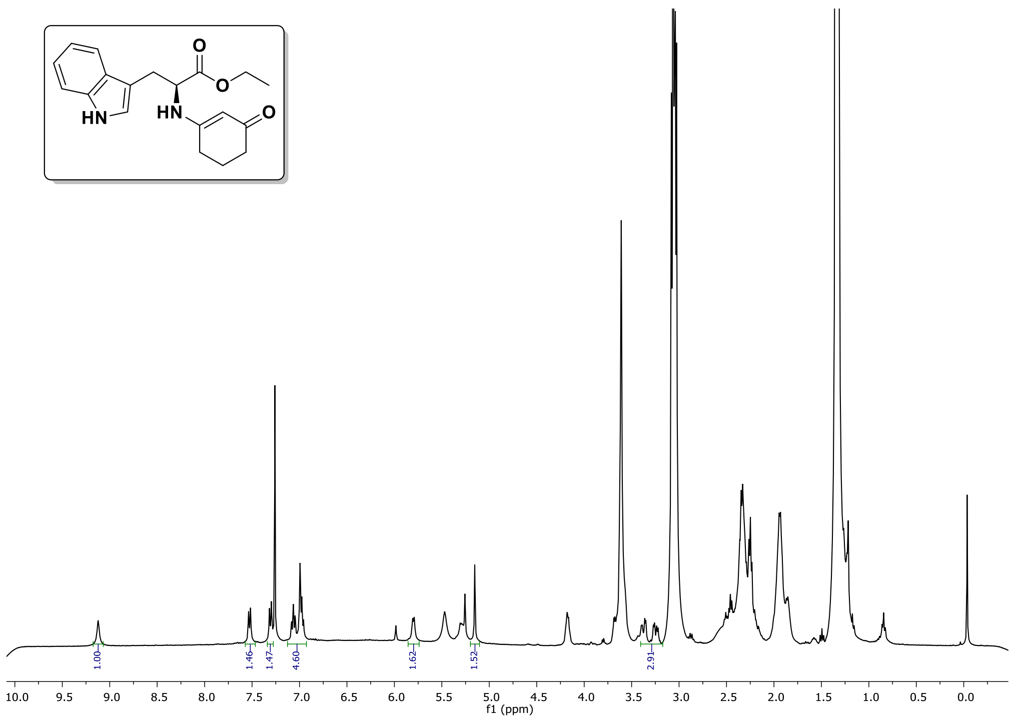

1H NMR spectrum of 34 in $CDCl_3$.

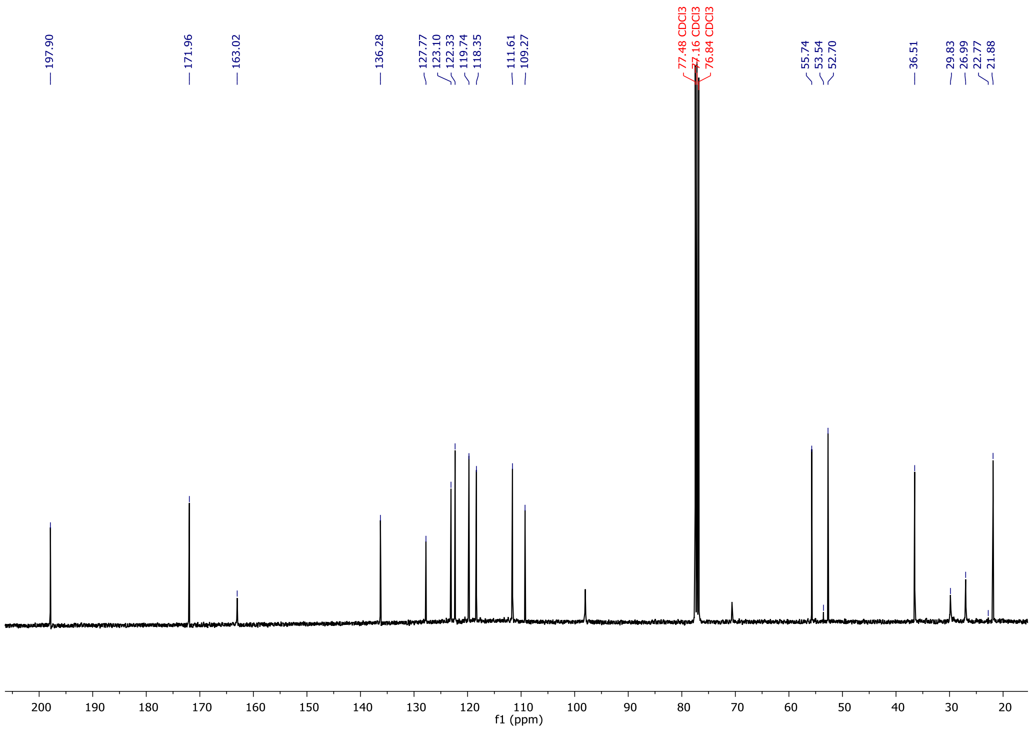

13C NMR spectrum of 34 in $CDCl_3$.

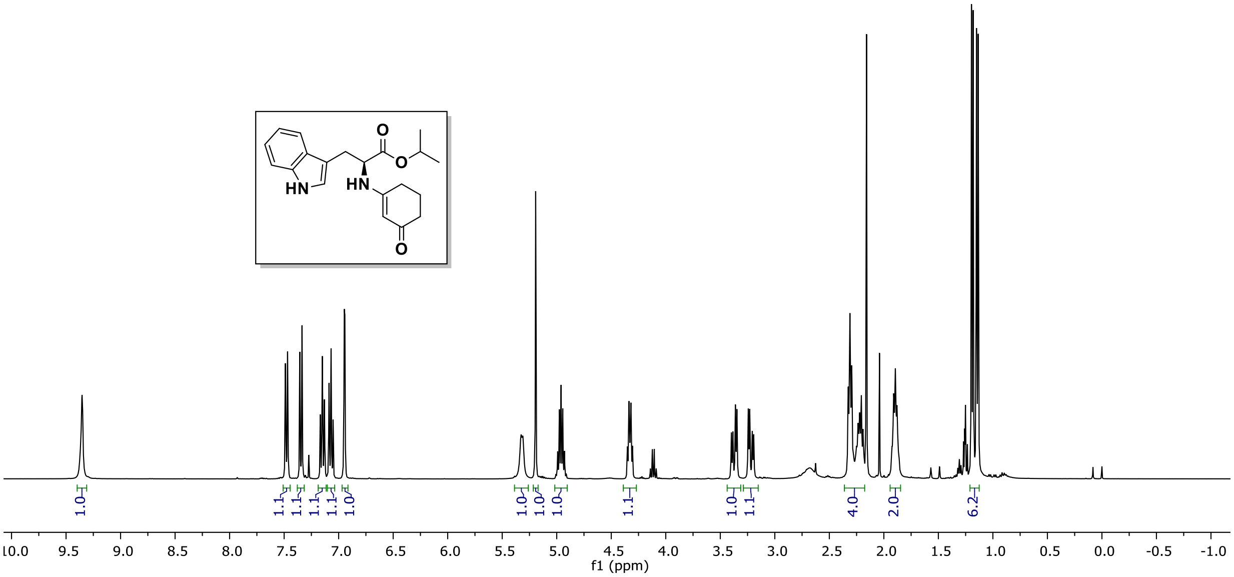

1H NMR spectrum of 35 in CDCl3.

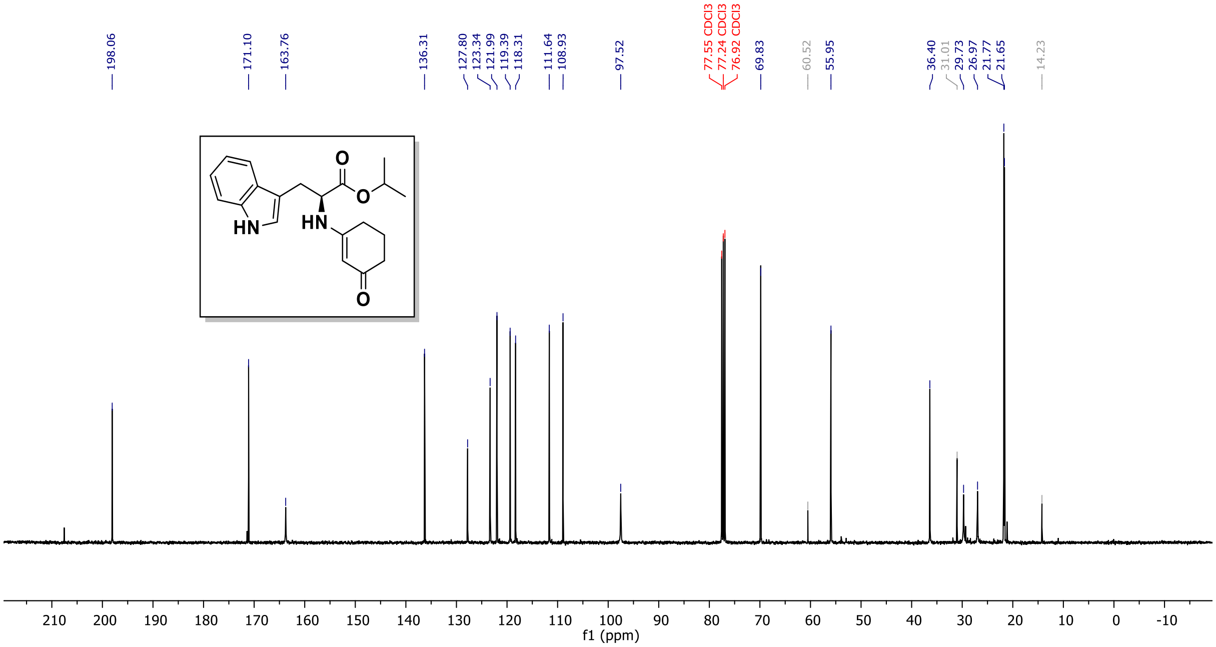

13C NMR spectrum of 35 in CDCl3.

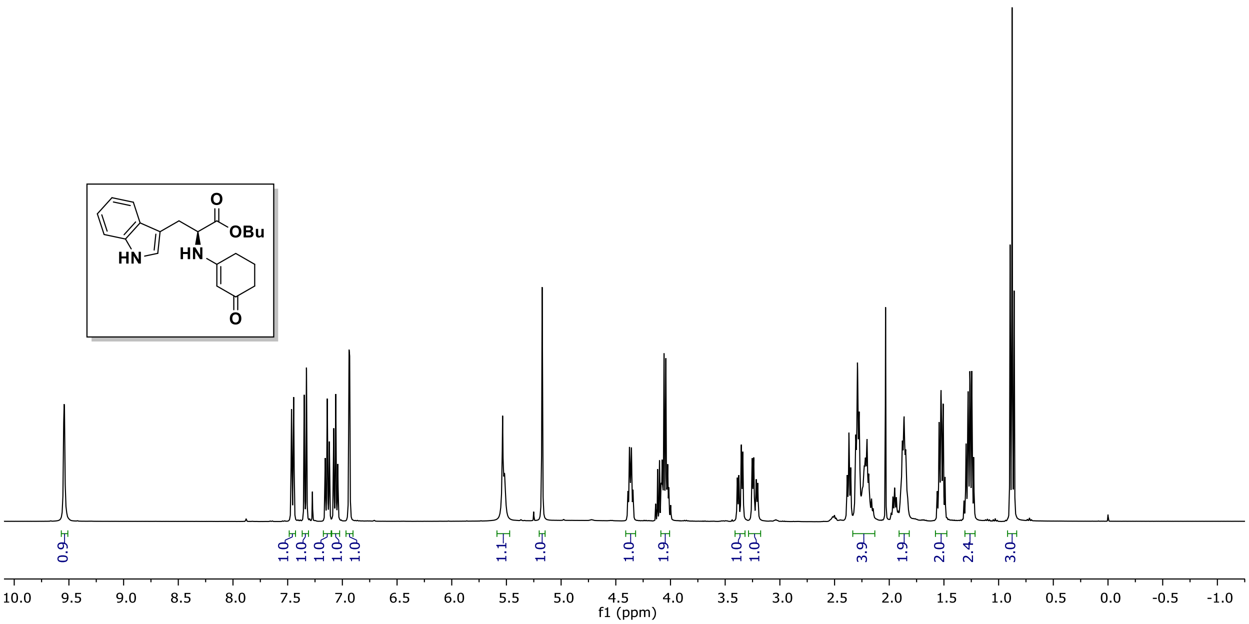

1H NMR spectrum of 36 in $CDCl_3$.

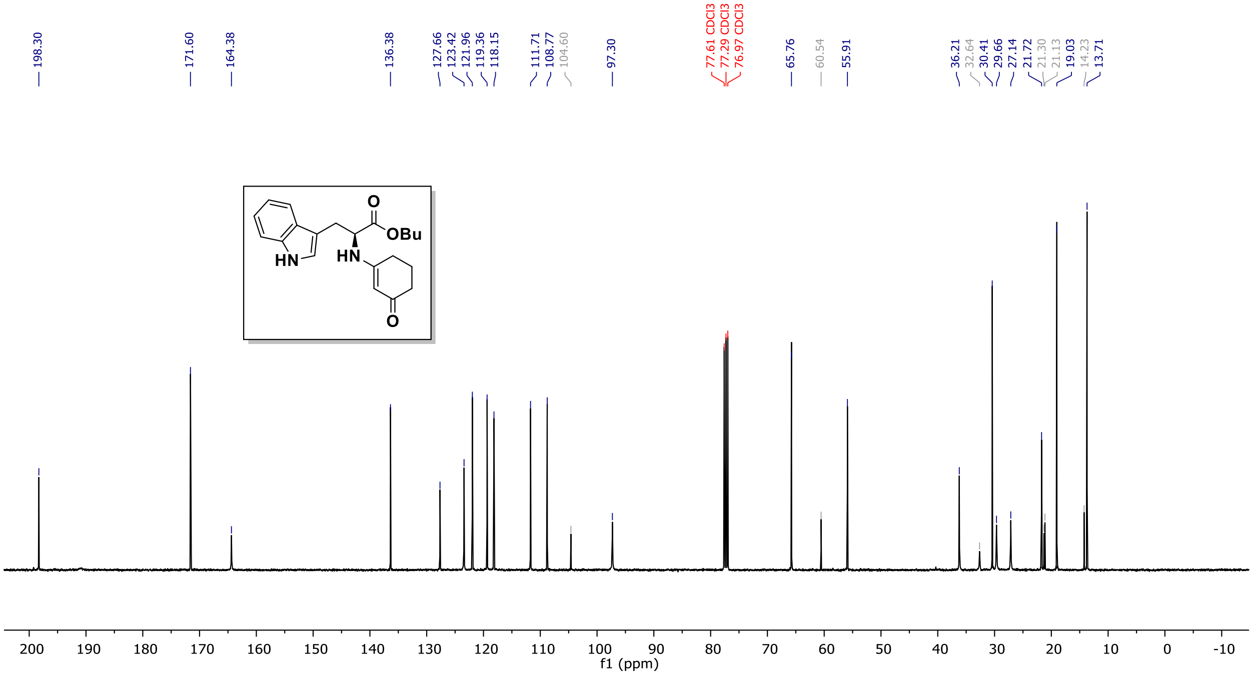

13C NMR spectrum of 36 in $CDCl_3$.

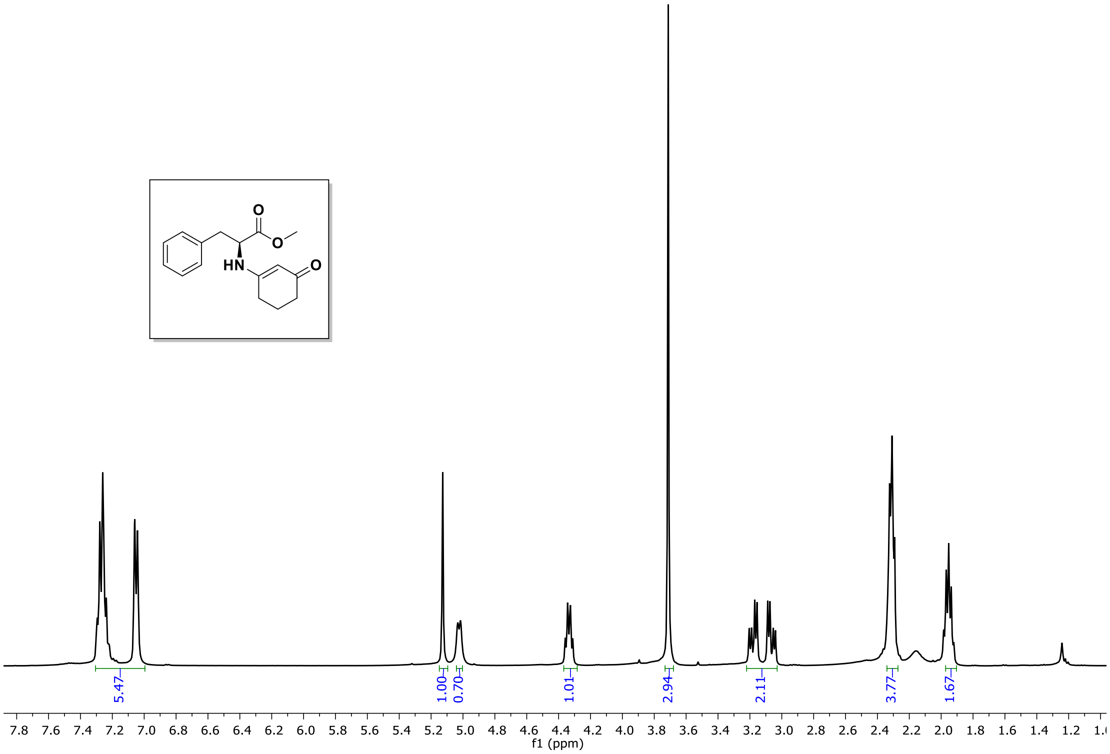

1H

NMR spectrum of 37 in $CDCl_3$.

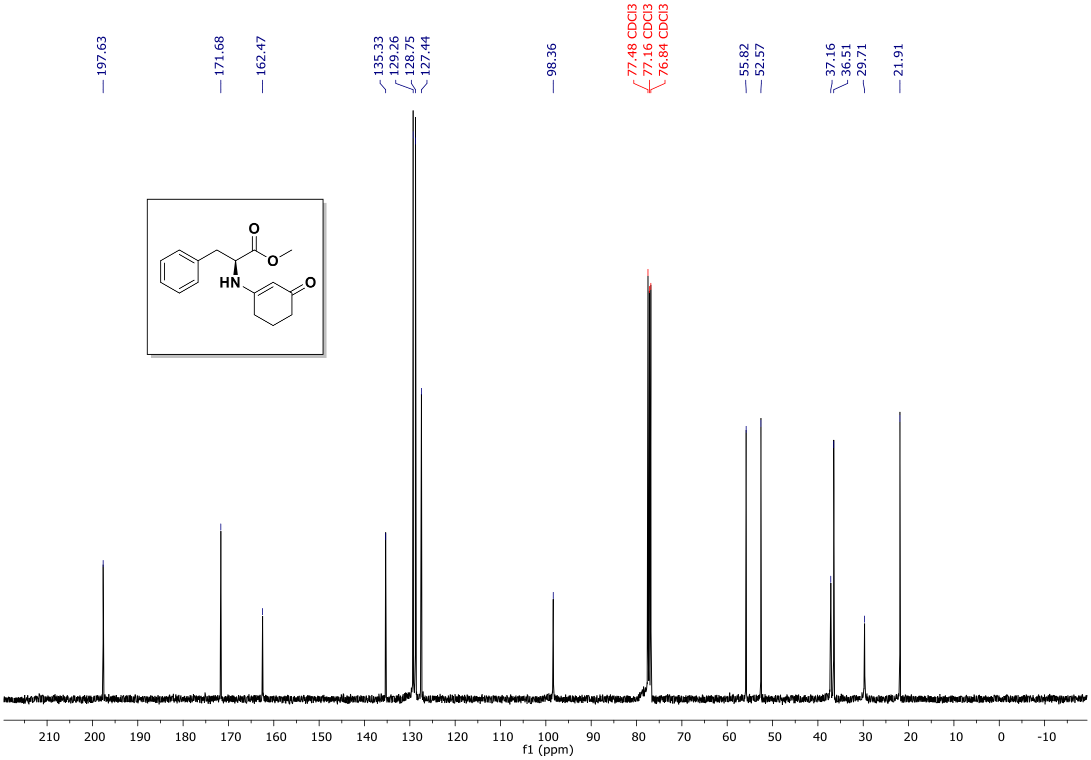

13C

NMR spectrum of 37 in $CDCl_3$.

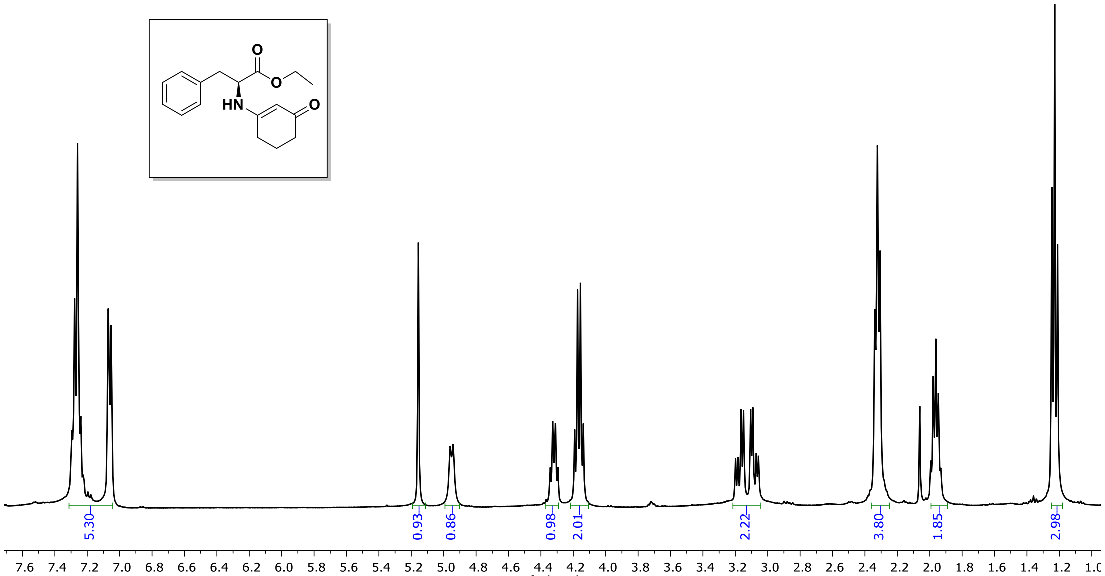

1H NMR spectrum of 38 in $CDCl_3$.

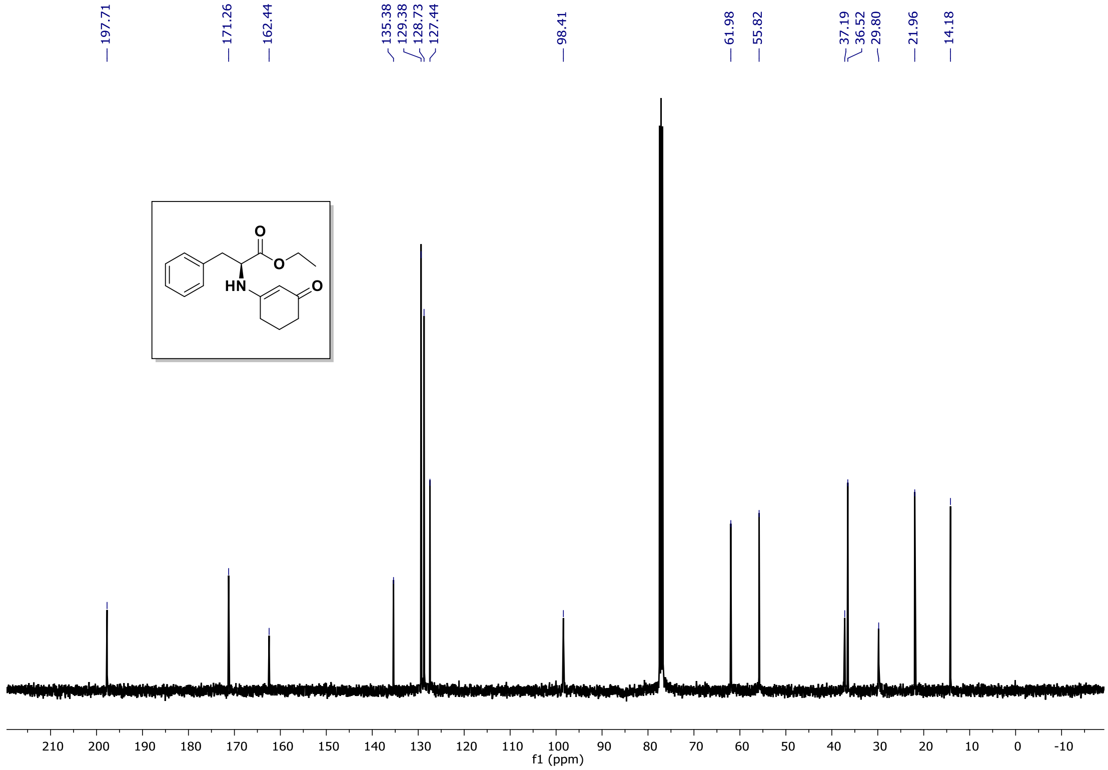

13C NMR spectrum of 38 in $CDCl_3$.

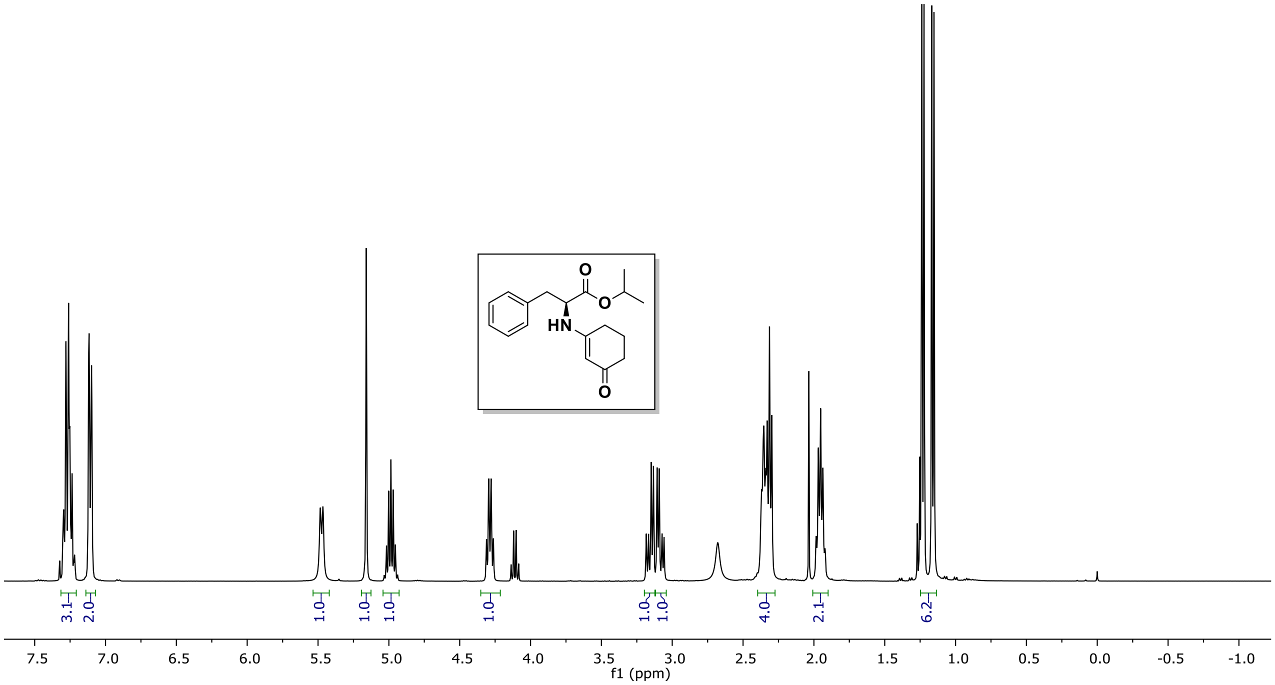

1H NMR spectrum of 39 in CDCl3.

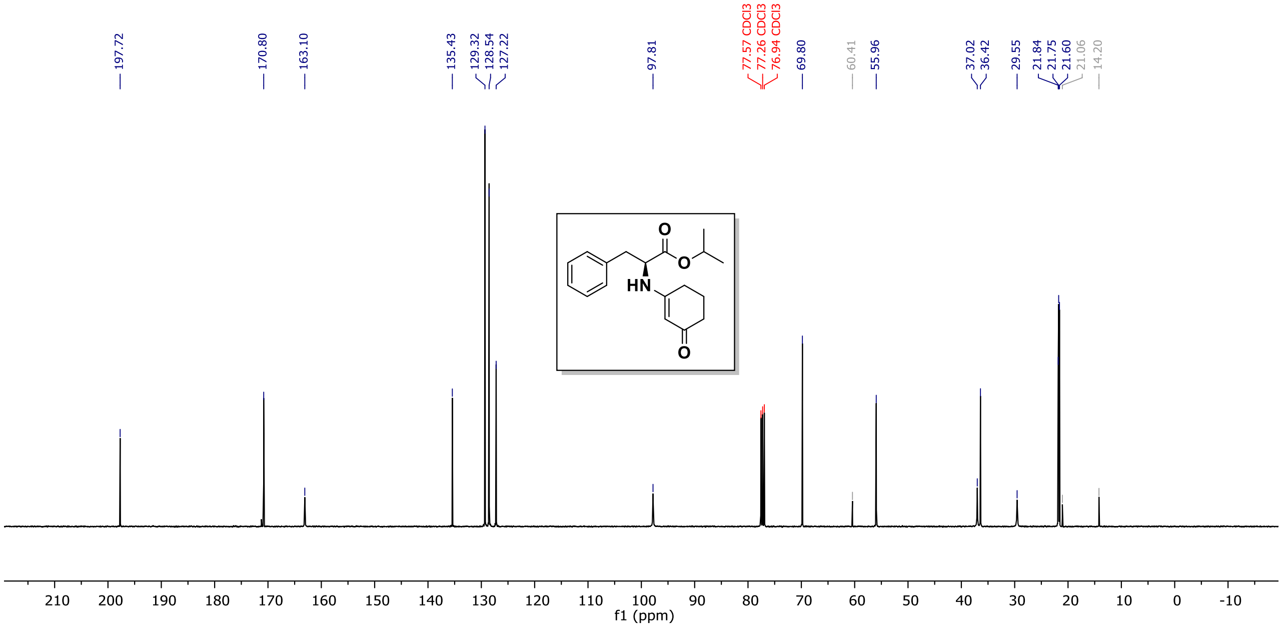

13C NMR spectrum of 39 in CDCl3.

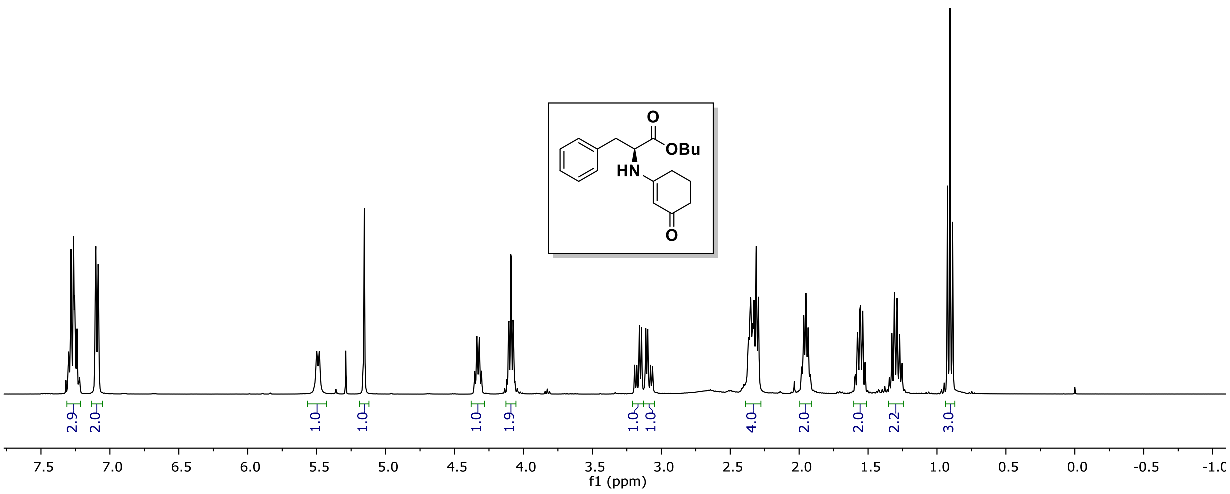

1H NMR spectrum of 40 in CDCl3.

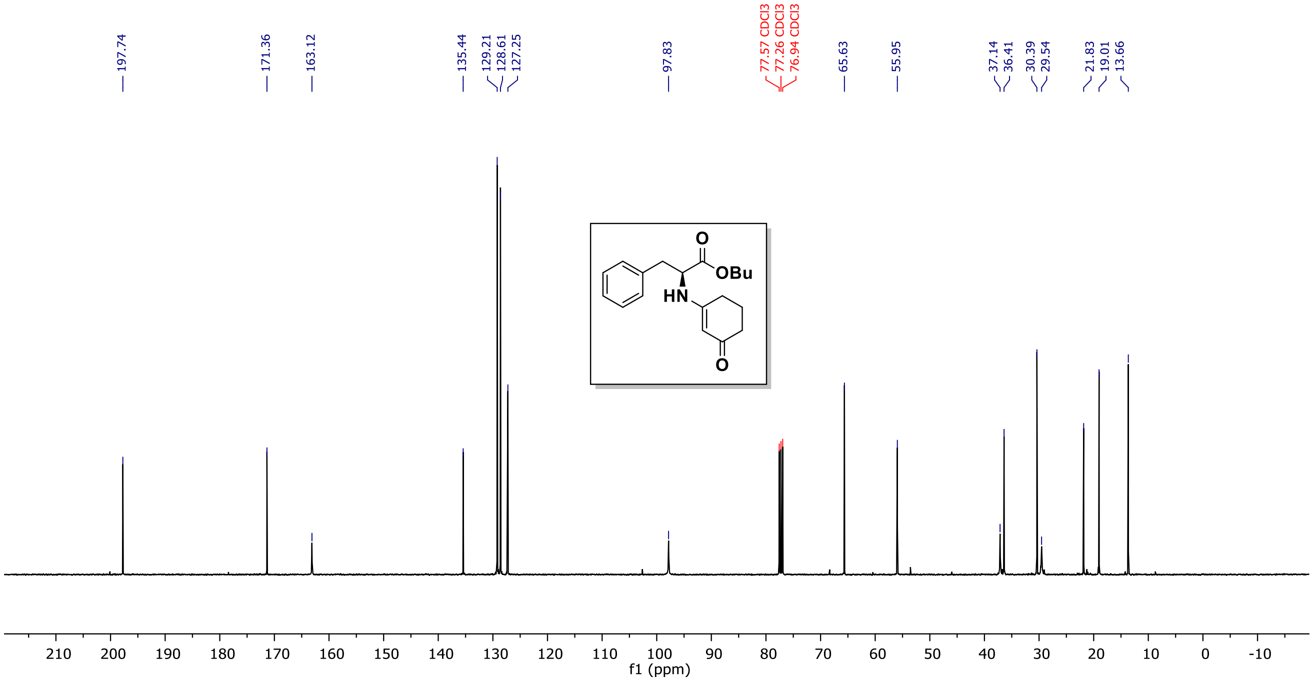

13C NMR spectrum of 40 in CDCl3.

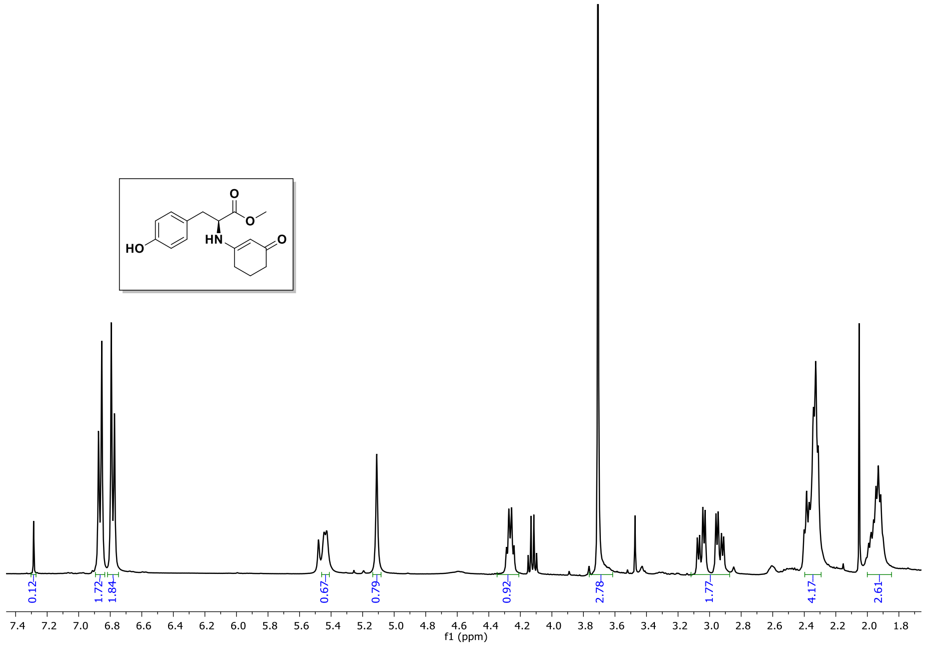

1H NMR spectrum of 41 in CDCl3.

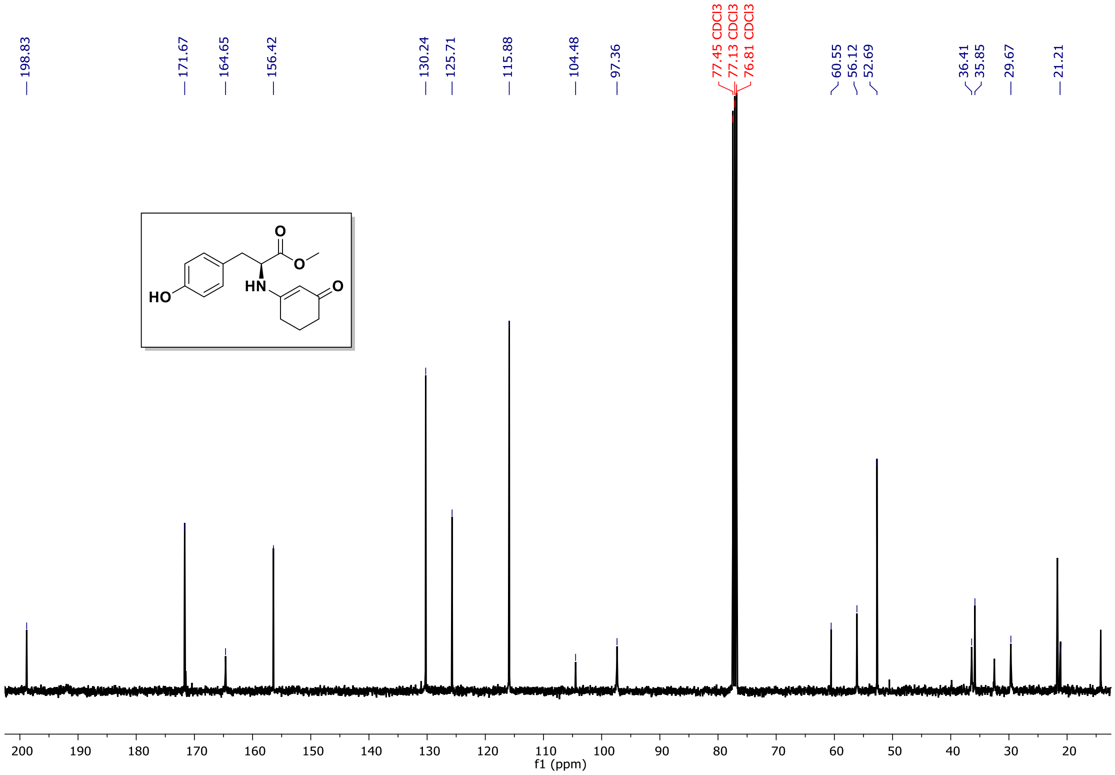

13C

13C NMR spectrum of 41 in CDCl3.

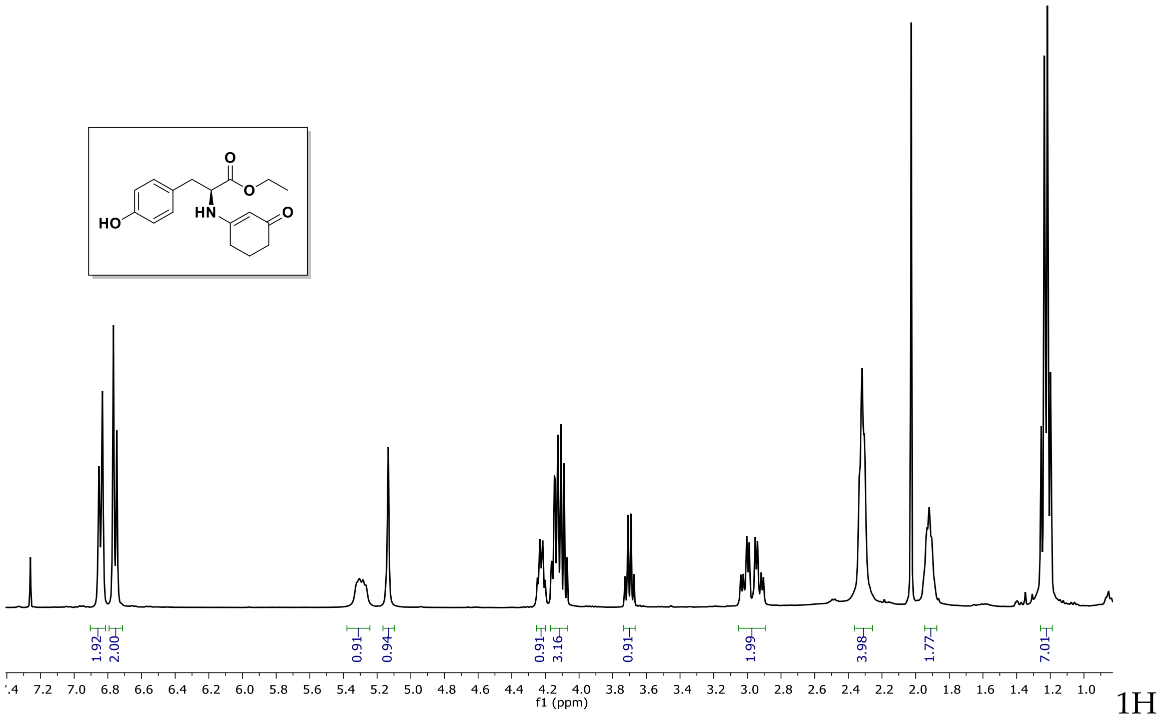

1H NMR spectrum of 42 in CDCl3.

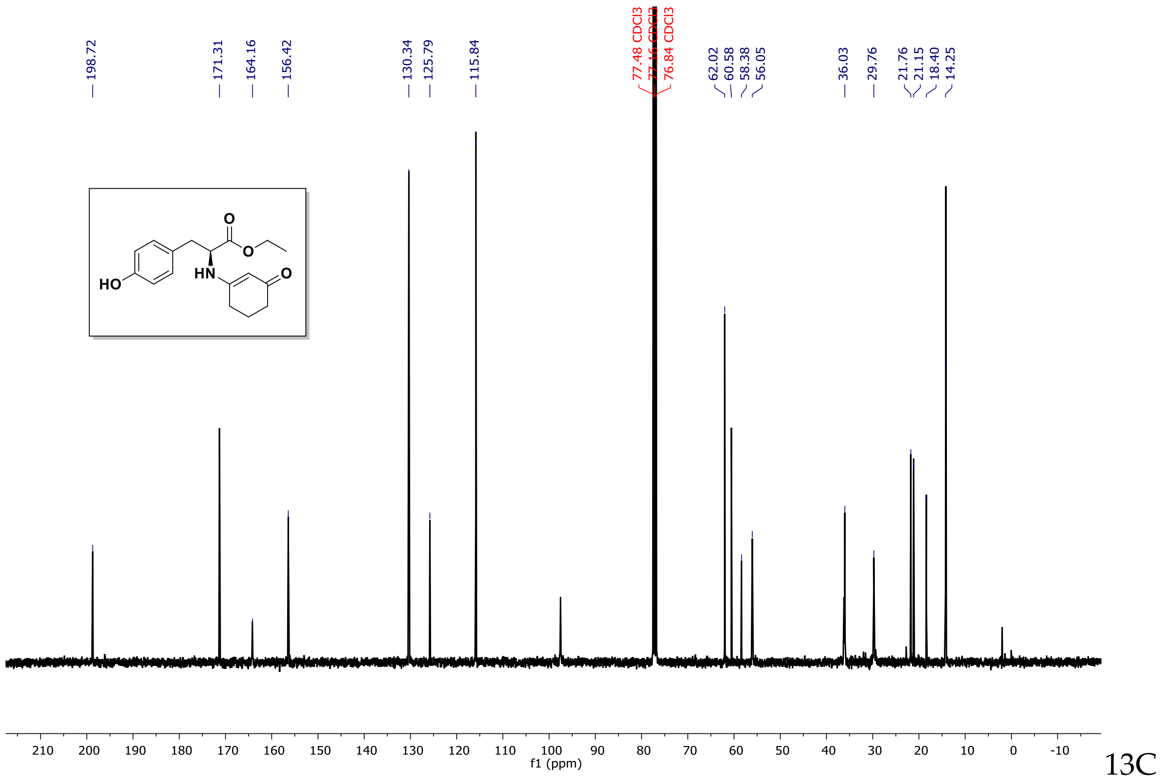

13C NMR spectrum of 42 in CDCl3.

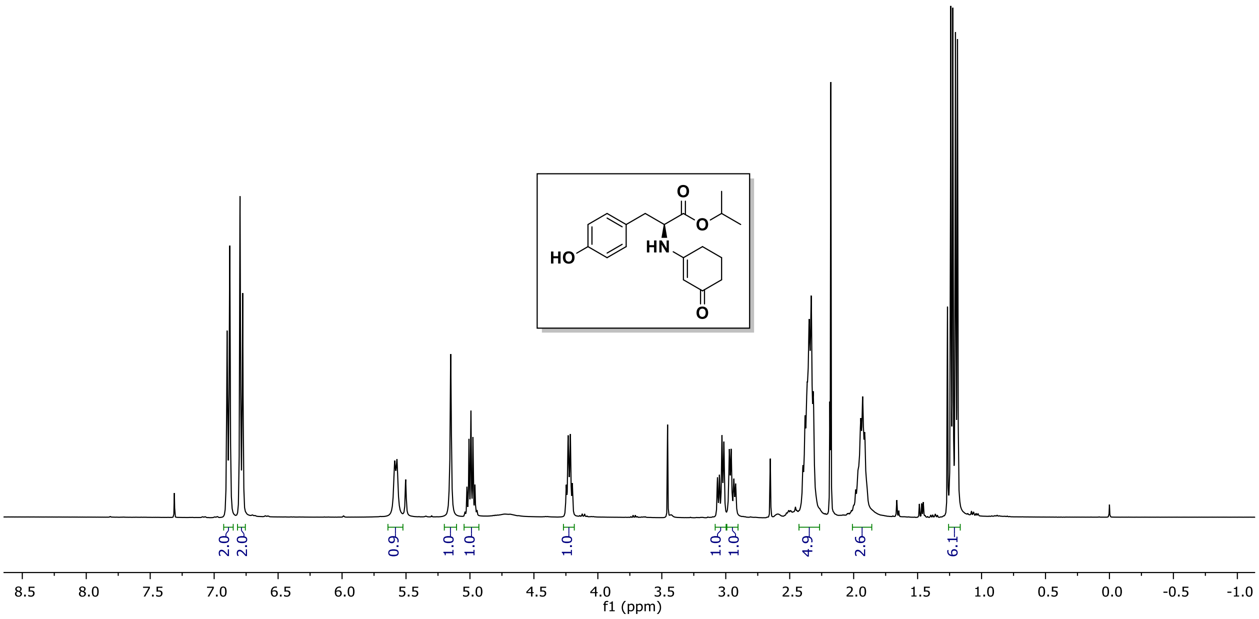

1H NMR spectrum of 43 in CDCl3.

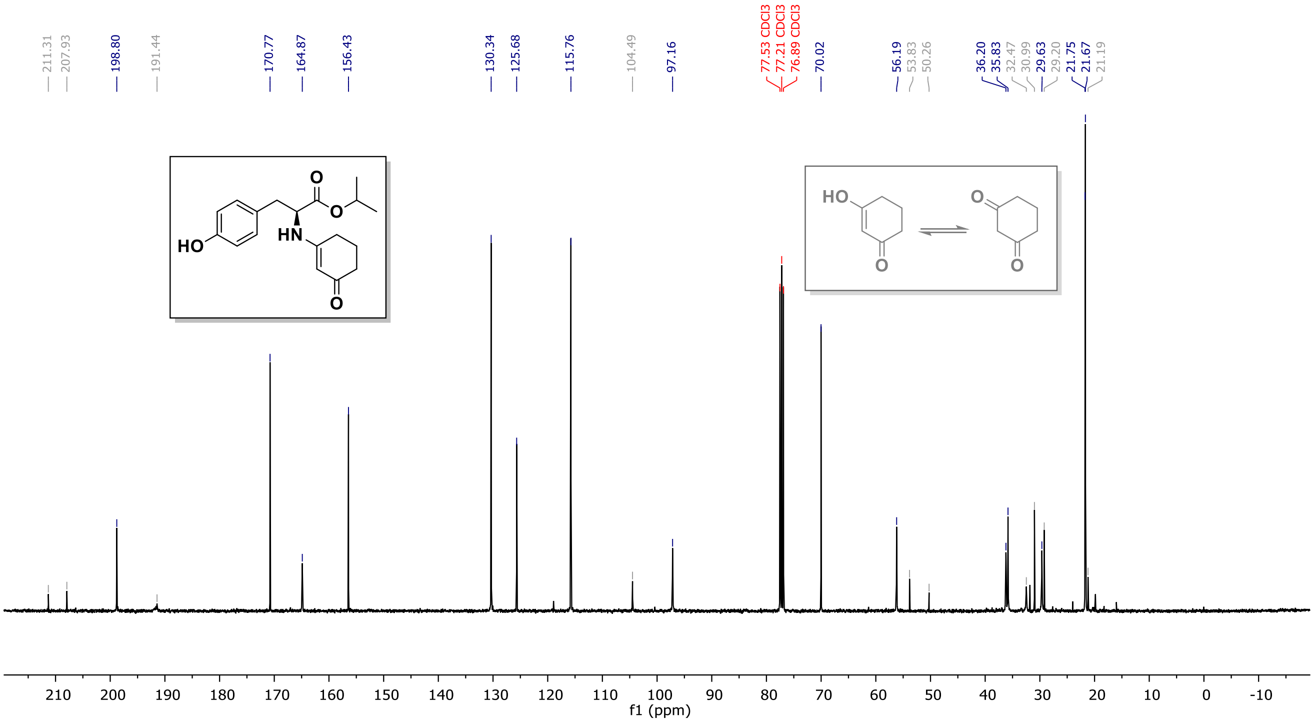

13C NMR spectrum of 43 in CDCl3.

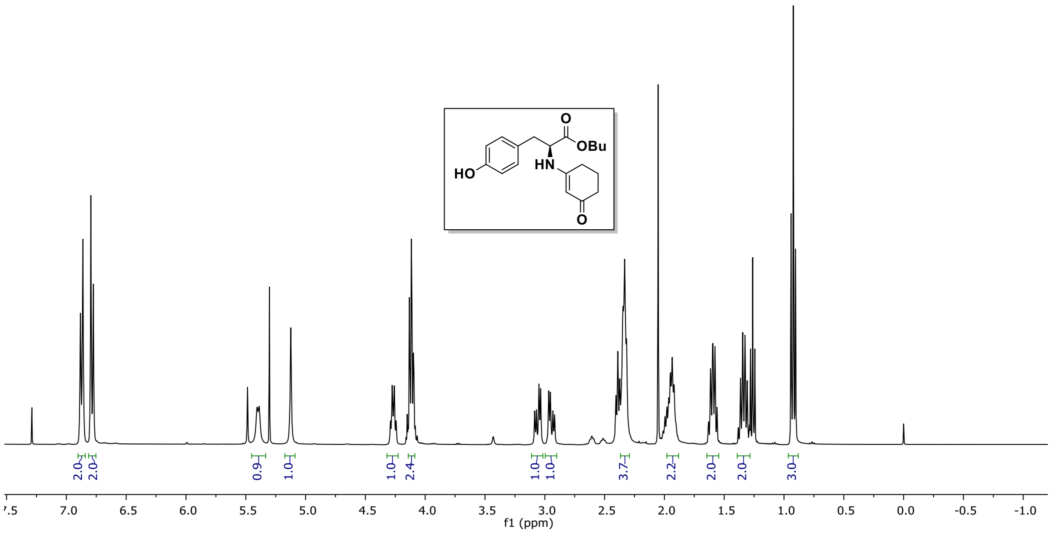

1H NMR spectrum of 44 in CDCl3.

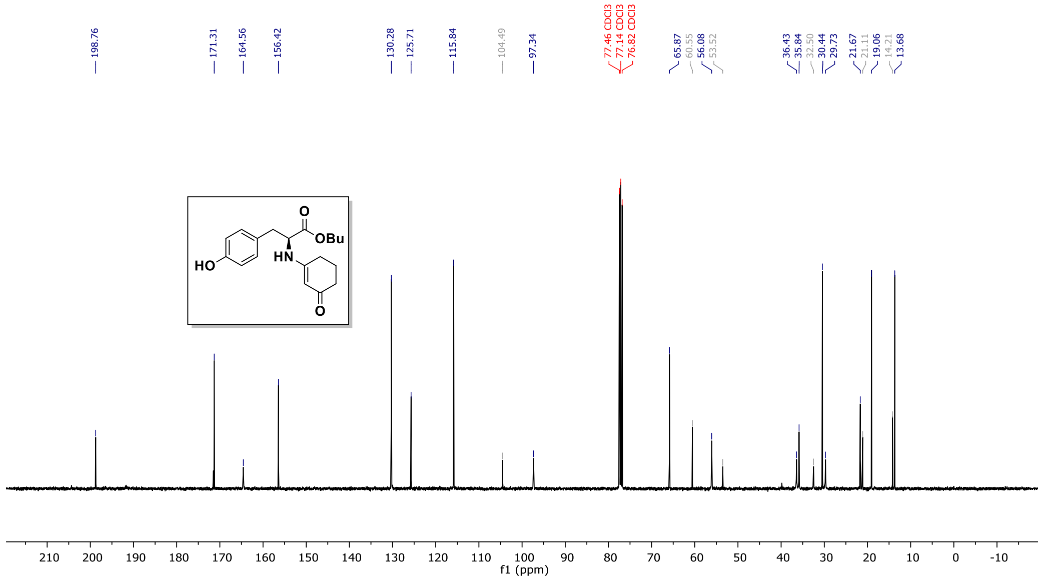

13C NMR spectrum of 44 in CDCl3.

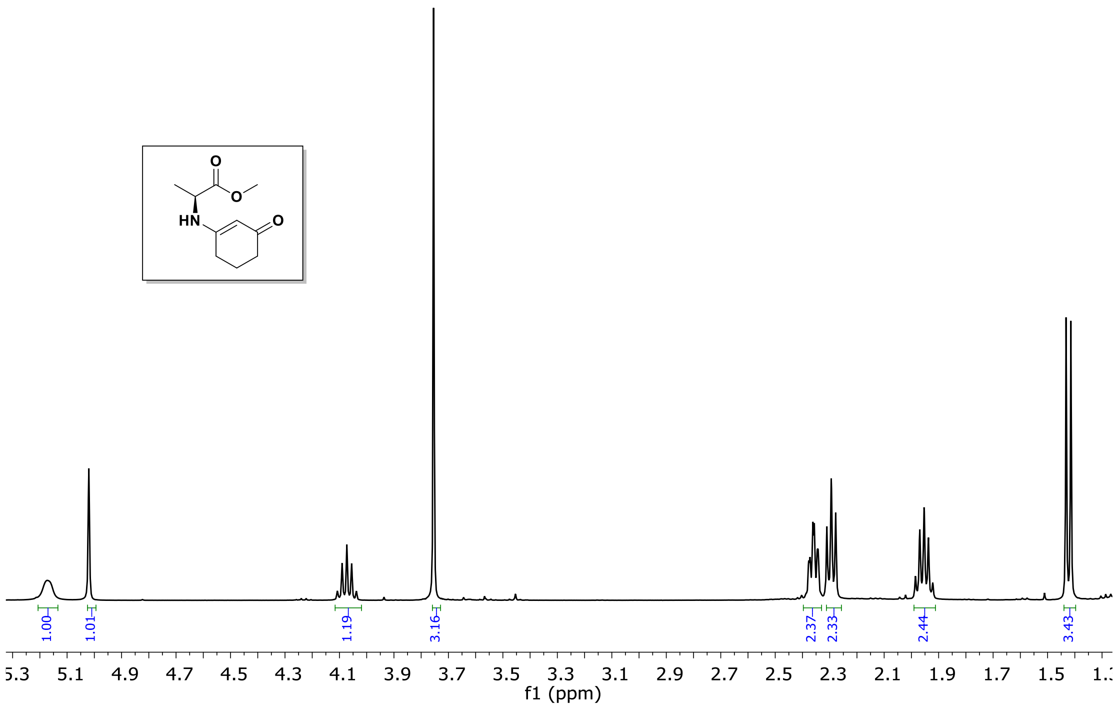

1H

1H NMR spectrum of 45 in $CDCl_3$.

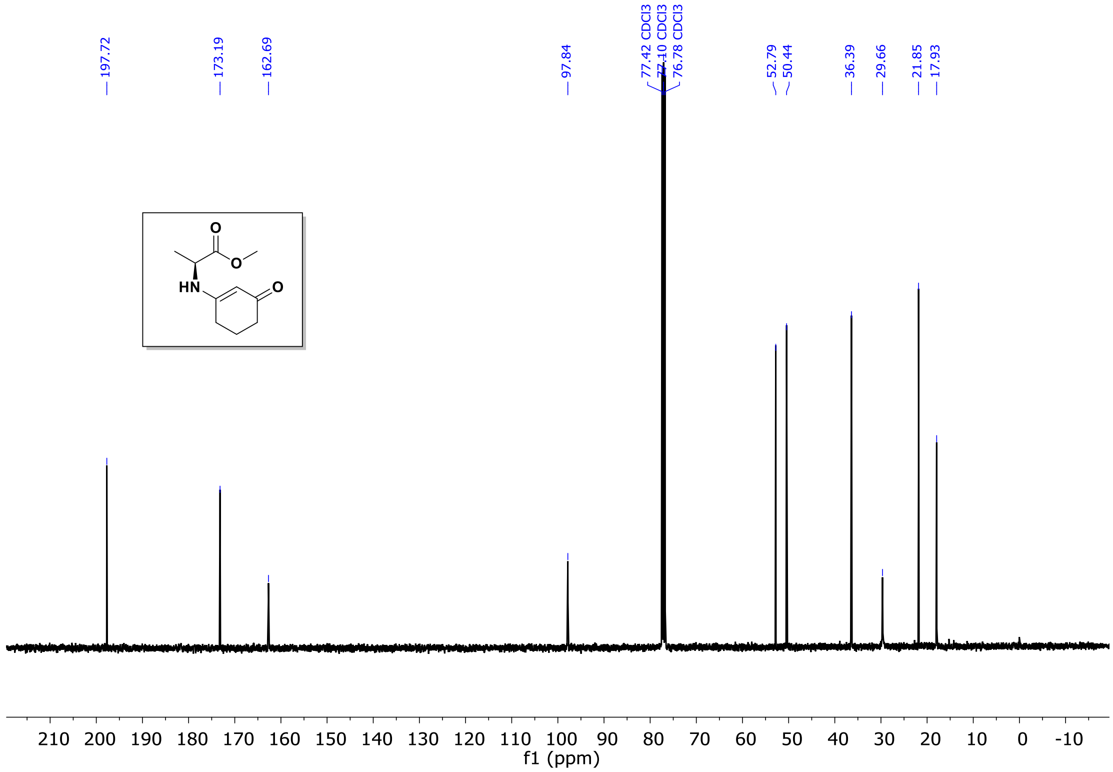

13C

13C NMR spectrum of 45 in $CDCl_3$.

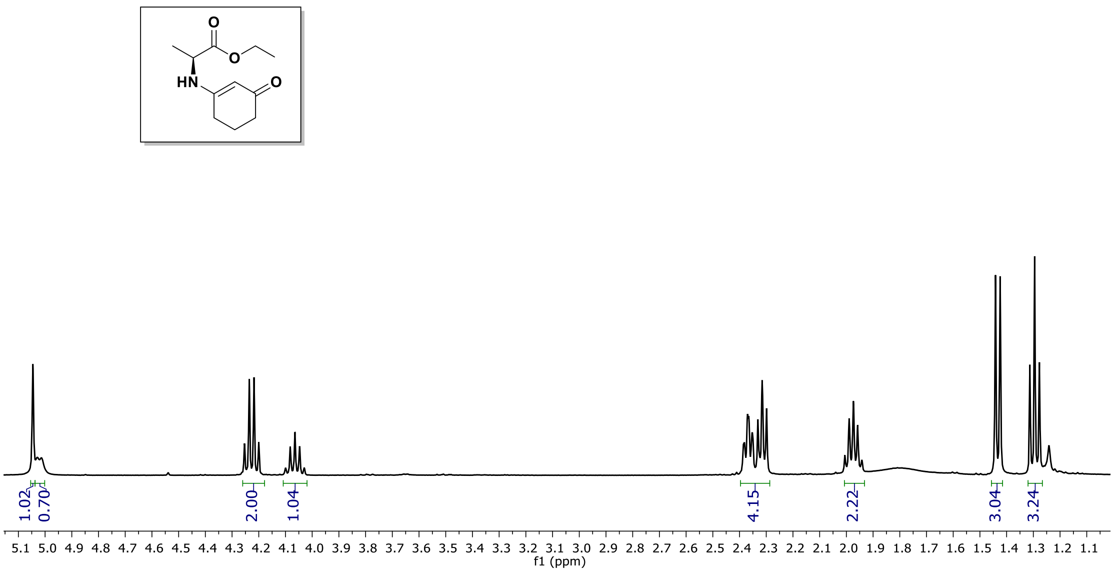

1H

1H NMR spectrum of 46 in $CDCl_3$.

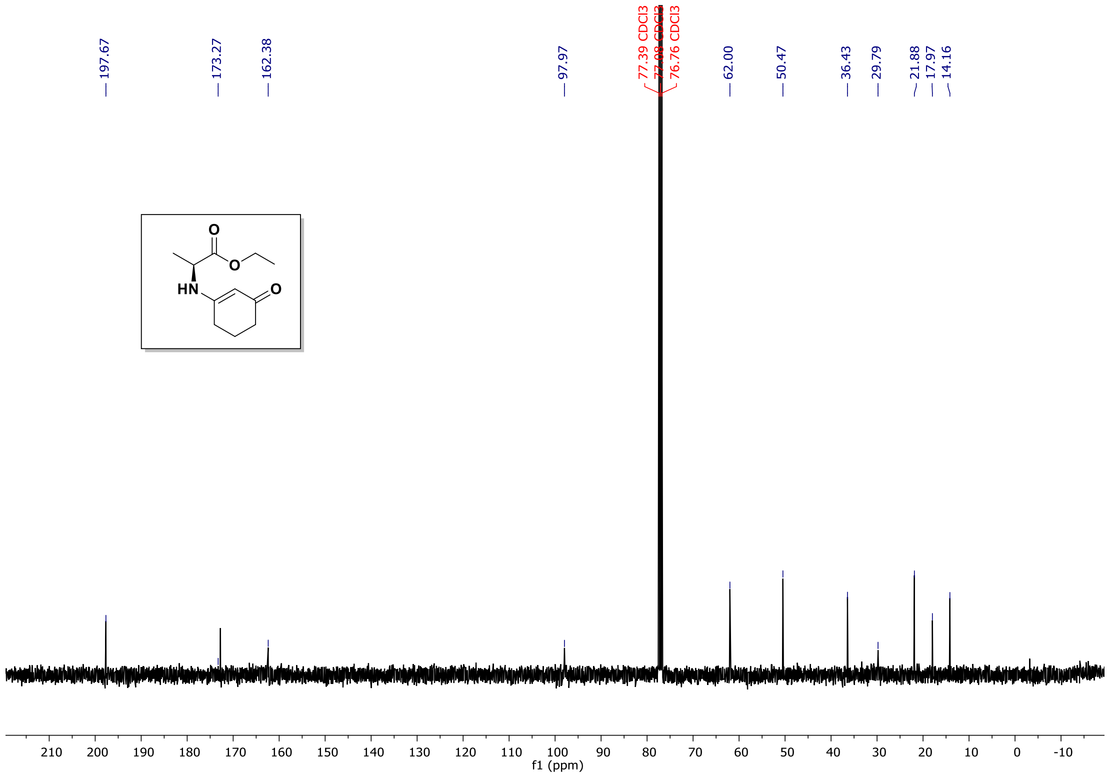

13C NMR spectrum of 46 in $CDCl_3$.

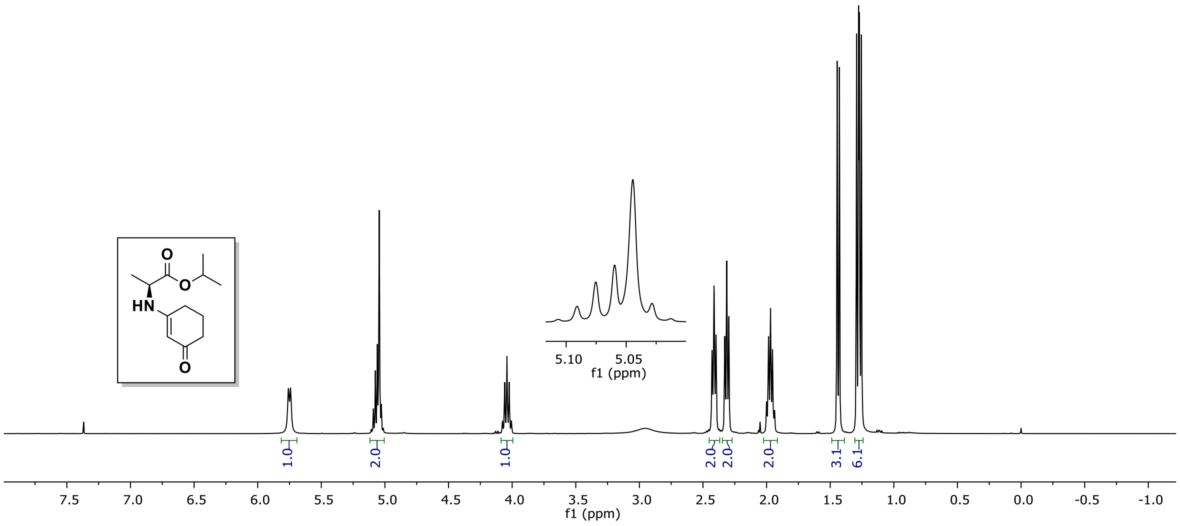

1H NMR spectrum of 47 in $CDCl_3$.

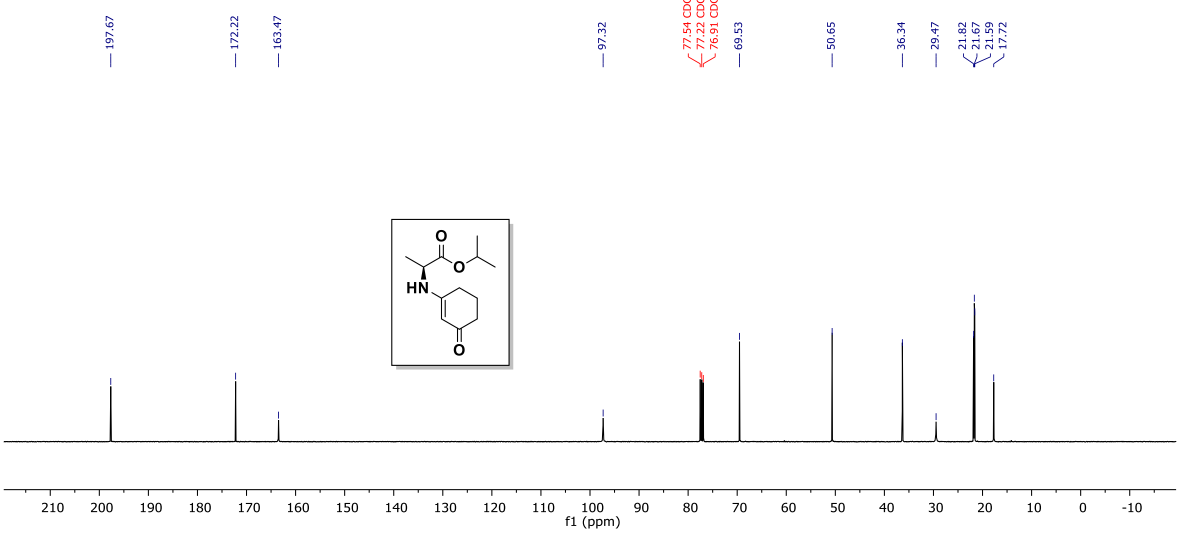

13C NMR spectrum of 47 in $CDCl_3$.

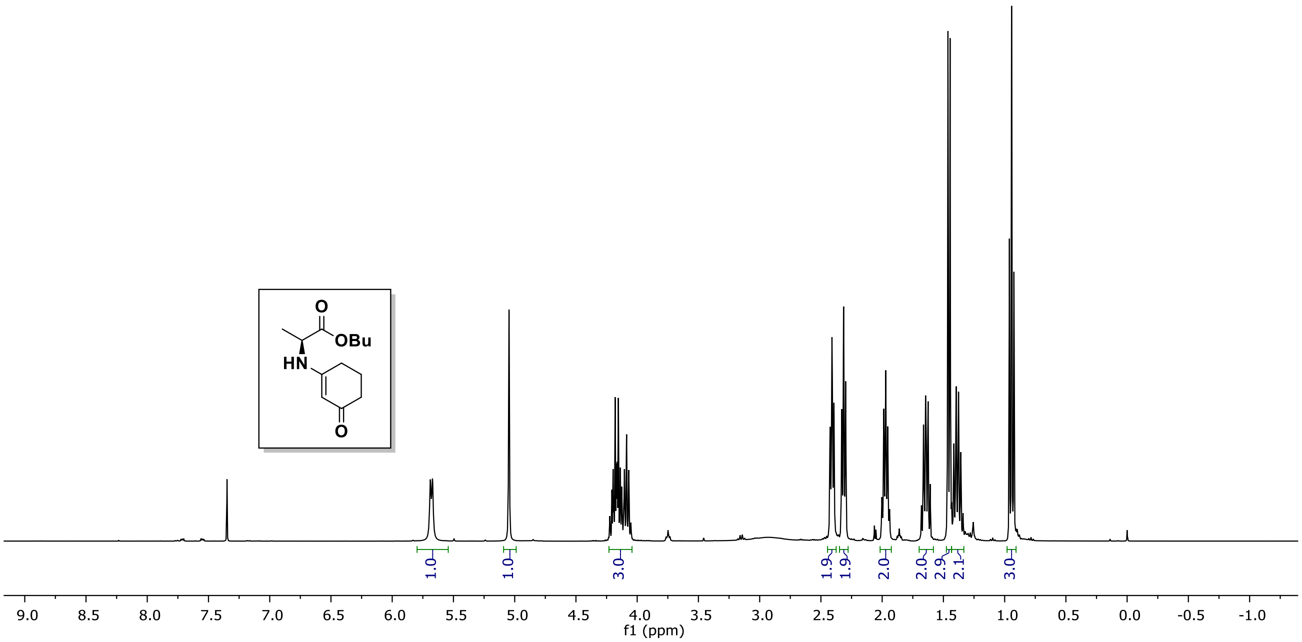

1H NMR spectrum of 48 in CDCl3.

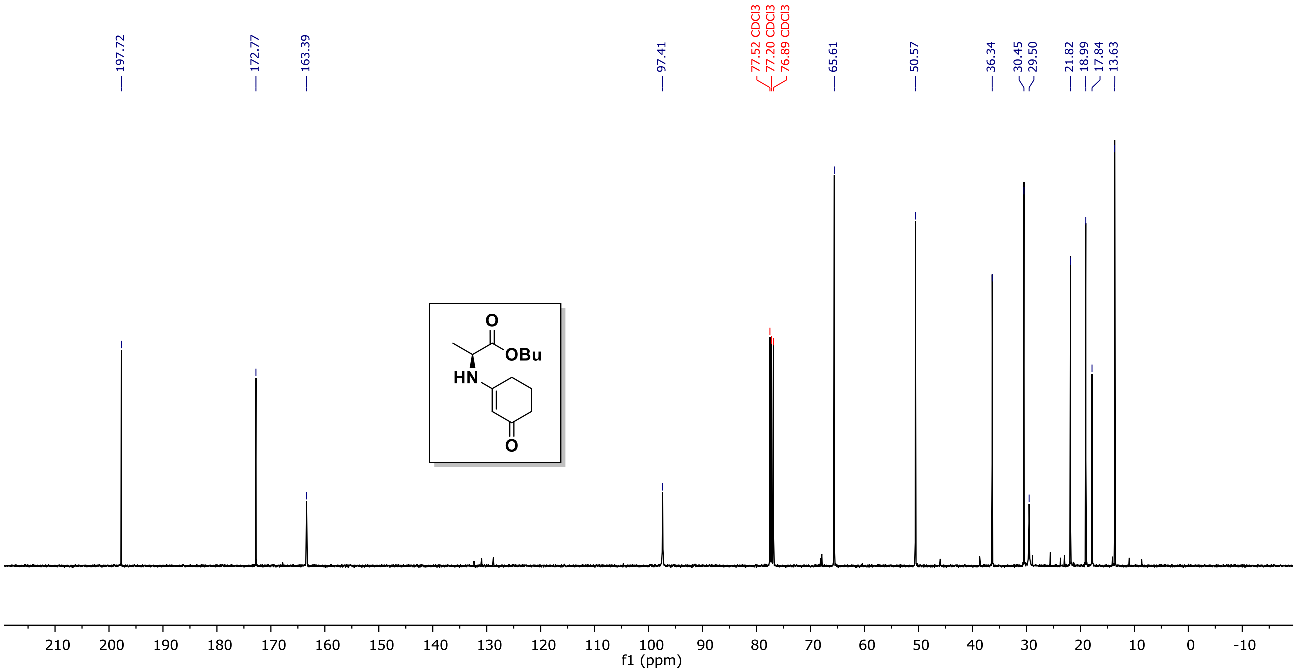

13C NMR spectrum of 48 in CDCl3.

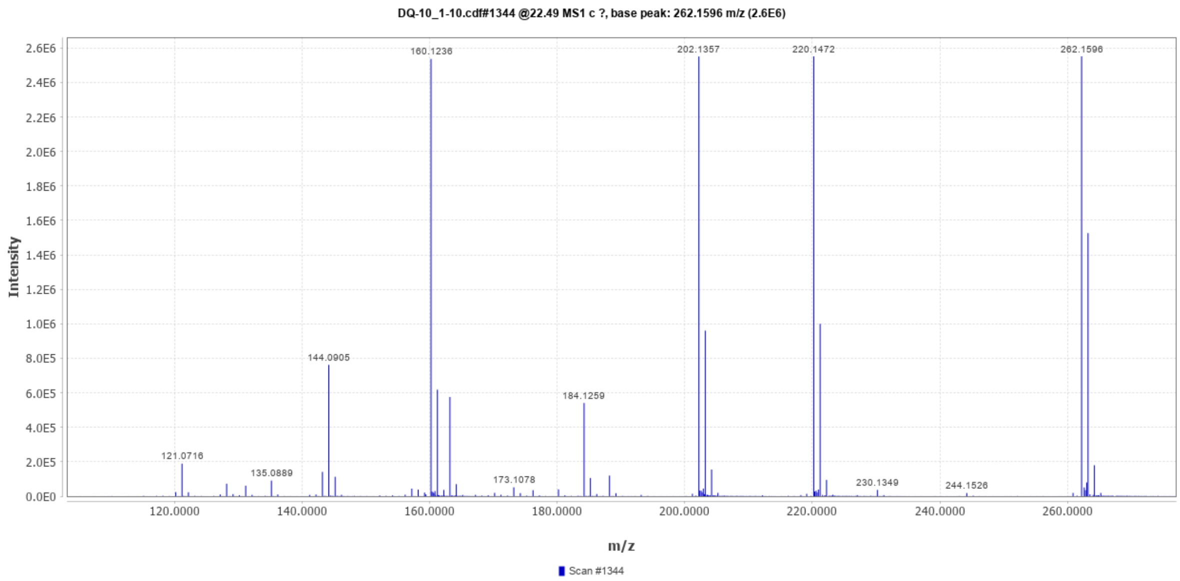

HRMS spectrum of 5

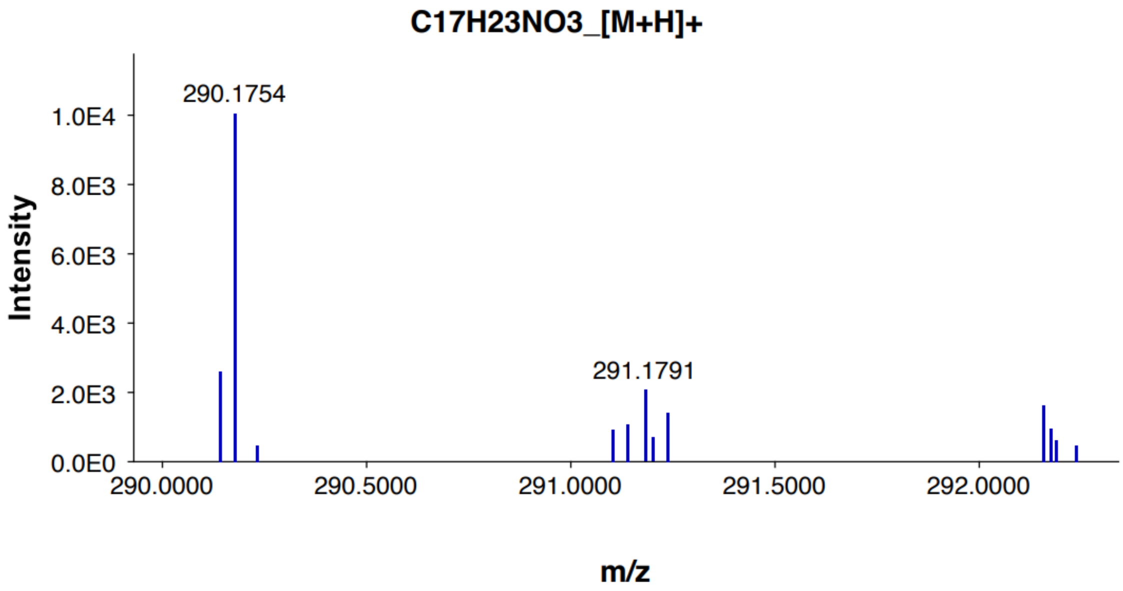

HRMS spectrum of 7

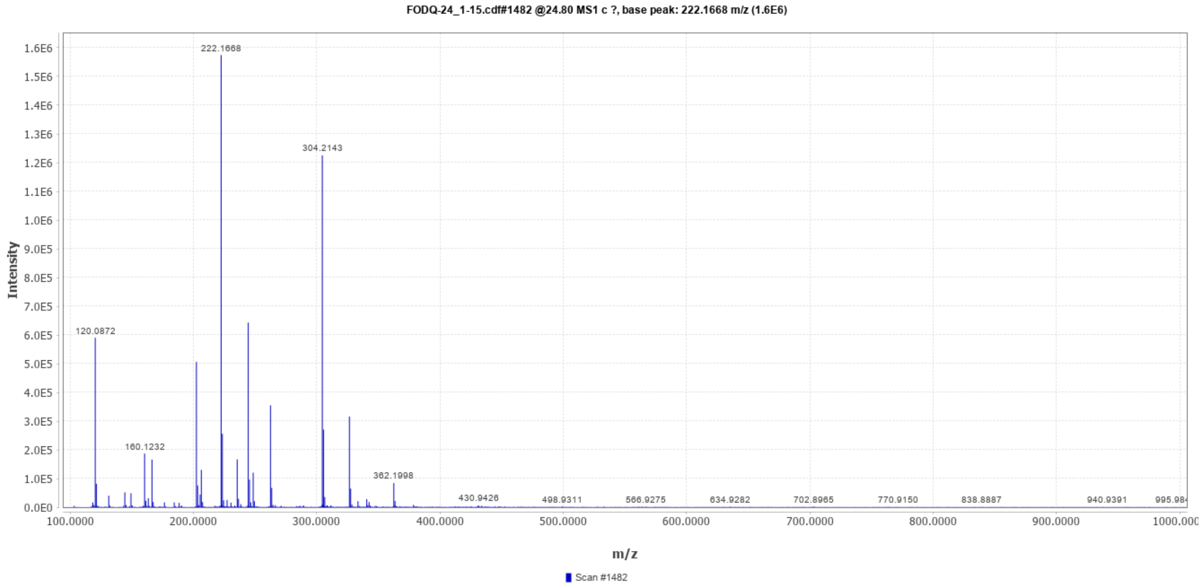

HRMS spectrum of 8

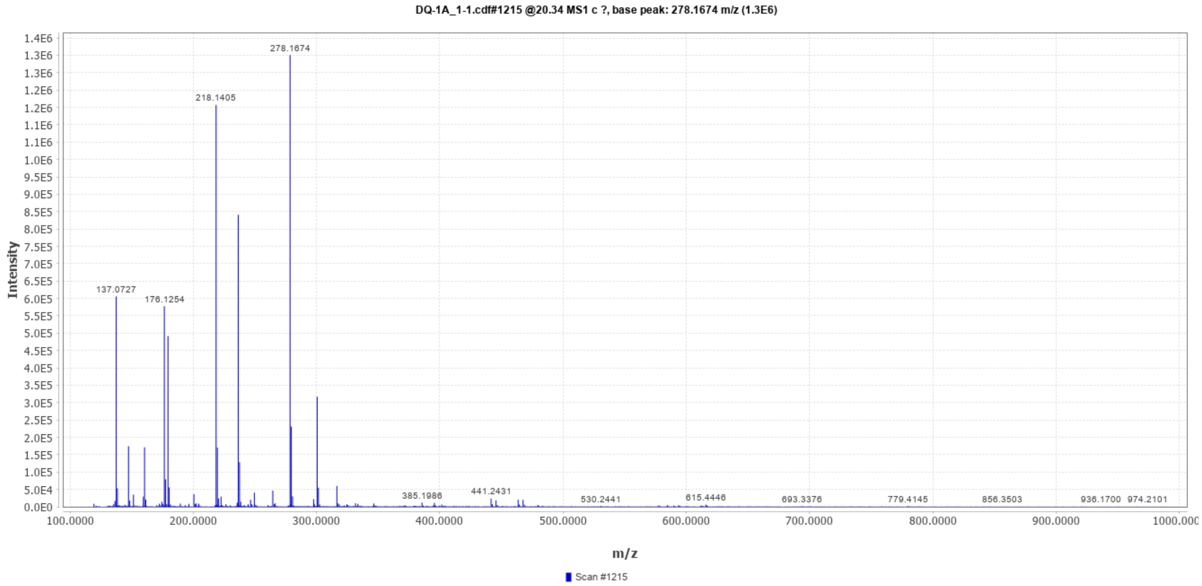

HRMS spectrum of 9

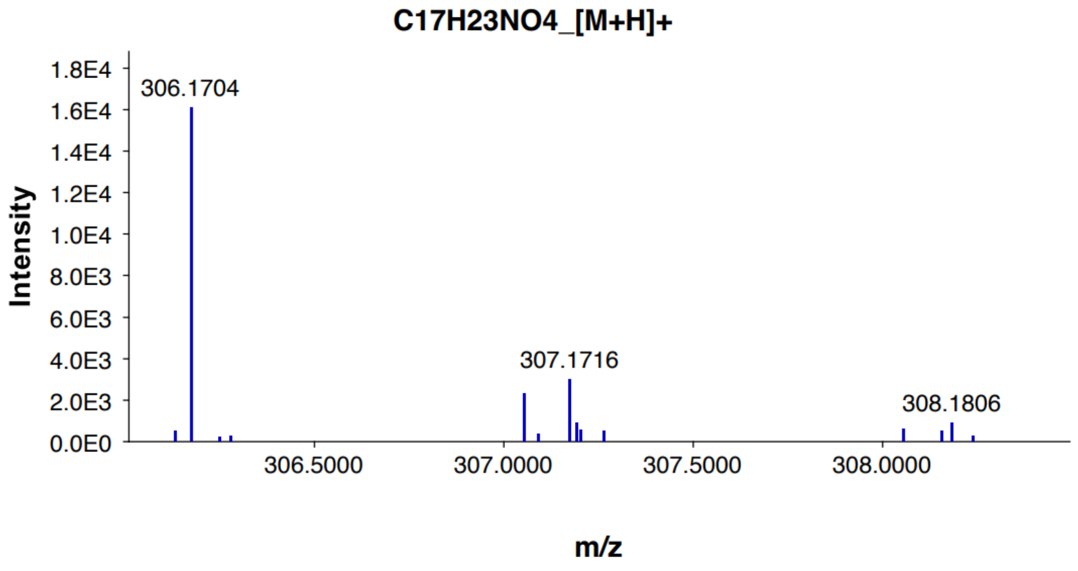

HRMS spectrum of 11

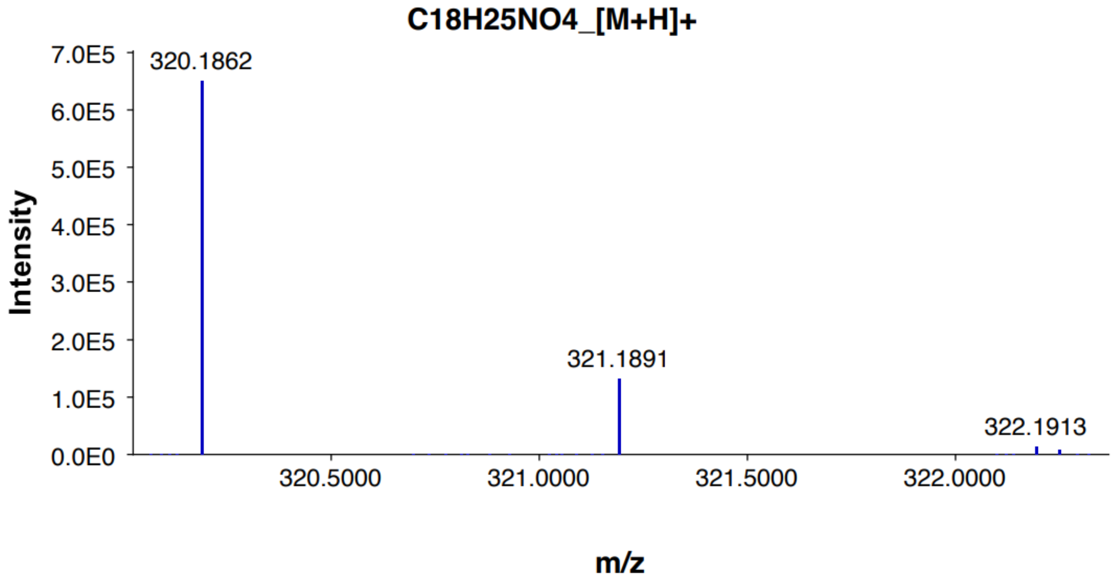

HRMS spectrum of 12.

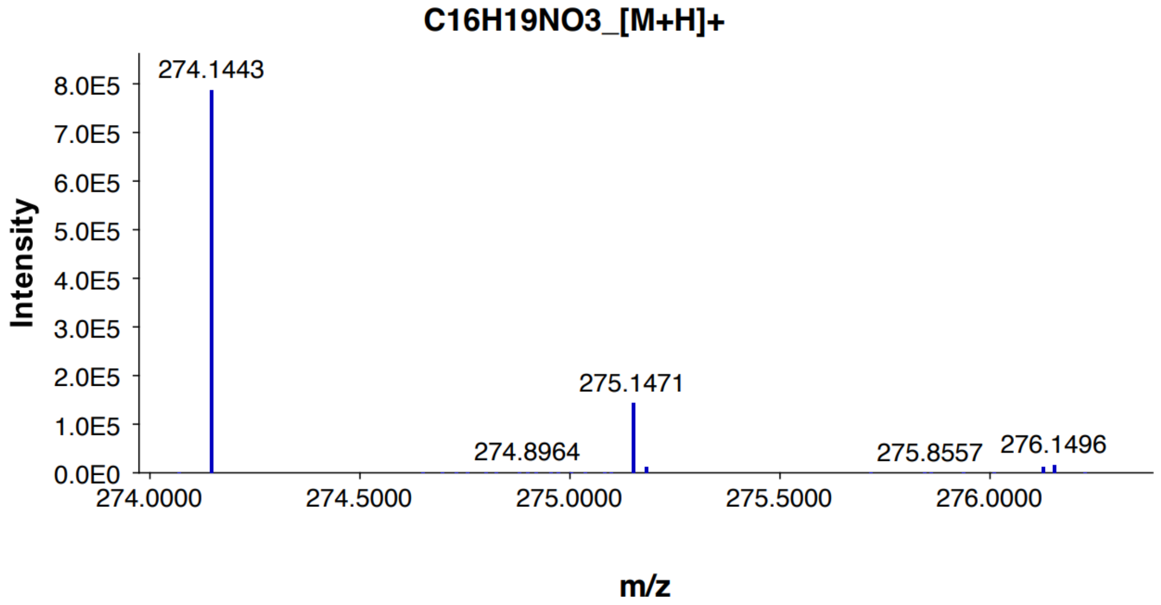

HRMS spectrum of 37.

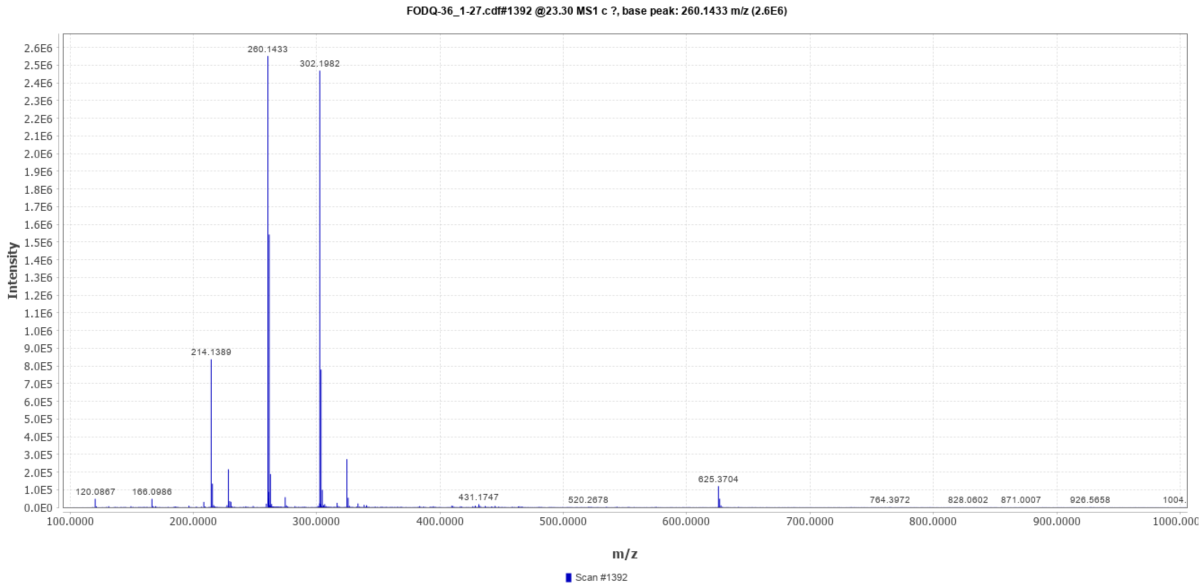

HRMS spectrum of 39

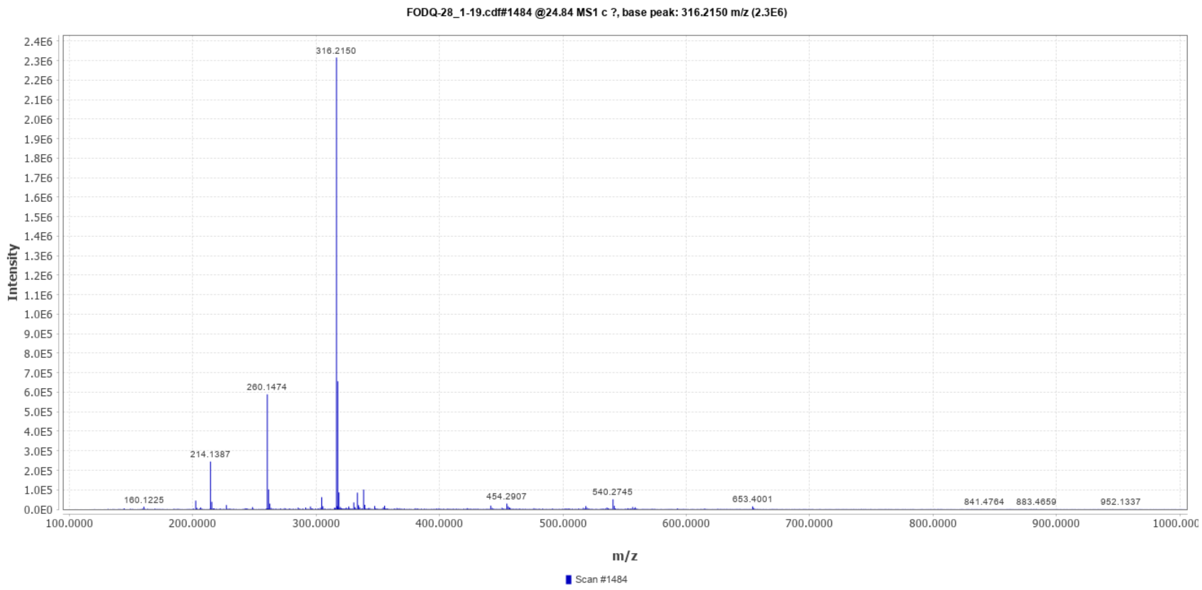

HRMS spectrum of 40

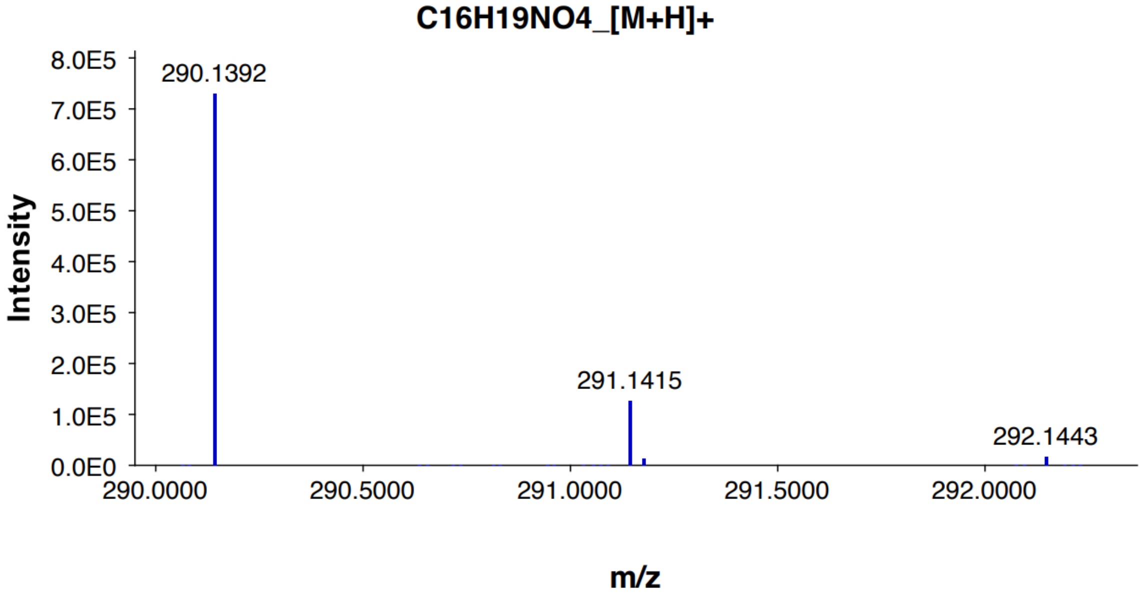

HRMS spectrum of 41

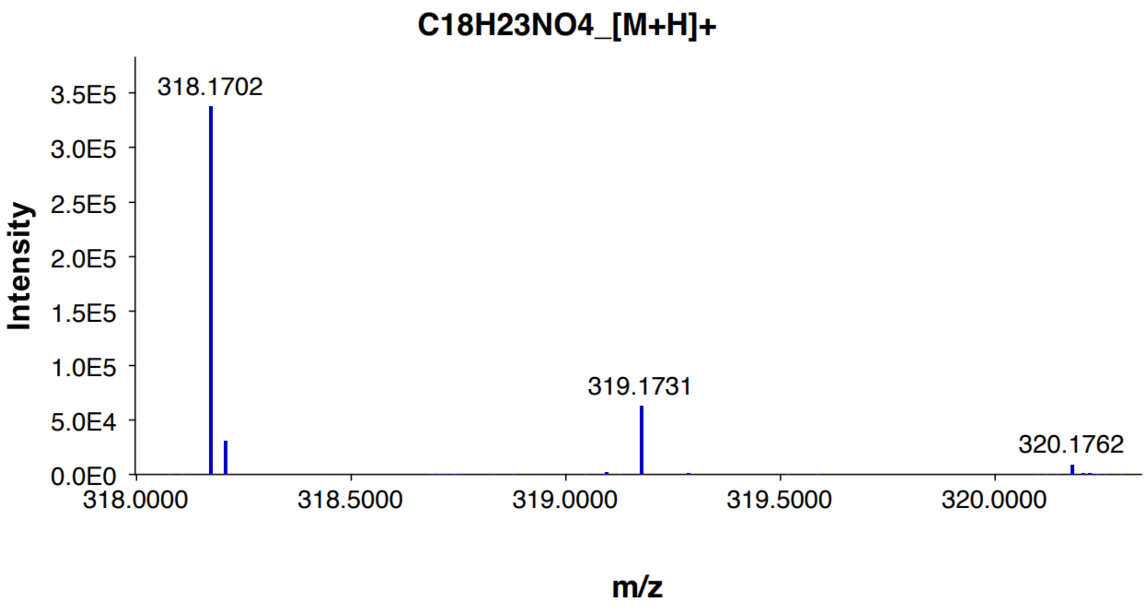

HRMS spectrum of 43

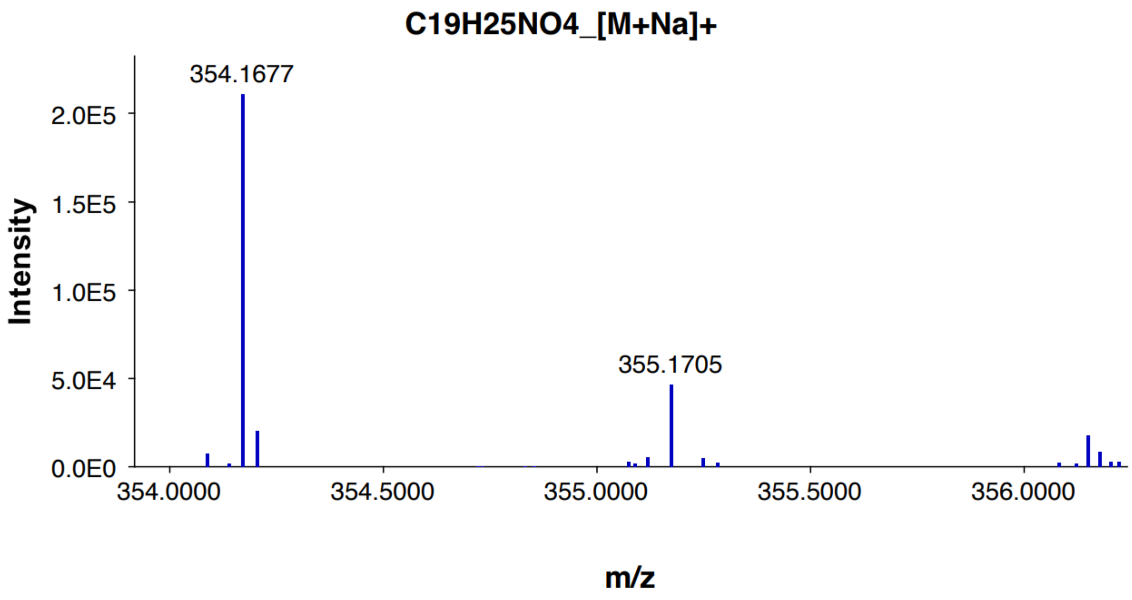

HRMS spectrum of 44
